# Inhibition of O‐GlcNAcylation protects from Shiga toxin‐mediated cell injury and lethality in host

**DOI:** 10.15252/emmm.202114678

**Published:** 2021-11-29

**Authors:** Kyung‐Soo Lee, Jieun Lee, Pureum Lee, Bong Chan Jeon, Min Yeong Song, Sojung Kwak, Jungwoon Lee, Jun‐Seob Kim, Doo‐Jin Kim, Ji Hyung Kim, Vernon L Tesh, Moo‐Seung Lee, Sung‐Kyun Park

**Affiliations:** ^1^ Environmental Diseases Research Center Korea Research Institute of Bioscience & Biotechnology (KRIBB) Daejeon Korea; ^2^ Department of Biomolecular Science KRIBB School of Bioscience Korea University of Science and Technology (UST) Daejeon Korea; ^3^ Immunotherapy Convergence Research Center Korea Research Institute of Bioscience & Biotechnology (KRIBB) Daejeon Korea; ^4^ Department of Nano‐Bioengineering Incheon National University Incheon Korea; ^5^ Infectious Disease Research Center Korea Research Institute of Bioscience & Biotechnology (KRIBB) Daejeon Korea; ^6^ Department of Microbial Pathogenesis and Immunology College of Medicine Texas A&M University Bryan TX USA

**Keywords:** apoptosis, hemolytic uremic syndrome, inflammation, O‐GlcNAcylation, Shiga toxin, Digestive System, Microbiology, Virology & Host Pathogen Interaction, Post-translational Modifications & Proteolysis

## Abstract

Shiga toxins (Stxs) produced by enterohemorrhagic *Escherichia coli* (EHEC) are the major virulence factors responsible for hemorrhagic colitis, which can lead to life‐threatening systemic complications including acute renal failure (hemolytic uremic syndrome) and neuropathy. Here, we report that O‐GlcNAcylation, a type of post‐translational modification, was acutely increased upon induction of endoplasmic reticulum (ER) stress in host cells by Stxs. Suppression of the abnormal Stx‐mediated increase in O‐GlcNAcylation effectively inhibited apoptotic and inflammatory responses in Stx‐susceptible cells. The protective effect of O‐GlcNAc inhibition for Stx‐mediated pathogenic responses was also verified using three‐dimensional (3D)‐cultured spheroids or organoids mimicking the human kidney. Treatment with an O‐GlcNAcylation inhibitor remarkably improved the major disease symptoms and survival rate for mice intraperitoneally injected with a lethal dose of Stx. In conclusion, this study elucidates O‐GlcNAcylation‐dependent pathogenic mechanisms of Stxs and demonstrates that inhibition of aberrant O‐GlcNAcylation is a potential approach to treat Stx‐mediated diseases.

## Graphical abstract

Here we identify a previously unknown link between the O‐GlcNAcylation pathway and the pathogenesis of enterohemorrhagic *E*. *coli* Shiga toxin‐mediated diseases. Given the capacity of these bacterial toxins to subvert normal cellular regulation in the host, this study demonstrates that Shiga toxins alter O‐GlcNAcylation, a type of post‐translational modification, to exacerbate dysfunction in host cell signaling.

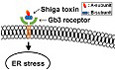

**The paper explained**

**Problem**

Shiga toxins (Stxs) produced by enterohemorrhagic *Escherichia coli* (EHEC) are particular public health concerns because of the potential to develop life‐threatening diseases such as bloody diarrhea, acute kidney dysfunction, and neurological abnormalities, primarily affecting children. Currently, there are no vaccines or specific and effective therapeutic strategies to prevent extraintestinal complications or mitigate the severity of renal injury.

**Results**

In the present study, we have identified an essential regulatory mechanism that controls Stx‐mediated HUS (hemolytic uremic syndrome) pathogenesis by global regulation of O‐GlcNAcylation (a type of post‐translational modification) involved in host damage. The O‐GlcNAcylation level in Stx‐exposed cells was elevated acutely, which promoted apoptotic and pro‐inflammatory responses. Activation of the ER stress response upon Stx intoxication bridged the pathways that induce enhanced O‐GlcNAcylation and host programmed cell death. In addition, O‐GlcNAcylation controlled Akt and p65 activity under these conditions, leading to modulation of Bad‐ and NF‐κB‐related pathways. Importantly, inhibiting O‐GlcNAcylation using a chemical inhibitor or OGT‐targeting siRNA decreased apoptosis and expression of pro‐inflammatory cytokines/chemokines. Furthermore, treatment with an OGT inhibitor reduced Stx2a‐mediated damage to human 3D‐kidney spheroids or organoids, and improved the survival rate and severe clinical symptoms of Stx2a‐injected mice.

**Impact**

Approaches to develop interventional strategies to treat the disease caused by Stxs have been based on our limited understanding of the interaction of the toxins with the host. Thus, the impact on the field of this study would be to not only increase our fundamental knowledge of the interaction of Stxs with host tissues but also provide the potential to identify targets for effective therapeutic regimens or interventions to ameliorate diseases mediated by the bacterial toxins.

## Introduction

Due to the successful development and use of antibiotics and effective public health measures, and improvements in health care delivery, epidemics of many bacterial infectious diseases are now considered a less serious risk. However, Shiga toxin (Stx)‐producing bacteria, particularly EHEC, have received considerable attention as emergent pathogens due to the harmful toxins they produce and their potential to cause widespread outbreaks of food‐borne disease (Majowicz *et al*, 2014). Shiga toxin was first isolated from the bacteria *Shigella dysenteriae* serotype 1. Shiga toxin‐producing *E. coli* (STEC) may express one or more structurally related but antigenically distinguishable toxin types, designated Shiga toxin type 1 (Stx1) and Shiga toxin type 2 (Stx2). Structurally and functionally related genetic variants of Stx1 (Stx1a–d) and Stx2 (Stx2a–g) have been identified (Melton‐Celsa, 2014). Stxs are multi‐functional ribosome‐inactivating proteins primarily responsible for the development of hemolytic uremic syndrome (HUS) and central nervous system impairment, life‐threatening complications that may follow diarrheal disease (Lee & Tesh, 2019). All Stxs consist of six protein subunits with an AB_5_ molecular configuration; the monomeric A subunit (˜32 kDa) possesses the toxin enzymatic activity and the homopentameric B subunits (7.7 kDa) are associated with binding to glycolipid receptors globotriaosylceramide (Gb_3_) and globotetraosylceramide (Gb_4_) (Fraser *et al*, 2004; Lingwood, 2020). Following Gb3 receptor binding, holotoxin molecules are internalized via mechanisms that are both clathrin dependent and clathrin independent. Stxs are subsequently trafficked in a retrograde manner through the trans‐Golgi network and Golgi apparatus to the endoplasmic reticulum (ER) (Khine *et al*, 2004; Romer *et al*, 2007; Sandvig *et al*, 2010). After entering the cytoplasm via the ER, which is the intracellular site for quality control and post‐translational processing of proteins, Stxs inhibit protein synthesis by modifying the 28S rRNA component of the ribosome and induce apoptosis by activating ER stress (Lee *et al*, 2008; Menge, 2020). Although the capacity of Stxs to trigger the phosphorylation of multiple host cellular proteins has been extensively demonstrated (Tesh, 2012), other Stx‐triggered post‐translational modifications (PTMs) that may exacerbate cytotoxicity, organ failure, or death of the host remain incompletely understood.

Proteins within the nucleocytoplasmic compartment are dynamically modified by the addition of O‐linked β‐N‐acetylglucosamine (O‐GlcNAc), derived from the end‐product uridine 5′‐phosphate (UDP)‐GlcNAc of the hexosamine biosynthetic pathway (HBP), to serine and threonine hydroxyl groups (Torres & Hart, 1984; Hart *et al*, 2007). This PTM is reversible, similar to phosphorylation. O‐GlcNAc transferase (OGT) and O‐GlcNAcase (OGA) are enzymes responsible for the addition and removal of O‐GlcNAc, respectively (Dong & Hart, 1994; Kreppel *et al*, 1997). In addition, crosstalk between phosphorylation and O‐GlcNAcylation has been extensively reported. Inhibition of a single kinase such as glycogen synthase kinase 3β significantly alters O‐GlcNAcylation of many cellular proteins and *vice versa* because protein O‐phosphorylation and O‐GlcNAcylation often occur competitively or reciprocally at the same site (Hart *et al*, 2011). Moreover, many kinases and phosphatases are major targets of O‐GlcNAcylation (Wang *et al*, 2008; Schwein & Woo, 2020). Due to this relationship between O‐GlcNAcylation and phosphorylation, the addition and removal of O‐GlcNAc directly or indirectly participate in a wide range of cellular processes such as gene expression, glycolysis, protein interactions, cell growth, insulin resistance, and stress responses via regulation of various target proteins (Hart *et al*, 2011; Bond & Hanover, 2013). Perturbation of the O‐GlcNAcylation pathway is highly associated with many human diseases including cancer, diabetes, Alzheimer’s disease, and cardiovascular disease (McClain, 2002; Marshall, 2006; Bond & Hanover, 2013; Wani *et al*, 2017; Wright *et al*, 2017). The α‐toxin of the pathogenic bacterium *Clostridium novyi* mimics OGT, despite lacking homology to the mammalian enzyme, and kills host cells by improperly attaching O‐GlcNAc to GTP‐binding sites in small GTPases (Selzer *et al*, 1996; Busch *et al*, 2000). In addition, mass spectrometry analysis revealed that flagellins of *Listeria monocytogenes* are modified with O‐GlcNAc (Schirm *et al*, 2004). Although only limited studies have investigated putative roles of O‐GlcNAcylation in infectious diseases, these observations suggest that the O‐GlcNAcylation pathway must be precisely regulated in host cells to properly control the spread of infectious agents or to reduce the detrimental effects of bacterial infections or toxins.

In this study, we identified a previously unknown link between the O‐GlcNAcylation pathway and the pathogenesis of Stx‐related diseases. O‐GlcNAcylation levels were acutely elevated in Stx‐exposed cells and elevation promoted apoptotic and pro‐inflammatory responses. Activation of the ER stress response by intoxication bridged the induction of enhanced O‐GlcNAcylation and host programmed cell death pathways. O‐GlcNAcylation controlled Akt and p65 activity under these conditions, leading to modulation of Bad‐ and NF‐κB‐related pathways. Inhibition of O‐GlcNAcylation using a chemical inhibitor or OGT‐targeting siRNA (siOGT) effectively decreased apoptosis and expression of pro‐inflammatory cytokines/chemokines. Treatment with an OGT inhibitor reduced Stx2a‐mediated damage of human 3D‐kidney spheroids or organoids and improved the survival rate and severe clinical symptoms of Stx2a‐injected mice. Our findings suggest that O‐GlcNAcylation is involved in activation of two major pathological mechanisms (apoptosis and inflammation) induced by Stxs and that a promising therapeutic agent to ameliorate the Stx‐mediated progression of HUS may be developed by inhibiting O‐GlcNAcylation.

## Results

### Stx exposure acutely increases the cellular O‐GlcNAc level by inducing ER stress

**Figure 1:**
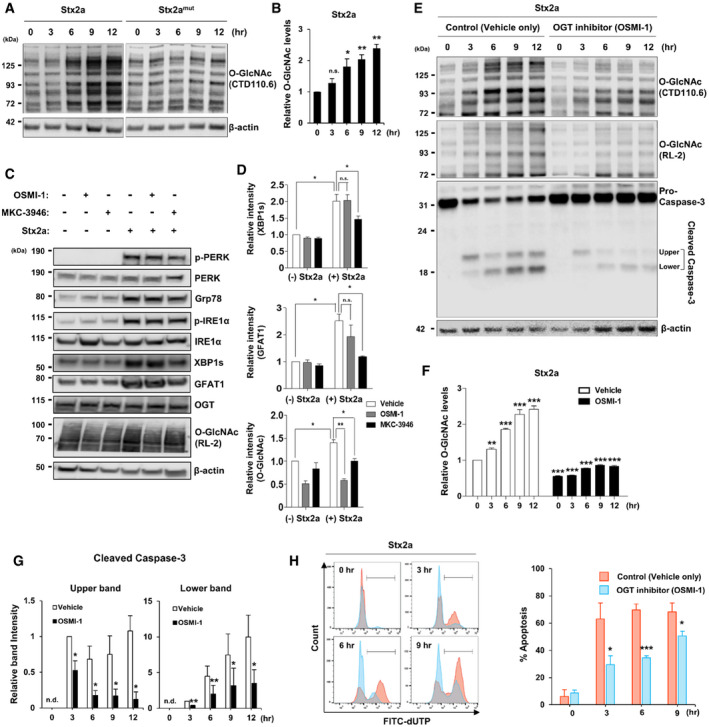
An acute increase in the cellular O‐GlcNAc level by the induction of ER stress upon Stx2a exposure causes apoptosis of THP‐1 cells Representative western blot showing changes in O‐GlcNAcylation in THP‐1 cells treated with Stx2a or Stx2a^mut^ (10 ng/ml each) for 0–12 h.Quantification of the band intensities in (A). Data are presented as mean ± SD (*n* = 3 biological replicates) normalized against β‐actin, which was used as a loading control.Representative western blot showing changes in various ER stress markers and O‐GlcNAcylation‐related protein expression in THP‐1 cells treated with Stx2a (10 ng/ml) for 3 h in the presence or absence of O‐linked *N*‐acetylglucosamine transferase (OGT) inhibitor OSMI‐1 (10 µM final) or serine/threonine‐protein kinase/endoribonuclease inositol‐requiring enzyme 1 a (IRE1a) inhibitor MKC‐3946 (10 µM final).Quantification of the band intensities in (C). Data are presented as mean ± SEM (*n* = 3 biological replicates) normalized against β‐actin, which was used as a loading control.Representative western blot of O‐GlcNAcylation and pro‐caspase‐3 cleavage in THP‐1 cells treated with Stx2a (10 ng/ml) in the presence or absence of OGT inhibitor OSMI‐1 (10 µM final).Quantification of the band intensities for O‐GlcNAcylation (RL‐2) in (E). Data are presented as mean ± SEM (*n* = 3 biological replicates) normalized against β‐actin, which was used as a loading control. The effects of Stx2a‐mediated induction for O‐GlcNAc levels at each time point were compared to 0 h (left panel), and OSMI‐1 treatment was compared with that of the vehicle (DMSO) control at each time point (right panel).Quantification of the relative band intensities of cleaved caspase‐3 in (E) (Left panel: upper band; Right panel: lower band). Data are presented as mean ± SD (*n* = 3 biological replicates) normalized against β‐actin, which was used as a loading control. The effects of OSMI‐1 were compared with those of the vehicle (DMSO) control at each time point. (n.d. = not detected).Left panel: Representative flow cytometric plots showing apoptosis of THP‐1 cells detected by the TUNEL assay upon treatment with Stx2a (10 ng/ml) in the presence (blue) or absence (red) of OSMI‐1 (10 µM, final). Right panel: Quantification of the percentage of apoptotic cells at each time point derived from the plots presented in the upper panel. Data are presented as mean ± SD (*n* = 3 biological replicates). The effects of OSMI‐1 were compared with those of the vehicle (DMSO) control at each time point. Data information: Statistical analysis was performed using two‐tailed Student’s *t*‐test. *P < 0.05; **P < 0.01; and ***P < 0.001. Source data are available online for this figure.

**Figure EV1:**
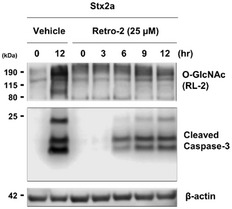
Inhibition of retrograde trafficking blocks the aberrant Stx2a‐induced increase in cellular O‐GlcNAcylation and consequently delays caspase‐3 activation Representative western blot showing changes in O‐GlcNAcylation and caspase‐3 activation in THP‐1 cells treated with Stx2a (10 ng/ml) for 12 h in the presence or absence of the retrograde trafficking inhibitor Retro‐2 (25 µM final). Source data are available online for this figure.

To explore the potential role of O‐GlcNAcylation in the pathogenesis of Stx‐related diseases, we examined changes in the cellular O‐GlcNAc level upon Stx2a treatment. Exposure to Stx2a significantly increased total O‐GlcNAcylation in Stx‐sensitive monocytic THP‐1 cells in a time‐dependent manner (Fig 1A, left panel, and 1B). It is well established that toxin molecules are internalized and transported into the ER via the Golgi apparatus in host cells, and subsequently trigger ER stress by inducing an accumulation of unfolded proteins (Lee *et al*, 2008), which in turn may initiate potentially fatal cellular processes such as pro‐apoptotic responses and pro‐inflammatory cytokine production (Lee & Tesh, 2019). On the other hand, ER stress could be correlated with the O‐GlcNAcylation pathway as previously reported (Zachara *et al*, 2004; Qiu *et al*, 2005). Therefore, we investigated the correlation between ER stress and induction of O‐GlcNAcylation in Stx‐exposed cells. In contrast to wild‐type form of Stx2a, the O‐GlcNAcylation level was barely induced in cells exposed to an enzymatic mutant form of Stx2a (Stx2a^mut^ ), which triggers a very limited ER stress response (Lee *et al*, 2008; Park *et al*, 2017a) (Fig 1A, right panel). In addition, intoxication with Stx2a did not elevate the O‐GlcNAc level in THP‐1 cells in the presence of Retro‐2, which prevents ER stress by inhibiting retrograde trafficking of Stxs from the Golgi to the ER (Secher *et al*, 2015) (Fig EV1, upper panel). Interestingly, it was previously reported that glutamine fructose‐6‐phosphate aminotransferase 1 (GFAT1), the rate‐limiting enzyme of HBP upstream of O‐GlcNAcylation, is upregulated due to an increase in the spliced form of X‐box binding protein 1 (XBP1) under ER stress condition (Wang *et al*, 2014). To examine if this mechanism can be related directly to ER stress‐mediated induction of O‐GlcNAcylation upon Stx exposure, cells were co‐treated with an inhibitor of the serine/threonine protein kinase/endoribonuclease inositol‐requiring enzyme 1 a (IRE1a), which is known to regulate XBP1 splicing (Yoshida *et al*, 2001). As expected, treatment with Stx2a alone induced various ER stress markers such as protein kinase RNA‐like ER kinase (PERK), 78‐kDa glucose‐regulated protein (Grp78), IRE1a, and consequently the spliced form of XBP1 (XBP1s) (Fig 1C). However, under treatment of IRE1α inhibitor MKC‐3946 (Mimura *et al*, 2012), XBP1s were decreased, thereby reducing the expression of GFAT1 and then inhibiting O‐GlcNAcylation (Fig 1C and D). In addition, when OSMI‐1, a potent inhibitor of OGT, was used (Ortiz‐Meoz *et al*, 2015; Park *et al*, 2017b), the abnormally increased O‐GlcNAcylation level by Stx exposure could be efficiently reduced, but there was no effect on the expression of ER stress‐related proteins or GFAT1 (Fig 1C and D). Considering that there is no change in OGT expression upon Stx exposure (Fig 1C), it is assumed that O‐GlcNAc induction by host cells during Stx intoxication is associated with the acute increase in GFAT1 expression, which presumably upregulates the production of UDP‐GlcNAc, a substrate of O‐GlcNAcylation, via HBP. Collectively, these results suggest that increased O‐GlcNAcylation is involved in ER stress‐mediated pathogenic cellular responses following intoxication mediated by Stx2a.

### Inhibition of O‐GlcNAcylation effectively delays Stx‐induced apoptosis of host cells

**Figure EV2:**
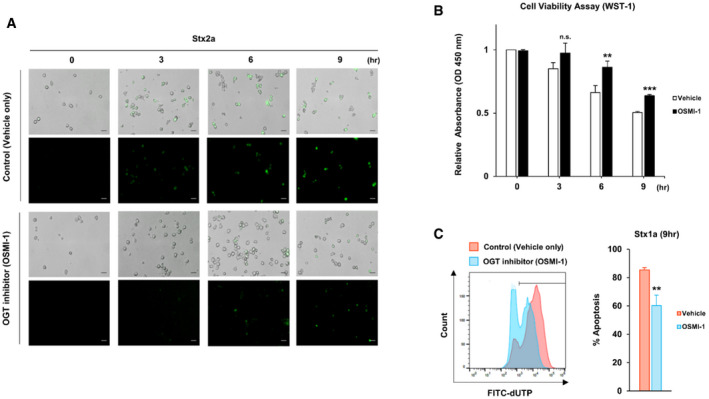
Suppression of Stx2a‐ and Stx1a‐induced O‐GlcNAcylation rescues cells from apoptosis Representative images showing TUNEL staining of Stx2a‐treated THP‐1 cells cultured in the presence of OSMI‐1 or the vehicle control at 0, 3, 6, and 9 h time point each. FITC‐dUTP staining (green fluorescence) in the TUNEL assay indicates active progression of apoptosis. Scale bars: 40 µm.WST‐1 dye‐based cell viability assay of Stx2a‐exposed THP‐1 cells treated with or without OSMI‐1 (10 µM final) at 0, 3, 6, and 9 h time point each (*n* = 3 biological replicates).Representative flow cytometric plot showing apoptosis progression in THP‐1 cells detected by TUNEL upon treatment with Stx1a (100 ng/ml) in the presence or absence of OSMI‐1 (10 µM final), and quantification of the percentage of apoptotic cells at 9 h (*n* = 3 biological replicates). The effects of OSMI‐1 treatment were compared with those of the vehicle (DMSO) control. Data information: Error bars for bar graphs are presented as mean ± SD Statistical analysis was performed using two‐tailed Student’s *t*‐test. *P < 0.05; **P < 0.01; and ***P < 0.001. Source data are available online for this figure.

We demonstrated that an acute increase in O‐GlcNAcylation was associated with the ER stress response upon Stx2a exposure. Therefore, we hypothesized that inhibition of the abnormal increase in O‐GlcNAcylation would modulate several pathological pathways activated under ER stress. To test this hypothesis, we first investigated whether the inhibitor of OGT, OSMI‐1, which showed the effect of efficiently reducing the abrupt induction of O‐GlcNAcylation in Stx2a‐exposed cells (Fig 1C), could downregulate Stx‐mediated apoptosis of host cells. Exposure to Stx2a rapidly increased O‐GlcNAcylation in vehicle‐treated THP‐1 cells after only 3 h; however, pre‐treatment with 10 µM OSMI‐1 for 12 h followed by exposure to Stx2a effectively reduced the increase in O‐GlcNAcylation for up to 12 h (Fig 1E, upper panel and 1F). Cleavage of pro‐caspase‐3, which occurred following induction of O‐GlcNAcylation upon Stx2a exposure, was also decreased in the presence of OSMI‐1 (Fig 1E and G). In addition, Retro‐2 treatment, which inhibits the increase in O‐GlcNAc level through reducing ER stress response by inhibiting toxin retro‐trafficking, also hindered pro‐caspase‐3 cleavage similarly to treatment with OGT inhibitors (Fig EV1), suggesting that O‐GlcNAcylation may be involved in the regulation of ER stress‐mediated apoptosis of host cells by Stxs. Therefore, we analyzed apoptosis by terminal deoxynucleotidyl transferase (TdT) deoxyuridine triphosphate (dUTP) nick end‐labeling (TUNEL) assay. DNA fragmentation occurs in apoptotic cells and, consequently, these cells can be identified and quantified via fluorescence‐activated cell sorting (FACS) or fluorescence microscopy following labeling of the 3′‐hydroxyl termini of DNA double‐strand breaks with fluorescently tagged dUTP using the TdT enzyme (Darzynkiewicz *et al*, 2008). Observation of fluorescent cells under a microscope revealed that OSMI‐1 treatment reduced the number of apoptotic cells upon Stx2a exposure, as expected (Fig EV2A). Quantitative analysis of fluorescent cells following TUNEL via FACS demonstrated that OSMI‐1 treatment significantly delayed apoptosis of Stx2a‐exposed cells (Fig 1H). In addition, the inhibitory effects of OSMI‐1 on Stx2a‐induced apoptosis were further validated by the WST‐1 dye‐based cell viability assay (Fig EV2B). Furthermore, O‐GlcNAcylation was similar induced when THP‐1 cells were treated with Stx1a, which is a different toxin type (data not shown), and OSMI‐1 treatment rescued apoptosis of Stx1a‐exposed cells (Fig EV2C). Because cells were pre‐treated with OSMI‐1 for 12 h before Stx exposure, no increase in either cleaved caspase‐3 (Fig 1E and G) or fluorescent cells of the TUNEL assay (Fig 1H) at 0 h time point under Stx2a plus OSMI‐1 conditions indicated that the treatment of OSMI‐1 alone had no significant effect on inducing apoptosis. Based on these results, we conclude that inhibition of O‐GlcNAcylation substantially delays Stx1a‐ and Stx2a‐induced apoptosis of host cells.

### Inhibition of O‐GlcNAcylation significantly decreases Stx‐induced production of pro‐inflammatory cytokines and chemokines in host cells

**Figure 2:**
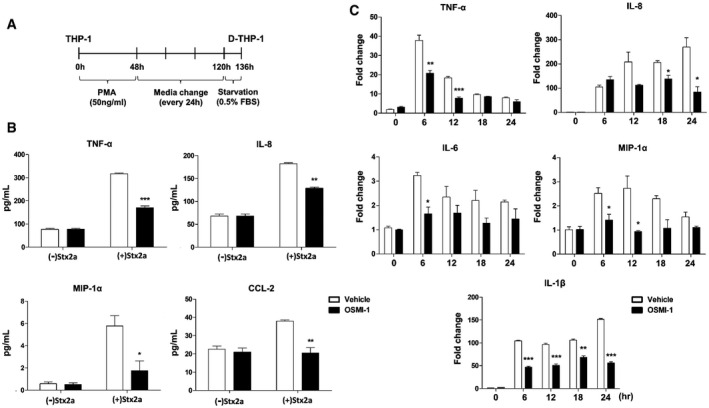
Suppression of O‐GlcNAcylation downregulates pro‐inflammatory cytokine/chemokine production induced by Stx2a intoxication Timeline for establishment of differentiated macrophage‐like THP‐1 cells (D‐THP‐1) from monocytic THP‐1 cells.ELISAs were used to analyze the inhibitory effect of OSMI‐1 (10 µM, final) on cytokine and chemokine production from THP‐1 cells exposed to Stx2a (10 ng/ml) for 9 h (*n* = 3 biological replicates).Real‐time qRT‐PCR analyses of fold changes in transcript levels for each cytokine/chemokine gene in D‐THP‐1 cells following Stx2a (10 ng/ml) intoxication for various time points in the presence or absence of OSMI‐1 (10 µM, final). Data are normalized using GAPDH as a loading control (*n* = 3 biological replicates). Data information: For graphs in (B and C), error bar represents mean ± SEM. Statistical analysis was performed using two‐tailed Student’s *t*‐test. *P < 0.05; **P < 0.01; and ***P < 0.001. The effects of OSMI‐1 were compared with those of the vehicle (DMSO) control at each time point. Source data are available online for this figure.

O‐GlcNAcylation inhibition delayed apoptosis of host cells activated by ER stress upon Stx exposure. Based on this observation, we hypothesized that O‐GlcNAcylation may also regulate the inflammatory response, another pathogenic mechanism induced by ER stress. To systematically investigate whether O‐GlcNAc inhibition attenuated Stx‐induced increases in the expression of genes encoding pro‐inflammatory cytokines and chemokines, undifferentiated monocytic THP‐1 cells and differentiated macrophage‐like THP‐1 (D‐THP‐1) cells (Fig 2A), which are well established as an *in vitro* human macrophage model to characterize toxin‐regulated inflammatory cytokine and chemokine production (Leyva‐Illades *et al*, 2012; Lee *et al*, 2016b), were treated with Stx2a in the presence or absence of OGT inhibitor OSMI‐1. As we hypothesized, enzyme‐linked immunosorbent assays (ELISAs) and quantitative reverse transcription PCR (RT‐qPCR) demonstrated that O‐GlcNAc inhibition considerably downregulated expression of pro‐inflammatory cytokines and chemokines such as tumor necrosis factor (TNF)‐α, interleukin (IL)‐8 [C‐X‐C motif chemokine ligand 8 (CXCL8)], IL‐1β, macrophage inflammatory protein (MIP)‐1α, and chemokine (C‐C motif) ligand (CCL)‐2 in THP‐1 (Fig 2B) as well as D‐THP‐1 cells (Fig 2C) following intoxication with Stx2a. There was no significant effect on cytokine induction by the treatment of OSMI‐1 alone in both types of cells. Taken together, these data demonstrate that the pro‐inflammatory response activated under ER stress in Stx2a‐exposed host cells is effectively suppressed by inhibiting the rapid increase in O‐GlcNAcylation.

### Increased O‐GlcNAcylation in Stx‐exposed host cells directly regulates phosphorylation of Akt and p65, which modulate the activities of Bad/caspase‐ or NF‐κB‐related pathways to induce apoptosis or inflammation, respectively

**Figure 3:**
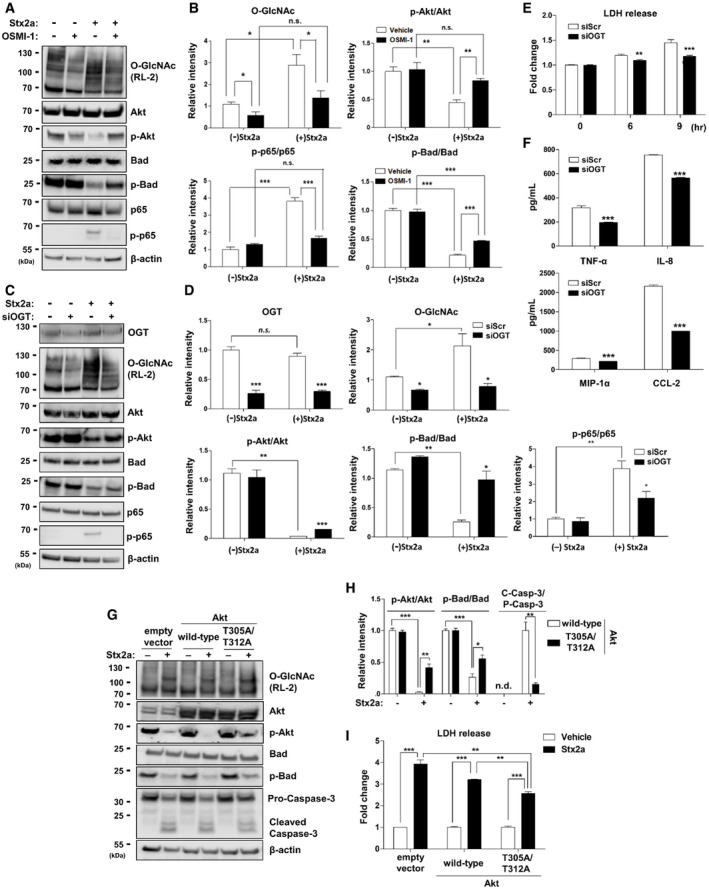
Inhibition of O‐GlcNAcylation restricts the activation of apoptotic and pro‐inflammatory responses caused by Stx2a through directly influencing the phosphorylation status of Akt and p65 Representative western blot showing changes in overall O‐GlcNAcylation (upper panel) or phosphorylation status of each target protein (lower panels) in THP‐1 cells treated with Stx2a (10 ng/ml) for 9 h in the presence or absence of OSMI‐1 (10 µM, final).Quantification of the relative band intensities for O‐GlcNAcylation (RL‐2) or each of the phosphorylated and total protein bands in (A). Data are normalized against β‐actin, which was used as a loading control (*n* = 3 biological replicates). The effects of Stx2a in vehicle controls were compared with lysates prepared from cells maintained in the absence of Stx2a, and OSMI‐1 treatment was compared with that of the vehicle (DMSO) controls in the presence of Stx2a.Representative western blot showing changes in OGT expression (top panel), overall O‐GlcNAcylation (second panel), or phosphorylation status of each target protein (lower panels) in THP‐1 cells treated with Stx2a (10 ng/ml) for 9 h in the presence or absence of OGT knockdown.Quantification of the relative band intensities between each of phosphorylated and total protein bands in (C). Data are normalized against β‐actin, which was used as a loading control (*n* = 3 biological replicates). The effects of Stx2a in siScr controls were compared with those of lysates from cells maintained in the absence of Stx2a, and OGT knockdown was compared with that of siScr controls in the presence of Stx2a (n.s. = not significant).LDH cytotoxicity assay performed during 9 h culture period following Stx2a (10 ng/ml) treatment of THP‐1 cells in the presence or absence of OGT knockdown (*n* = 3 biological replicates). The effects of OGT siRNA (siOGT) were compared with those of scrambled siRNA (siScr) controls at each time point.ELISAs were used to analyze the inhibitory effect of siOGT on cytokine/chemokine production from THP‐1 cells exposed to Stx2a (10 ng/ml) for 9 h (*n* = 3 biological replicates). The effects of OGT knockdown were compared with those of siScr controls in the presence of Stx2a.Representative western blot showing changes in overall O‐GlcNAcylation (upper panel), phosphorylation status of each target protein (middle panels), or pro‐caspase‐3 cleavage (lower panel) in THP‐1 cells treated with Stx2a (10 ng/ml) for 9 h in the presence or absence of overexpression of Akt (wild type or T305A/T312A).Quantification of the relative band intensities of each of the phosphorylated and total protein bands or cleaved caspase‐3 in (G). Data are normalized against β‐actin, which was used as a loading control (*n* = 3 biological replicates). The effects of Stx2a in vehicle controls were compared with lysates prepared from cells maintained in the absence of Stx2a, and overexpression of mutant Akt (T305A/T312A) was compared with that of Akt_wild‐type in the presence of Stx2a (n.d. = not detected; C‐Casp‐3 = Cleaved Caspase‐3; P‐Casp‐3 = Pro‐Caspase‐3).LDH cytotoxicity assay performed at 9 h following Stx2a (10 ng/ml) treatment of THP‐1 cells in the presence or absence of overexpression of Akt (wild type or T305A/T312A) (*n* = 3 biological replicates). The effects of Stx2a were compared with those of culture medium from cells transfected with Akt (wild type or T305A/T312A), and overexpression of mutant Akt (T305A/T312A) was compared with that of Akt_wild type in the presence of Stx2a. Data information: Error bars for bar graphs are presented as mean ± SEM. Statistical analysis was performed using two‐tailed Student’s *t*‐test. *P < 0.05; **P < 0.01; and ***P < 0.001. Source data are available online for this figure.

**Figure EV3:**
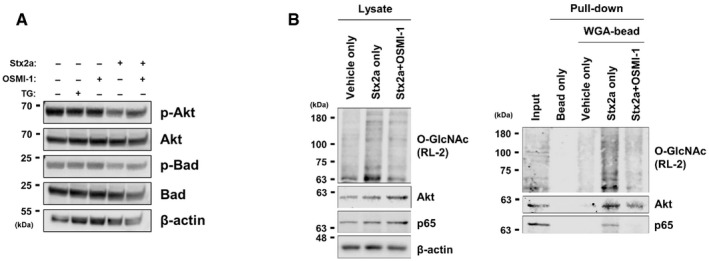
Akt and p65 were directly O‐GlcNAcylated in Stx2a‐treated THP‐1 cells Representative western blot showing changes in phosphorylation status of Akt or Bad in THP‐1 cells treated with Stx2a (10 ng/ml) for 3 h in the presence or absence of OSMI‐1 (10 µM, final) or OGA inhibitor Thiamet G (2 µM, final).Representative western blot images, before (left) and after (right) pull down using WGA‐lectin conjugated to agarose beads or bead‐only control, to determine O‐GlcNAc attachment to Akt and p65 in lysates from Stx2a (10 ng/ml)‐exposed THP‐1 cells for 9 h in the presence or absence of OSMI‐1 (10 µM, final). Source data are available online for this figure.

To provide mechanistic data supporting our novel finding that ER stress rapidly and abnormally elevates O‐GlcNAcylation in Stx‐exposed host cells, we sought to identify proteins that connect O‐GlcNAcylation and pathogenic changes induced by Stxs. Among several proteins linked with the pathogenesis of Stx‐related diseases, Akt was a prime candidate because not only is its activity known to be under the control of O‐GlcNAcylation but also it is a critical regulator of both apoptosis and the inflammatory reaction (Datta *et al*, 1999; Vergadi *et al*, 2017; Schwein & Woo, 2020). Moreover, Cherla *et al* (2009) demonstrated that Akt activity negatively regulates Stx1a‐induced production of pro‐inflammatory cytokines. To determine whether the increase in O‐GlcNAcylation upon Stx exposure affected Akt activity, we checked the phosphorylation status of Akt and its downstream proteins. As anticipated, the acute increase in O‐GlcNAcylation in Stx‐treated host cells led to a significant reduction in Akt phosphorylation at threonine 308 (Fig 3A and B), but not at serine 473 (data not shown). Consequently, phosphorylation of Bad, which is part of a canonical signaling pathway mediating apoptosis under the control of Akt, was also decreased (Fig 3A and B). Notably, since these changes in phosphorylation level of Akt or Bad already occurred at 3 h after Stx2a exposure (Fig EV3A), as early as caspase‐3 was shown to be activated (Fig 1E and G), it is presumed that the apoptotic response was acutely mediated through those proteins. Contrary to Akt activation, phosphorylation at serine 536 of p65, a main component of the NF‐κB pathway, was markedly increased upon O‐GlcNAc induction in Stx‐exposed cells (Fig 3A and B). It was reported that phosphorylation of p65 at serine 536 allows its transport into the nucleus, where it induces transcriptional activation of genes encoding pro‐inflammatory cytokines (Sakurai *et al*, 1999; Sasaki *et al*, 2005). Therefore, increased O‐GlcNAcylation appears to lead to the activation of apoptotic and inflammatory signals by regulating the phosphorylation status of several key proteins that have been shown to be involved in Stx pathogenesis. However, the alteration of phosphorylation status for Akt and p65 upon Stx exposure was effectively restored when the abnormal increase in O‐GlcNAcylation was prevented by treatment with OGT inhibitor OSMI‐1 (Fig 3A and B). Phosphorylation of Bad was also recovered (Fig 3A and B), thereby suppressing the increase in caspase activity (Fig 1E and G). Furthermore, Akt and p65 were clearly detected in proteins pull downed using a GlcNAc‐specific lectin (WGA), demonstrating that Akt and p65 were directly O‐GlcNAcylated in Stx2a‐treated THP‐1 cells (Fig EV3B), as previously reported in other cell systems (Jozwiak *et al*, 2014).

In order to verify the rescue effects of a chemical inhibitor of O‐GlcNAcylation in Stx‐treated cells, we utilized an OGT knockdown experiment to specifically downregulate OGT expression. First, it was clearly confirmed that transfection of siRNA for OGT (siOGT) into THP‐1 cells effectively inhibited OGT expression, thereby reducing overall O‐GlcNAc levels compared to transfection with scrambled siRNA (siScr) (Fig 3C and D). Under this condition, it was shown that Stx2a‐induced cytotoxicity was suppressed by OGT knockdown using a lactate dehydrogenase (LDH)‐based cell viability assay (Fig 3E). In addition, pro‐inflammatory cytokine and chemokine production upon Stx2a exposure in THP‐1 cells was significantly decreased through the inhibition of OGT expression by siRNA (Fig 3F). Interestingly, it was confirmed that phosphorylation changes in some key proteins such as Akt, Bad, or p65 following Stx2a treatment were recovered to reduce apoptosis or inflammatory reactions under OGT knockdown conditions similar to the use of chemical inhibitors (Fig 3C and D). Based on these results, we conclude that Stx‐induced apoptosis and inflammatory responses can be simultaneously suppressed by O‐GlcNAc inhibition via specific control of Akt‐ and p65‐related signaling pathways.

It has been reported that phosphorylation of Akt at threonine 308 is regulated depending on its O‐GlcNAcylation status at threonine 305 and 312 (Wang *et al*, 2012). Therefore, in order to verify whether this correlation affects Akt activity under conditions in which O‐GlcNAc levels are induced by Stx exposure, a mutant construct of Akt in which both threonine 305 and 312 (Akt_T305A/T312A) were substituted with alanine was compared to wild‐type Akt. When the wild type was overexpressed in THP‐1 cells, phosphorylation of Akt at threonine 308 was significantly reduced by increased O‐GlcNAcylation upon Stx2a exposure, which induced LDH release due to cell apoptosis (Fig 3G, H and I). However, in case of overexpression of Akt_T305A/T312A, the severe decrease in phosphorylation at threonine 308 was recovered, thereby reducing the amount of LDH release from Stx2a‐exposed cells (Fig 3G, H and I). Thus, these results directly support that increased O‐GlcNAcylation of Akt at threonine 305 and 312 under Stx exposure downregulates Akt activity by reducing phosphorylation at threonine 308.

### Stx2a‐activated pathogenic responses are regulated through the induction of O‐GlcNAc levels in primary human renal cells

**Figure 4:**
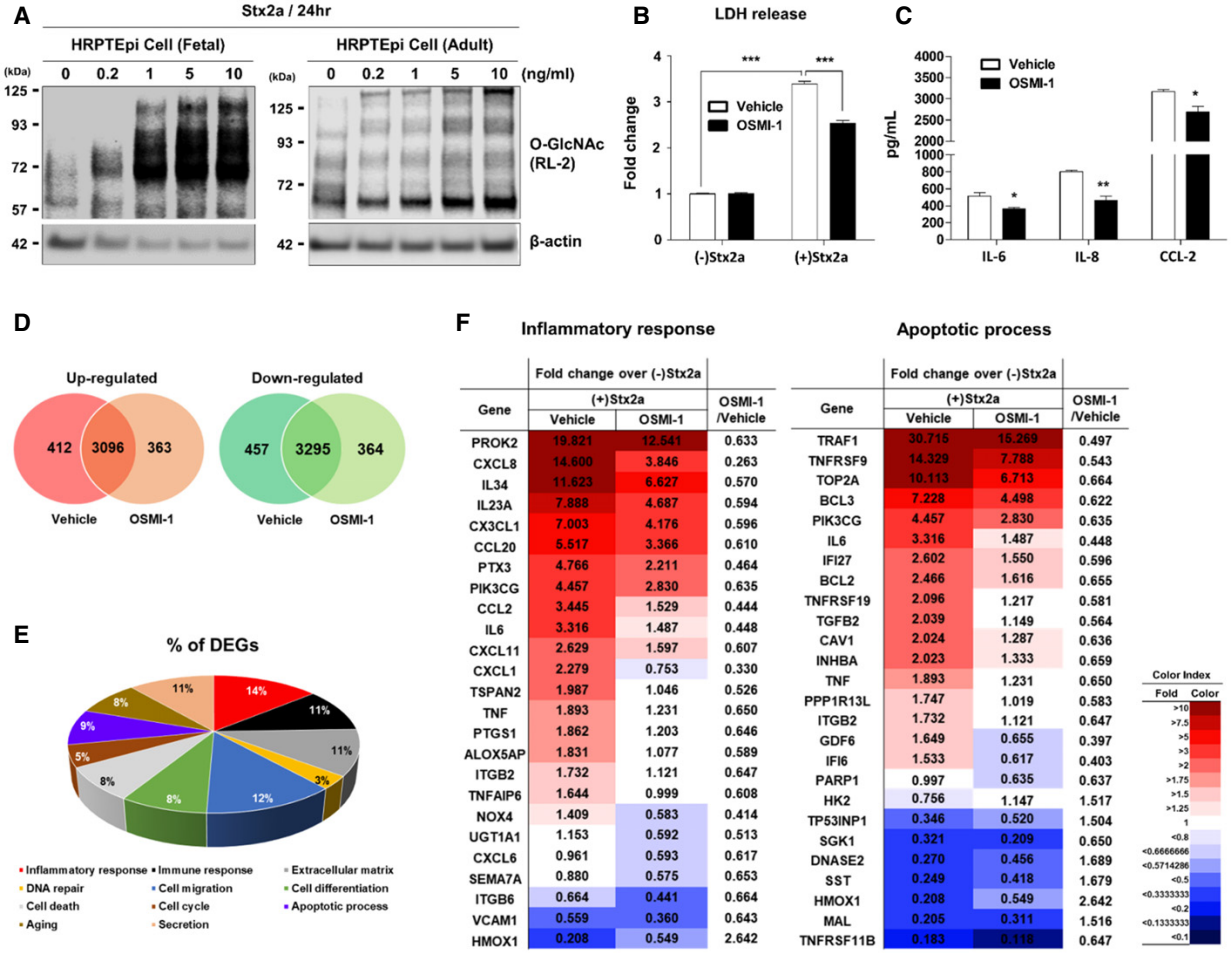
Stx2a‐mediated apoptotic and pro‐inflammatory responses in primary human renal cells are regulated through O‐GlcNAcylation ARepresentative western blot showing the changes in O‐GlcNAcylation in primary human renal proximal tubule epithelial (HRPTEpi) cells (originating from fetal or adult kidney) treated with different concentrations (0–10 ng/ml) of Stx2a for 24 h.BLDH cytotoxicity assay following Stx2a (10 ng/ml) treatment of HRPTEpi (adult) cells for 48 h in the presence or absence OSMI‐1 (10 µM, final) (*n* = 3 biological replicates). The effects of Stx2a in vehicle controls were compared with culture medium prepared from HRPTEpi cells maintained in the absence of Stx2a, and OSMI‐1 treatment was compared with that of the vehicle (DMSO) controls in the presence of Stx2a.CELISAs were used to analyze the inhibitory effect of OSMI‐1 on cytokine/chemokine production from HRPTEpi cells (adult) exposed to Stx2a (10 ng/ml) for 48 h (*n* = 3 biological replicates). The effects of OSMI‐1 were compared with those of the vehicle (DMSO) control.D–FGlobal transcriptome analysis of differentially expressed genes of HRPTEpi cells during Stx2a intoxication with vehicle or 10 µM of OSMI‐1, respectively. (D) Venn diagram of overlapping differentially expressed genes (DEGs) in the presence or absence of OSMI‐1 treatment among Stx2a‐inducible genes compared to cells cultured without Stx2a. (E) Pie chart showing genes significantly regulated by OSMI‐1 in the Stx2a‐exposed HRPTEpi cells as categorized by function. (F) Heatmaps representing the comparative expression levels for DEGs in the presence or absence of OSMI‐1 treatment upon Stx2a intoxication related to the inflammatory response (left) and apoptotic process (right). The numbers within the tables are normalized gene expression level compared to HRPTEpi cells maintained in the absence of Stx2a. Expression values are represented with red (upregulation) or blue (downregulation) color using FPKM values by Cufflinks; the cutoffs used a fold change of at least 1.5 followed by pairwise comparison and Student’s *t*‐test with a Benjamini and Hochberg correction. The FPKM values were normalized using EdgeR within R and visualized using ExDEGA. Data are the means from two independent replicates. Data information: For graphs in (B and C), error bar represents mean ± SEM. Statistical analysis was performed using two‐tailed Student’s *t*‐test. *P < 0.05; **P < 0.01; and ***P < 0.001. Source data are available online for this figure.

**Figure EV4:**
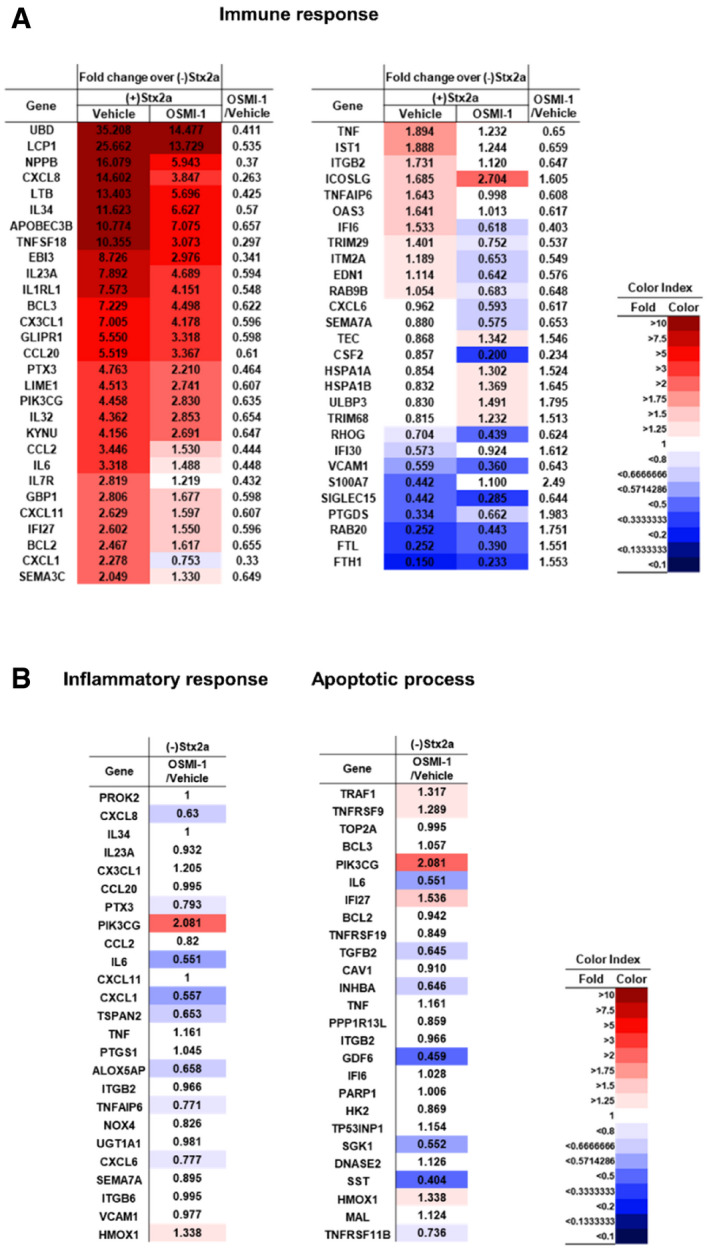
Stx2a‐mediated immune responses in primary human renal cells are regulated through O‐GlcNAcylation Heatmaps representing the comparative expression levels for DEGs in the presence or absence of OSMI‐1 treatment upon Stx2a intoxication related to the immune responses.Heatmaps representing the comparative expression levels for DEGs in the presence or absence of OSMI‐1 treatment without Stx2a exposure related to the inflammatory response (left) and apoptotic process (right). Data information: The numbers within the tables are normalized gene expression level compared to HRPTEpi cells maintained in the absence of either Stx2a or OSMI‐1. Expression values are represented with red (upregulation) or blue (downregulation) color using FPKM values by Cufflinks; the cutoffs used a fold change of at least 1.5 followed by pairwise comparison and Student’s *t*‐test with a Benjamini and Hochberg correction. The FPKM values were normalized using EdgeR within R and visualized using ExDEGA. Data are the means from two independent replicates.

Since the renal proximal tubule epithelial cell is a primary site of nephrotoxicity due to Stx‐activated pathogenic mechanisms in humans, we examined the role of O‐GlcNAcylation on pathways associated with apoptosis and inflammation in primary human renal proximal tubular epithelial (HRPTEpi) cells upon Stx2a exposure. As we initially characterized using Stx‐treated THP‐1 cells, treatment with Stx2a increased total O‐GlcNAcylation in HRPTEpi cells in a dose‐dependent manner (Fig 4A). In addition, downregulation of O‐GlcNAcylation using OGT inhibitor OSMI‐1 prominently suppressed apoptotic LDH release or inflammatory cytokine/chemokine production in HRPTEpi cells as well (Fig 4B and C), which implies that O‐GlcNAcylation‐related pathogenic activation by Stxs may be conserved in Stx‐sensitive cells. In order to globally analyze the rescue effect of OSMI‐1, we carried out transcriptome analysis using high‐throughput RNA sequencing to determine genes involved in O‐GlcNAcylation‐mediated regulatory mechanism of Stx*‐*intoxicated HRPTEpi cells. The total RNA fractions purified from the Stx2a‐treated HRPTEpi cells in the presence or absence of OSMI‐1 were analyzed to determine the respective gene expression levels. A total number of 7987 differentially expressed genes (DEGs) were identified which includes 3871 genes up‐regulated and 4116 genes down‐regulated upon Stx2a exposure, and most of the confirmed DEGs appeared in common regardless of OSMI‐1 treatment (Fig 4D; Dataset EV1). Functional enrichment analysis for those overlapped DEGs defined a number of significantly dysregulated gene ontology (GO) terms and Kyoto Encyclopedia of Genes and Genomes (KEGG) pathways, such as inflammation, cell migration, immune response, and apoptosis, under inhibition of O‐GlcNAcylation (Fig 4E). To demonstrate differentially expressed patterns in the presence or absence of OSMI‐1 upon Stx2a exposure, the comparative expression levels were displayed as heatmaps side by side across the conditions. As anticipated, the significant inhibitory effect of OSMI‐1 treatment was associated with the regulation of various genes involved in signaling pathways mainly associated with apoptosis, inflammation, and immune responses (Figs 4F and EV4A), but no influence by the treatment of OSMI‐1 alone (Fig EV4B; Dataset EV1). These results reinforce the concept that activation of Stx‐related pathogenic mechanisms can be significantly controlled via O‐GlcNAcylation.

### Hindrance of O‐GlcNAcylation protects against Stx2a‐mediated cytotoxicity and pro‐inflammatory response in three‐dimensional (3D) human mini‐kidney spheroids and induced pluripotent stem cells (iPSC)‐derived renal organoids

**Figure 5:**
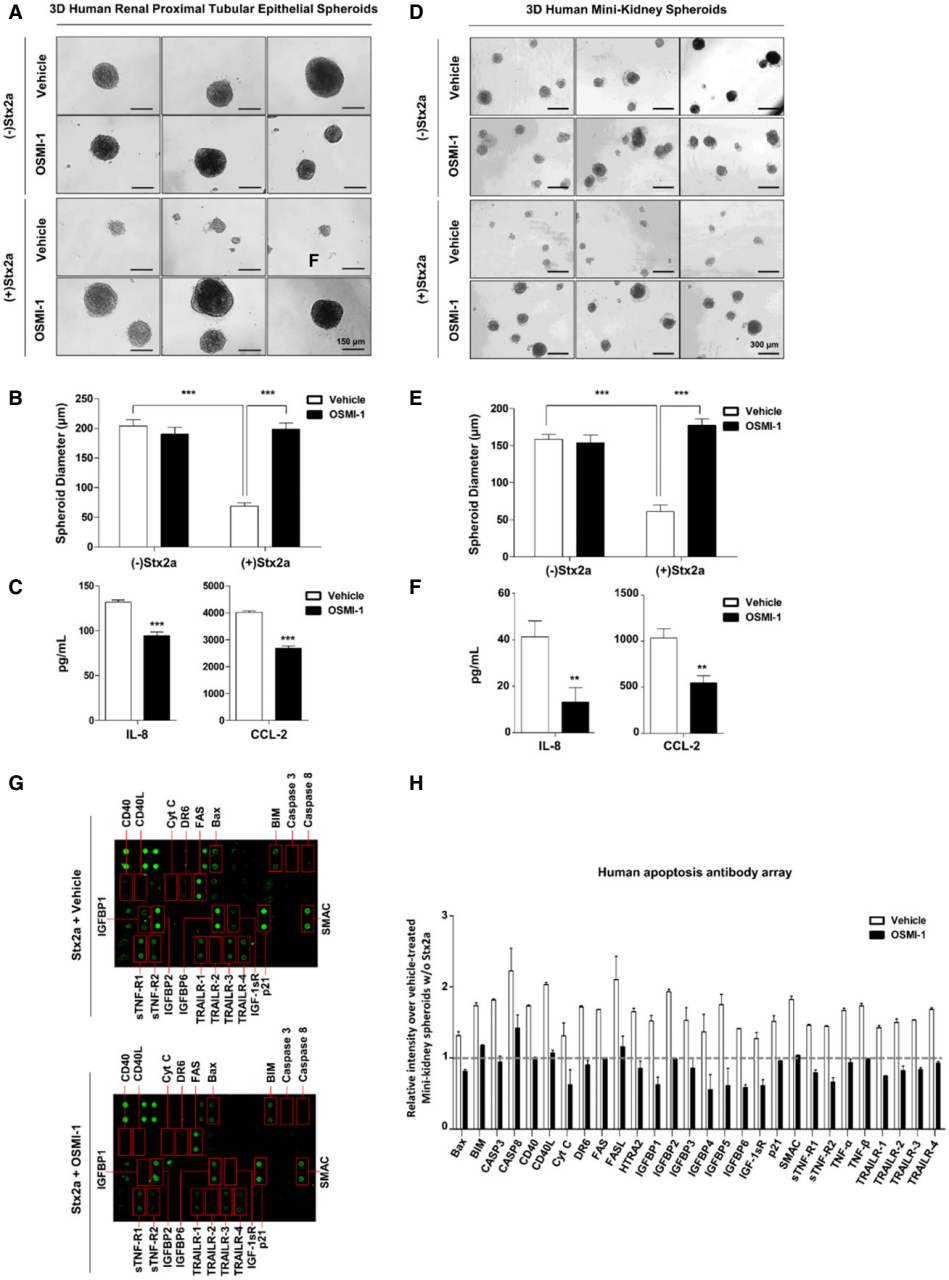
Protection from Stx2a‐induced apoptotic and inflammatory responses in 3D renal spheroids by inhibition of O‐GlcNAcylation ARepresentative images showing morphological changes in spheroids from 3D cultures of HRPTEpi cells following Stx2a (10 ng/ml) treatment for 72 h in the presence or absence OSMI‐1 (10 µM, final).BQuantification of estimated spheroid diameter in (A) [*n* = 11 independent spheroids (biological replicates)]. The effects of Stx2a in vehicle controls were compared with those of spheroids maintained in the absence of Stx2a, and OSMI‐1 treatment was compared with that of the vehicle (DMSO) controls in the presence of Stx2a.CELISAs were used to analyze the inhibitory effect of OSMI‐1 on IL‐8 and CCL‐2 production from 3D cultures of HRPTEpi cells exposed to Stx2a (10 ng/ml) for 72 h (*n* = 3 biological replicates). The effects of OSMI‐1 were compared with those of the vehicle (DMSO) controls.DRepresentative images showing morphological changes in spheroids from 3D cultures of human mini‐kidneys following Stx2a (10 ng/ml) treatment for 72 h in the presence or absence OSMI‐1 (10 µM, final).EQuantification of estimated spheroid diameter in (D) [*n* = 11 independent spheroids (biological replicates)]. The effects of Stx2a in vehicle controls were compared with those of spheroids maintained in the absence of Stx2a, and OSMI‐1 treatment was compared with that of the vehicle (DMSO) controls in the presence of Stx2a.FELISAs were used to analyze the inhibitory effect of OSMI‐1 on IL‐8 and CCL‐2 production from 3D cultures of human mini‐kidneys exposed to Stx2a (10 ng/ml) for 72 h (*n* = 3 biological replicates). The effects of OSMI‐1 were compared with those of the vehicle (DMSO) controls.G, HHuman apoptosis antibody array analysis of multiple proteins using pooled lysate from 3D‐human mini‐kidney spheroids following Stx2a (10 ng/ml) treatment for 72 h in the presence or absence of OSMI‐1 (10 µM, final). (G) Antibody spots representing signal differences are indicated in red boxes. (H) The graph shows the average of relative spot intensities for each protein compared to those measured in the control in the absence of Stx2a exposure (*n* = 2 biological replicates). Dashed line represents the reference point of the fold change. Raw values of fluorescence intensities are provided in the Source Data. Data information: Error bars for bar graphs are presented as mean ± SEM. Statistical analysis was performed using two‐tailed Student’s *t*‐test. *P < 0.05; **P < 0.01; and ***P < 0.001. Source data are available online for this figure.

Technologies to generate 3D cellular spheroids or various iPSC‐derived organoids have revolutionized our understanding of human cellular physiology and disease pathogenesis mechanisms, leading to accelerated therapeutic developments. Particularly, as disease model systems, generation of human 3D renal spheroids (Maliszewska‐Olejniczak *et al*, 2018; Secker *et al*, 2018) or stem cell‐derived kidney organoids (Taguchi & Nishinakamura, 2017; Forbes *et al*, 2018; Przepiorski *et al*, 2018; van den Berg *et al*,^2018^) have enabled more accurate predictions of native tissue‐level outcomes underlying complex pathophysiological processes. In a previous study, DesRochers *et al* (2015) reported that exposure of a 3D renal tissue model to Stx2a revealed a pathophysiological phenotype more consistent with changes seen *in vivo* compared to 2D renal cultures. Therefore, we verified our findings made using 2D cell cultures that pathogenic mechanisms in host cells exposed to Stxs are controlled through the regulation of O‐GlcNAcylation, using 3D renal culture systems or kidney organoids. Firstly, we examined cytotoxicity, renal injury biomarker expression, and inflammatory cytokine/chemokine secretion in Stx2a‐treated 3D‐human proximal tubular epithelial (HRPTEpi) spheroids or mini‐kidney spheroids comprising human primary proximal tubular epithelial cells with supporting cells including mesangial cells and glomerular endothelial cells in the presence or absence of OSMI‐1. Using microscopic analyses of spheroid collapse and measurement of spheroid diameter, Stx2a caused severe damage in the formation of 3D spheroids, whereas 3D structures were well‐maintained under conditions of co‐treatment with the OGT inhibitor OSMI‐1 in HRPTEpi spheroids (Fig 5A and B) and mini‐kidney spheroids (Fig 5D and E). In addition, O‐GlcNAc inhibition downregulated soluble pro‐inflammatory chemokine production upon Stx2a exposure, analyzed using supernatants collected from spheroid cultures (Fig 5C and F). Finally, we employed antibody array to examine the protective effect of OSMI‐1 on apoptosis in Stx2a‐treated spheroids from 3D culture of human mini‐kidneys. In the average fluorescence intensities associated with each marker protein, relative expressions of all pro‐apoptotic factors included in this array such as Bax, Bim, Caspase‐3/8, and FAS were noticeably increased in 3D renal spheroids by Stx2a exposure, but few were induced in Stx2a + OSMI‐1 co‐treated spheroids (Fig 5G and H).

**Figure 6:**
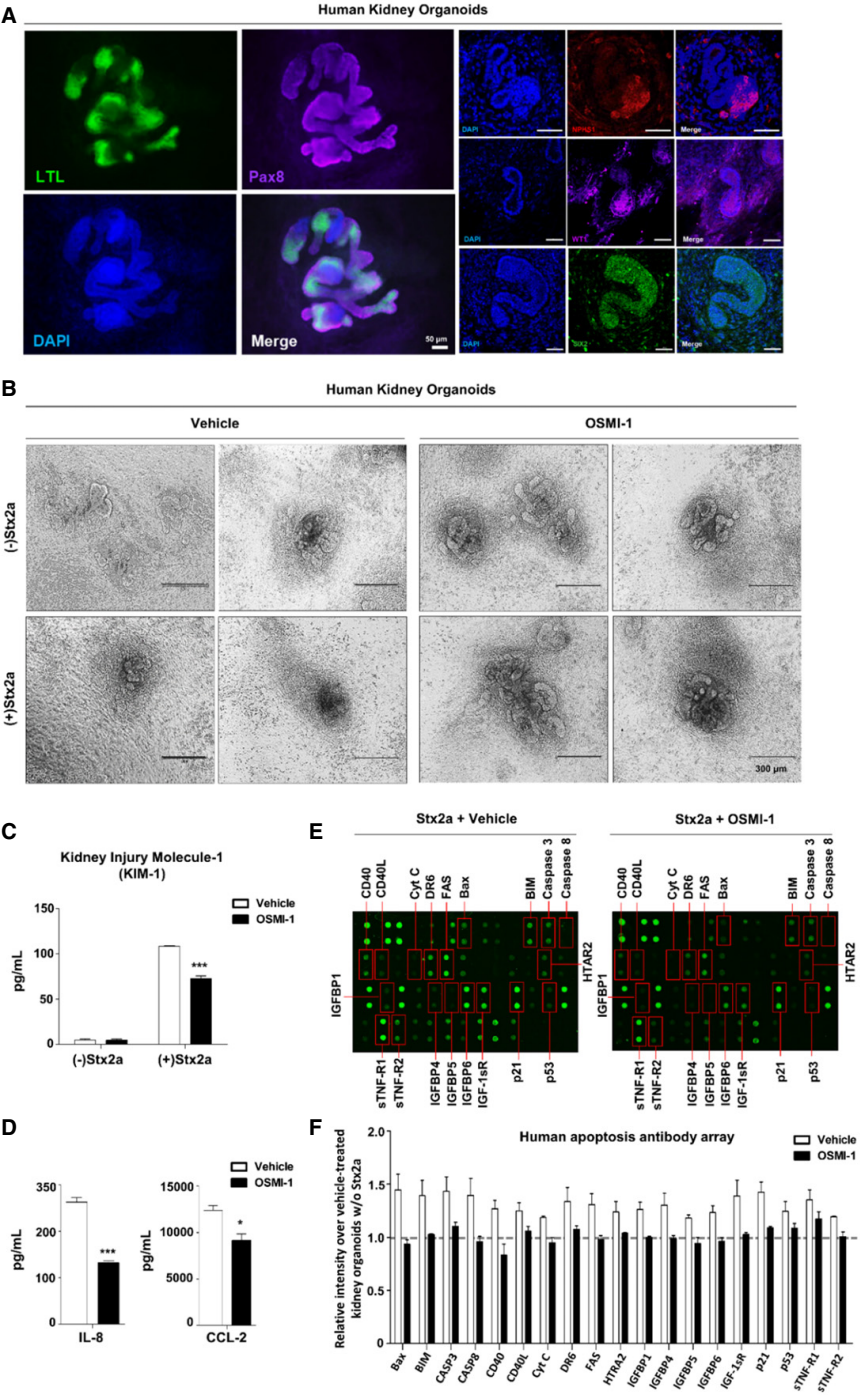
Downregulation of O‐GlcNAcylation protects iPSC‐derived human kidney organoids from injury caused by Stx2a exposure ARepresentative immunofluorescence images of the nephron structures within kidney organoids derived from hiPSCs on day 18 of the differentiation; proximal tubules (LTL, green), parietal epithelial cells (PAX8, purple), kidney glomerular podocytes (NPHS1, red), and nephron progenitors (WT1, purple and SIX2, green). Cell nuclei were stained with DAPI. Scale bars: 50 µm.BRepresentative images showing morphological collapses in iPSC‐derived human kidney organoids following Stx2a (10 ng/ml) treatment for 72 h in the presence or absence OSMI‐1 (10 µM, final).C, DELISAs were used to analyze the inhibitory effect of OSMI‐1 (10 µM, final) on kidney injury molecule‐1 (KIM‐1) secretion (C) and IL‐8 and CCL‐2 production (D) in culture supernatants of iPSC‐derived human kidney organoids exposed to Stx2a (10 ng/ml) for 72 h (*n* = 3 biological replicates). The effects of OSMI‐1 were compared with those of the vehicle (DMSO) controls.E, FHuman apoptosis antibody array analysis of multiple proteins using pooled lysates from iPSC‐derived human kidney organoids following Stx2a (10 ng/ml) treatment for 72 h in the presence or absence of OSMI‐1 (10 µM, final). (E) Antibody spots representing signal differences are indicated in red boxes. (F) The graph shows the average of relative spot intensities for each protein compared to those measured in the control in the absence of Stx2a exposure (*n* = 2 biological replicates). Dashed line represents the reference point of the fold change. Raw values of fluorescence intensities are provided in the Source Data. Data information: Error bars for bar graphs are presented as mean ± SEM. Statistical analysis was performed using two‐tailed Student’s *t*‐test. *P < 0.05; **P < 0.01; and ***P < 0.001. Source data are available online for this figure.

In order to further mimic conditions *in vivo*, we generated human‐induced pluripotent stem cell (hiPSC)‐derived kidney organoids differentiated by a stepwise manner to result in the composition of renal tubular structures with typical nephron‐like morphology validated by marker expression of glomerular podocytes (Nephrosis 1, NPHS1; Wilms' tumor 1, WT1), glomerular parietal epithelial cells (paired box gene 8, PAX8), renal proximal tubule (lotus tetragonolobus lectin, LTL), and nephron progenitor cells (SIX Homeobox 2, SIX2) (Fig 6A). Most importantly, LTL marker expression implied that the kidney organoids consisted of tightly packed renal proximal tubules, well‐established targets of Stx2a‐induced apoptosis. To utilize the full potential of human kidney organoids as a convenient model, the organoids were also treated with the pharmacological inhibitor OSMI‐1 prior to intoxication with Stx2a. The kidney organoids exposed to Stx2a alone displayed a clearly different morphology compared to the vehicle‐only control, with no distinct nephron‐like segmentation. In contrast, morphological changes were not detected in OSMI‐1‐treated organoids followed by intoxication with Stx2a (Fig 6B). In order to monitor *in vivo*‐like kidney damage, secretion of kidney injury molecule‐1 (KIM‐1) as an indicator of acute renal failure was significantly increased in the supernatants from kidney organoids exposed to Stx2a compared to supernatants from intoxicated organoids pre‐treated with OSMI‐1 (Fig 6C), suggesting that O‐GlcNAc inhibition effectively reduces Stx2a‐induced renal injury. In addition, the secreted levels of chemokines IL‐8 and CCL‐2 were significantly decreased in the OSMI‐1 pre‐treated human kidney organoids compared to Stx2a treatment alone (Fig 6D). Finally, we employed antibody array to verify the protective effect of OSMI‐1 on apoptosis in the Stx2a‐treated human kidney organoids. Similar to the results in 3D renal spheroids, expression of several pro‐apoptotic factors, including Bax, Bim, Caspase‐3/8, and p21, was induced in human kidney organoids by Stx2a exposure, but the expression was not affected in OSMI‐1 pre‐treated organoids (Fig 6E and F). These data first demonstrate that human kidney organoids can be injured with Stx2a, and further, this renal damage is markedly rescued by blocking Stx‐induced O‐GlcNAcylation levels in host cells. Given that 3D cell culture systems and organoids are more advantageous in evaluating clinical drug responses by providing conditions similar to the *in vivo* environment than those using 2D culture systems, our results clearly suggest that O‐GlcNAcylation inhibitors may have sufficient potential to warrant additional study as therapeutic agents for prevention of extraintestinal complications that may follow infection with Stx‐producing bacteria.

### Administration of an OGT inhibitor considerably improves survival of mice challenged with a lethal dose of Stx2a

**Figure 7:**
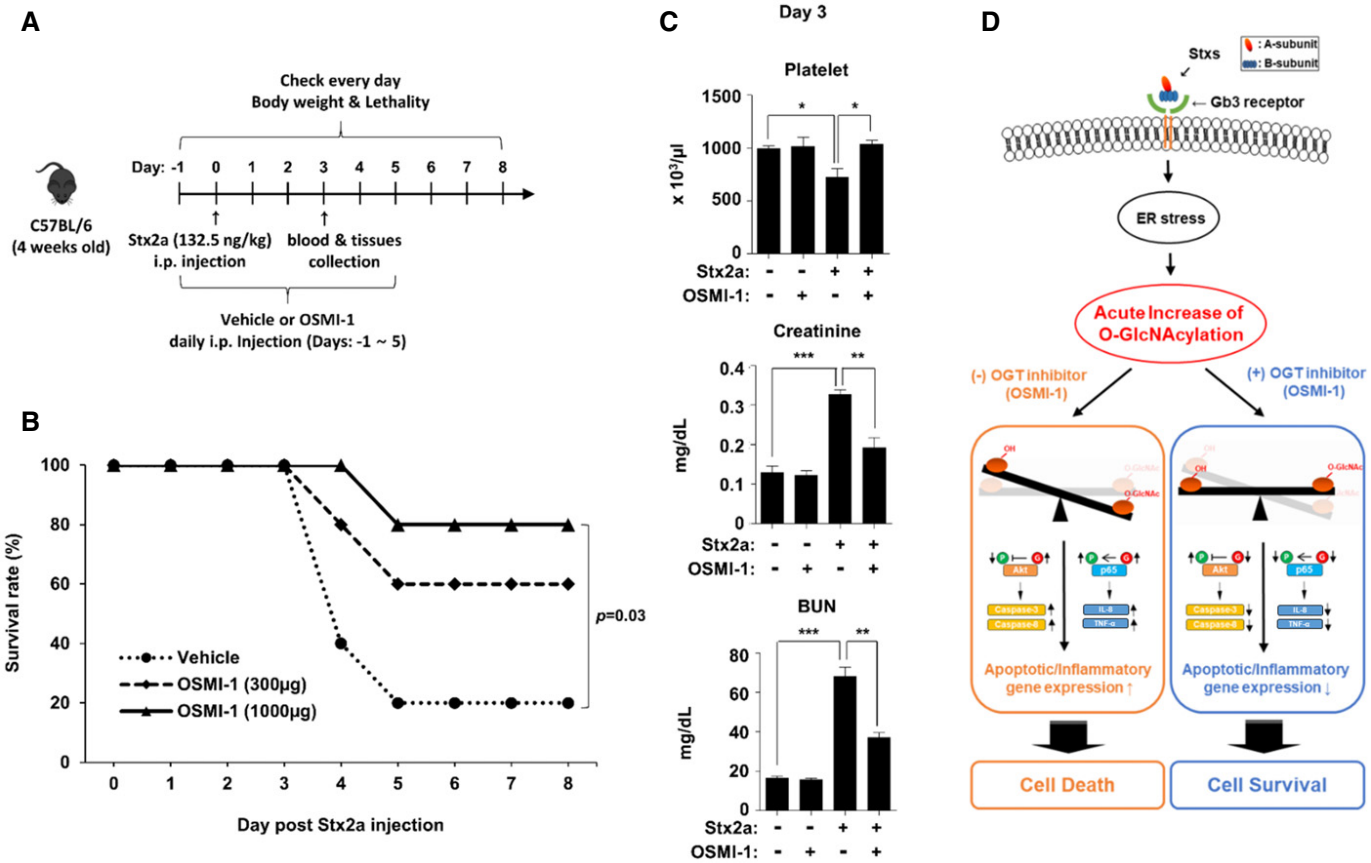
Treatment with an OGT inhibitor improves survival and clinical symptoms of mice challenged with Stx2a Timeline for injection of mice with Stx2a and OSMI‐1. Mice were pretreated with OSMI‐1 or vehicle alone beginning 1 day before injection of Stx2a. Body weight changes and mortality were monitored every day for 1 week to determine the survival rate. Blood and tissue samples were collected at day 3 after Stx2a challenge.Survival rate of mice challenged once with Stx2a (132.5 ng/kg) and injected daily with two doses (300 or 1,000 µg/mouse) of OSMI‐1 (*n* = 10, 5 per dose) or vehicle (*n* = 10). The effect of OSMI‐1 (1,000 µg/mouse) was compared with that of the vehicle control (a log‐rank test with GraphPad Prism software).Chemical and hematological analyses of blood pooled from three mice at day 3 after Stx2a challenge. Data are presented as mean ± SEM (*n* = 3 biological replicates from nine mice, two‐tailed Student’s *t*‐test). The effects of OSMI‐1 (1,000 µg/mouse) were compared with those of the vehicle control. *P < 0.05; **P < 0.01; and ***P < 0.001.Schematic illustration of proposed protective mechanism from Stx‐induced apoptotic and inflammatory responses through inhibition of O‐GlcNAcylation. Source data are available online for this figure.

**Figure EV5:**
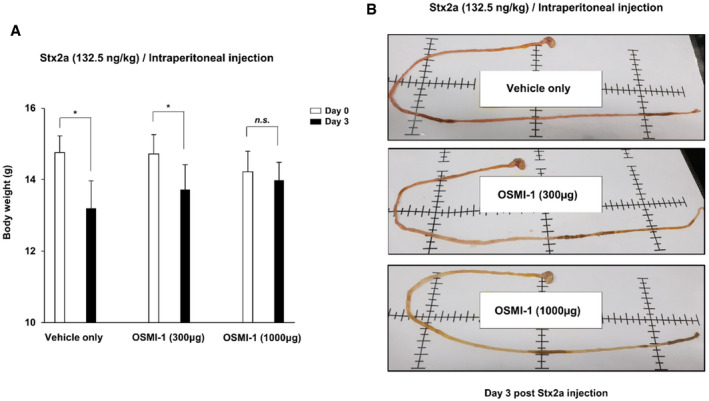
Treatment with an OGT inhibitor improves severe loss of body weight due to the hemorrhagic symptoms in the intestines of mice challenged with Stx2a Body weight changes in mice challenged with Stx2a in the presence or absence of OSMI‐1 (300 or 1,000 µg/mouse). Data are presented as mean ± SD (*n* = 5 biological replicates per group, two‐tailed Student’s *t*‐test). Comparisons between days 0 and 3 were indicated for each group. *P < 0.05; **P < 0.01; and ***P < 0.001.Representative images of the intestines of mice at day 3 after Stx2a injection and combinatorial treatment with two doses of OSMI‐1 or the vehicle alone. Source data are available online for this figure.

*In vitro* assays demonstrated that the OGT inhibitor OSMI‐1 suppressed Stx‐induced apoptosis and inflammatory responses in cells; therefore, we hypothesized that it would protect mice injected with a lethal dose of Stx2a. To establish the mouse model, we determined the concentration of purified Stx2a that induced an appropriate level of lethality upon intraperitoneal injection (Keepers *et al*, 2006; Cheng *et al*, 2013) and then treated with OSMI‐1 at doses previously established to be effective *in vivo* (Lee *et al*, 2020) to test its protective effect (Fig 7A). The survival rate of C57Bl/6 mice was ˜20% at 5 days after the injection of 132.5 ng/kg Stx2a, but OSMI‐1 apparently improved the survival rate by 60–80% in a dose‐dependent manner (Fig 7B). The most prominent clinical features of humans intoxicated with Stxs are severe kidney damage, thrombocytopenia, and hemorrhagic enteritis. In our mouse model, the amounts of blood urea nitrogen (BUN) and creatinine, which are renal toxicity markers, detected in the blood were significantly increased upon treatment with Stx2a, but restored to almost normal levels upon co‐treatment with OGT inhibitor OSMI‐1 (Fig 7C). OSMI‐1 also completely protected against the thrombocytopenia (reduction in the platelet count) in mice challenged with Stx2a (Fig 7C). In addition, the severe loss of body weight or the red coloration of the intestines due to hemorrhagic inflammatory responses induced by Stx2a administration was completely absent in mice receiving the OGT inhibitor (Fig EV5A and B). In summary, we demonstrated that O‐GlcNAc inhibition improves mortality and various disease symptoms caused by Stx2a exposure in mice, further reinforcing O‐GlcNAc inhibition as a novel strategy for the development of new agents to treat life‐threatening complications which may occur following infection with Stx‐producing bacteria.

## Discussion

Stxs are multi‐functional proteins that induce protein synthesis inhibition, ribotoxic and ER stress responses, apoptosis, autophagy, and inflammatory cytokine and chemokine production by activating various host cellular signaling pathways (Lee *et al*, 2016a). While the capacity of Stxs to affect the functions of protein kinases and transcriptional activators has been explored (Parello *et al*, 2015), there are few studies assessing the ability of Stxs to influence other PTM. To our knowledge, this is the first report to directly assess the extent of O‐GlcNAcylation, a type of PTM, under conditions of cell stress induced by Stx intoxication *in vitro* and *in vivo*. The major conclusions of this study as described in Fig 7D are that an acute increase in O‐GlcNAcylation induced by Stx1a and Stx2a is detrimental to host cells because it triggers ER stress‐mediated pro‐apoptotic and pro‐inflammatory responses via regulation of the serine/threonine kinase Akt and NF‐κB component p65. The cleavage of pro‐caspase‐3 and the detection of TUNEL‐positive cells were directly correlated with the kinetics of increased O‐GlcNAcylation. Treatment of THP‐1 cells with Stx2a resulted in increased expression of cytokines TNF‐α, IL‐1β, and IL‐6, and chemokines IL‐8, MIP‐1α, and CCL‐2. Apoptosis induction and cytokine/chemokine expression induced by Stx2a intoxication were suppressed by co‐treatment of cells with a pharmacological inhibitor of O‐GlcNAcylation or by transfection with a small interfering RNA directed against O‐linked *N*‐acetylglucosamine transferase. Using lectin pull‐down assays, we showed that both Akt and p65 are O‐GlcNAcylated following Stx2a intoxication. Protective effects of O‐GlcNAcylation inhibition were manifest in Stx2a‐treated 3D primary renal epithelial cell spheroids or kidney organoids.

Our previous study showed that Stx1a signals through the PI3K/Akt/mTOR pathway in THP‐1 cells via a mechanism that does not require toxin enzymatic activity. Signaling through this pathway mediated a transient increase in cytokine expression via hyperphosphorylation of eIF4E‐binding proteins, but Akt negatively regulated production of pro‐inflammatory cytokines by inactivating the positive regulatory factor glycogen synthase kinase 3α/β (Cherla *et al*, 2009). In contrast to these earlier studies, we show here that O‐GlcNAcylation of target proteins requires toxin enzymatic activity as a mutated holotoxin lacking enzymatic activity (Stx2a^mut^) failed to trigger rapid O‐GlcNAcylation. Taken together, these findings highlight the fact that toxin A and B subunits may independently trigger the activation of different signaling pathways at different points during the retrograde transport process of toxins from the cell membrane to the ER. Finally, several lines of evidence suggest that the abnormal Stx‐induced increase in O‐GlcNAcylation is associated with apoptosis and inflammation *in vivo*. We showed that the survival rate and renal, hematological, and intestinal symptoms of mice administered a lethal dose of Stx2a were improved by pharmacological inhibition of O‐GlcNAcylation using OGT inhibitor OSMI‐1, indicating that an acute increase in O‐GlcNAcylation in HUS patients infected with STEC may be a critical factor contributing to disease progression. These results suggest a rationale for the development of therapeutic agents for Stx‐related disorders based on the inhibition of aberrant O‐GlcNAcylation induced by the toxins. Because cells are equipped with multiple layers of control mechanisms acting at the transcriptional, post‐transcriptional, and post‐translational levels to maintain cellular O‐GlcNAc levels within the normal range (Kazemi *et al*, 2010; Zhang *et al*, 2014; Park *et al*, 2017b; Yang & Qian, 2017; Qian *et al*, 2018), a short‐term treatment regimen using therapeutic agents directed against O‐GlcNAcylation‐related acute symptoms that may follow infection by Stx‐producing bacteria represents an attractive strategy that minimizes concerns about adverse effects caused by artificial manipulation of O‐GlcNAc homeostasis.

In the present study, we identified proteins, including Akt and p65, whose activities are directly influenced by the level of O‐GlcNAcylation in Stx2a‐intoxicated cells. However, alteration of O‐GlcNAcylation induced by Stx exposure may globally affect the functions of a much greater variety of target proteins in addition to Akt and p65 (Hart *et al*, 2011; Bond & Hanover, 2013). Besides, these target proteins are often linked to molecular mechanisms that elicit opposite effects (Hanover *et al*, 2005; Forsythe *et al*, 2006; Hart *et al*, 2011). Indeed, an increase in O‐GlcNAcylation can be both a positive and negative regulator of inflammatory responses. For example, hyper‐O‐GlcNAcylation promotes the pro‐inflammatory response by directly activating various proteins involved in the NF‐κB pathway, such as c‐Rel and p65 (Ryu & Do, 2011; Allison *et al*, 2012; Ramakrishnan *et al*, 2013). On the contrary, hyper‐O‐GlcNAcylation in macrophages was reported to promote the anti‐inflammatory response (Hwang *et al*, 2019; Li *et al*, 2019). Of particular interest, c‐Rel activated by O‐GlcNAcylation participates in both pro‐ and anti‐inflammatory responses (Hwang *et al*, 2017). Upregulation of cellular O‐GlcNAcylation is often linked with apoptosis, as observed in this study, in several cell types including pancreatic β‐cells, myocardial cells, and cancer cell as well as neuronal cells in the brain (Liu *et al*, 2004, 2011; Kang *et al*, 2008; Rajamani & Essop, 2010). However, according to some reports, hyper‐O‐GlcNAcylation is also able to enhance survival signals such as the anti‐apoptotic Bcl‐2 pathway (Champattanachai *et al*, 2008). Therefore, it seems that changes in cellular functions associated with O‐GlcNAcylation may depend on which target proteins react more quickly and sensitively to alterations in O‐GlcNAcylation. In other words, cells may behave differently under the same conditions depending on how the balance in expression or activity of target proteins is tilted.

In summary, we demonstrated that O‐GlcNAcylation is rapidly increased in Stx‐exposed cells, and this PTM was directly linked with induction of apoptosis and pro‐inflammatory responses caused by Stxs. We confirmed that reduction in O‐GlcNAcylation alleviated the effects of Stx intoxication. Treatment of intoxicated cells with an OGT inhibitor or transfection with siRNA directed against OGT adversely affected the pathogenic activities of Stx. In addition, except for our present study, there has been no approach to use human iPSC‐derived kidney organoids or mini‐kidney spheroids as human kidney surrogate models for studying bacterial toxin or Stx2a‐mediated pathogenesis. Consistent with our findings using 2D cell cultures of THP‐1 and human renal proximal tubule epithelial cells, we demonstrated that human kidney organoids may be intoxicated with Stx2a at 10 ng/ml dose resulting in the activation of pro‐inflammatory and apoptotic cascades. Furthermore, the injury of 3D human kidney organoids can be mitigated by inhibiting O‐GlcNAcylation levels (Fig 6). Moreover, treatment with an optimal dose of a pharmacological inhibitor of O‐GlcNAcylation in a mouse model revealed the potential to ameliorate Stx‐associated acute renal failure and thrombocytopenia, which may follow the bloody diarrhea caused by Stx‐producing bacteria. As such, our findings that the O‐GlcNAc inhibitory strategy showed a therapeutic effect on the Stx‐intoxicated human renal organoid, as well as on the mouse model, is an encouraging result for the development of treatment agents. However, in order to enhance the medical impact of these findings, studies on how to translate this inhibitory effect into treatment in humans, including the safety evaluation of currently developed or to‐be‐developed O‐GlcNAc inhibitors, will be necessary for clinical applications to prevent or interrupt the Stx‐mediated HUS disease progression in patients. In addition, although there have been no reports describing the involvement of EHEC‐produced effector molecules delivered via the type 3 secretion system for regulating O‐GlcNAcylation in the infected mammalian host cells, the increased knowledge of the O‐GlcNAcylation‐mediated positive or negative pathways regulated by bacterial effector molecules translocated into the infected host may be a future experimental approach to provide additional promising therapeutic targets to prevent HUS disease progression. Furthermore, according to our unpublished data (K.‐S. Lee, M.‐S, Lee), once THP‐1 cells were exposed to tunicamycin instead of Stx to induce ER stress, O‐GlcNAc levels were increased again, similar to Stx exposure, and treatment with O‐GlcNAc inhibitor could reduce the tunicamycin‐mediated LDH release and cytokine production. Although these results suggest the possibility that O‐GlcNAc regulation may be widely applied to various diseases induced by ER stress, further study is needed to verify this hypothesis. In particular, since Stx‐mediated ER stress is cell‐type specific, it will need to be validated in other cell systems than Stx‐sensitive THP‐1 cells.

## Materials and Methods

### Toxins and reagents

Purified Stx2a and Stx2a^mut^, which harbors the triple mutation Y77S/E167Q/R170L in the enzymatic active site of the A‐subunit, were purchased from NIAID (NIH Biodefense and Emerging Infections Research Repository; BEI Resources, Manassas, VA, USA). Stx1a was purified from recombinant Stx1a‐expressing *E. coli* DH5α (pCKS112) by sequential ion exchange and immunoaffinity chromatography. The purity of Stx1a was determined by sodium dodecyl sulfate‐polyacrylamide gel electrophoresis (SDS‐PAGE), silver staining, and western blot analysis. The level of endotoxin contaminants was reduced to < 0.1 ng/ml using ActiClean Etox columns (Sterogene Bioseparations, Carlsbad, CA, USA). The degree of endotoxin contamination was assessed by a *Limulus* Amoebocyte lysate assay (Associates of Cape Cod, East Falmouth, MA, USA). OSMI‐1 was initially purchased from Sigma (SML 1621) and subsequently from Cayman (21894), and Retro‐2 was from Sigma (SML 1085). MKC‐3946 (HY‐19710) and Thiamet G (HY‐12588) were purchased from MedChemExpress.

### Cell culture

The human monocytic cell line THP‐1 was purchased from the American Type Culture Collection (Manassas, VA, USA). Cells were grown in RPMI 1640 medium (ThermoFisher Scientific, Waltham, MA, USA) containing 10% fetal bovine serum (FBS; HyClone, Logan, UT, USA), 5.0 µg/ml streptomycin, and 5 U/ml penicillin at 37°C in a humidified incubator containing 5% CO_2_. THP‐1 cells (2.0 × 10^6^ cells/well) were seeded into six‐well plates and differentiated into D‐THP‐1 cells by treatment with 50 ng/ml phorbol‐12‐myristate 13‐acetate (PMA; Sigma‐Aldrich, St. Louis, MO, USA) for 48 h. Thereafter, cells were washed three times with cold sterile Dulbecco’s phosphate‐buffered saline (DPBS; Sigma‐Aldrich) and incubated in fresh medium lacking PMA and containing 10% FBS, 5.0 µg/ml streptomycin, and 5 U/ml penicillin at 37°C in an incubator containing 5% CO_2_. After changing the medium every 24 h for the next 3 days, cells were starved for 16 h in RPMI 1640 medium containing 0.5% FBS to reduce background kinase activity stimulated by PMA and then experiments were performed.

Primary human proximal tubule epithelial (HRPTEpi) cells were purchased from Cell Applications, Inc. (San Diego, CA, USA) and cultured in 5% CO_2_ at 37°C in Human RenaEpi Growth Medium (Cell Applications, Inc.) containing 100 U/ml penicillin and 100 μg/ml streptomycin. HRPTEpi cells were seeded into six‐well plates, washed twice with 1× DPBS, and treated with 0.1 or 1.0 ng/ml Stx2 for 24, 48, or 72 h in Human RenaEpi Basal Medium (Cell Applications, Inc.) containing 5% heat‐inactivated FBS (GE Healthcare Life Sciences, Queensland, Australia).

### 3D spheroids culture

The ready‐to‐use 3D human renal proximal tubular epithelial spheroids kit (SP3D‐4100) and 3D human mini‐kidney spheroids kit (SP3D‐4108) were purchased from ScienCell Research Laboratories (Carlsbad, CA, USA) and cultured according to the manufacturer’s instructions. Briefly, each frozen vial containing ≥ 4.0 × 10^3^ spheroids was thawed and re‐suspended in 3D Epithelial Spheroid Medium containing 2% FBS or 3D Kidney Spheroid Medium containing 5% FBS, and to each media, supplements included in the kit were added. After re‐suspension, approximately 167 spheroids were seeded in a 24‐well typed Ultra‐Low Binding Culture Plate provided in the kit and incubated for 24 h. After incubation, 70% of culture medium was changed with fresh medium and incubated for 3 days. After 3 days, 70% of culture medium was changed with fresh medium and incubated for 24 h. After 24 h, 10 µM of OSMI‐1 or the same volume of DMSO was added to each well and incubated for 12 h. After incubation, spheroids were treated with 10 ng/ml of Stx2a for 72 h. The resultant phenotypes were captured using EVOS M5000 fluorescence microscope (ThermoFisher Scientific, Waltham, MA, USA).

### Western blot analysis

Cells were harvested and lysed in CETi Lysis Buffer containing protease and phosphatase inhibitors (TransLab, Daejeon, Korea). Protein concentrations were determined using a Pierce BCA Protein Assay Kit (ThermoFisher Scientific, Waltham, MA, USA), and 30–50 µg of proteins was loaded into each lane of a 4–12% Bis–Tris SDS‐PAGE protein gel (Invitrogen Corp., Carlsbad, CA, USA) and electrophoresed at 200 V. Thereafter, proteins in the gel were transferred onto a polyvinylidene difluoride membrane. The membrane was blocked for 1 h with 5% non‐fat milk prepared with TBST (20 mM Tris [pH 7.6], 137 mM NaCl, and 0.05% Tween 20) and then washed three times (5 min each) with TBST. Thereafter, the membrane was probed with a primary antibody overnight at 4°C, washed three times as previously described, and labeled with a horseradish peroxidase (HRP)‐conjugated secondary antibody for 90 min at room temperature in the dark. Bands were detected using an Odyssey Scanner (LI‐COR, Bad Homburg, Germany). The following antibodies were used: mouse monoclonal antibodies specific for O‐GlcNAc (RL2; ThermoFisher Scientific, MA1‐072 or CTD110.6; Santa Cruz Biotechnologies, sc‐59623), XBP1s and GFAT1 (Santa Cruz Biotechnologies), and p65 (Cell Signaling Technology, Danvers, MA, USA); rabbit monoclonal antibodies specific for human OGT (Sigma), phospho‐IRE1α (abcam), PERK, phospho‐PERK, Grp78, IRE1α, phospho‐p65, caspase‐3, Akt, phospho‐Akt, Bad, and phospho‐Bad (Cell Signaling Technology); and a HRP‐conjugated monoclonal antibody specific for human β‐actin and secondary antibodies specific for mouse/rabbit IgG (Cell signaling Technology) or mouse IgM (abcam).

### TUNEL assay, flow cytometry, and fluorescence microscopy

The TUNEL assay to detect apoptotic cells was performed using an APO‐DIRECT™ Kit (TONBO Bioscience, San Diego, CA, USA) following the manufacturer’s protocol. Briefly, cells (1 × 10^6^ cells/ml) were fixed in 1% (w/v) paraformaldehyde prepared in phosphate‐buffered saline (PBS, pH 7.4) for 1 h and then permeabilized in 70% ethanol for 30 min on ice. After several washes using Wash Buffer provided with the kit to completely remove ethanol, cell pellets were re‐suspended in 50 µl of DNA Labeling Solution [0.5 µl of TdT enzyme, 8 µl of fluorescein isothiocyanate (FITC)‐conjugated dUTP, and 10 µl of 5× Reaction Buffer in 31.5 µl of water] for 1 h at 37°C and then washed twice with Rinse Buffer provided with the kit. Finally, cells were incubated in propidium iodide (PI)/RNase solution for 30 min at room temperature and then analyzed using a GALLIOS flow cytometer (Beckman Coulter, Miami, FL, USA) with FlowJo software. For fluorescence microscopy, cells were adhered to a six‐well plate pre‐coated with poly‐L‐lysine solution (Sigma‐Aldrich) after DNA labeling without PI staining, and PBS was added to the plate. Apoptotic cells possessing DNA breaks labeled with FITC‐dUTP by TdT were imaged using an inverted microscope (IX71; Olympus, Tokyo, Japan).

### Cell viability assay

The WST‐1 dye‐based assay was performed using EZ‐CYTOX (Daeil Lab Service, EZ‐1000) following the manufacturer’s protocol. Briefly, 100 µl of cultured THP‐1 cells treated with Stx2a only or Stx2a plus OSMI‐1 were transferred to a 96‐well plate in triplicate and incubated with 10 µl of WST‐1 reagent for 2 h under standard culture conditions as described above. Thereafter, absorbance at 450 nm was measured using a SpectraMAX 190 Microplate Reader (Molecular Devices, Menlo Park, CA, USA). Cytotoxicity was assessed using a Pierce™ LDH Cytotoxicity Assay Kit (ThermoFisher Scientific, 88953). THP‐1 cells were transfected with siOGT or Akt (WT or T305A/T312A), and then treated with Stx2a. Culture medium was harvested after incubation for an appropriate duration, and culture medium lacking cells was used as a negative control. Dead or dying cells release cytosolic enzymes including lactate dehydrogenase (LDH) into the culture medium. The enzymatic response of LDH to the Reaction Mixture provided with the kit leads to production of red formazan, which can be measured using a spectrophotometer (SpectraMAX 190 Microplate Reader, Molecular Devices).

### RT‐qPCR

RNA was extracted using NucleoSpin RNA Plus (Macherey‐Nagel, Düren, Germany) following the manufacturer’s instructions. cDNA synthesis with reverse transcriptase and cDNA amplification were performed using a RT‐qPCR kit (NanoHelix Co. Ltd., Daejeon, Korea). Real‐time PCR was performed using the LightCycler^®^ 96 System (Roche, Mannheim, Germany). The cycling conditions for real‐time PCR were as follows: a common amplification step with an initial cycle for cDNA synthesis at 50°C for 30 min, followed by heating at 95°C for 15 min to activate the DNA polymerase, and then 40 cycles of denaturation at 95°C for 20 s and annealing and extension at 60°C for 60 s. SYBR Green Technology was used to quantify the results. Expression levels were normalized against that of GAPDH. Primer sequences are listed in Table 1.

**Table 1:** Sequences of the primers used for RT‐qPCR.

Gene	Type	Sequence (5′→3′)
IL‐8	Forward	AGC TGA TGG CCC TAA ACA GA
	Reverse	CAT CCA GAG GGC AGA GGT C
MIP‐1α	Forward	GCC TTC CAG TCA CTT GGT CT
	Reverse	ATG TTC CCA AGG AGC TCA GA
TNF‐α	Forward	AGC CCA TGT TGT AGC AAA CC
	Reverse	TCT CAG CTC CAC GCC ATT
IL‐1β	Forward	GCT GAG GAA GAT GCT GGT TG
	Reverse	GAA GGG AAA GAA GGT GCT CA
IL‐6	Forward	TTC TCC ACA AGC GCC TTC
	Reverse	GGA ATC TTC TCC TGG GGG TA
GAPDH	Forward	GCA CCG TCA AGG CTG AGA AC
	Reverse	TGG TGA AGA CGC CAG TGG A

### ELISAs

The levels of human IL‐6, IL‐1β, IL‐8, CCL‐2, CCL‐3, and TNF‐α in culture media were measured using ELISA kits (Invitrogen Corp.) following the manufacturer’s instructions. In brief, half‐area microplates (Corning, Corning, NY, USA) were coated with 50 µl of each capture antibody solution and incubated overnight at 4°C. Wells were washed three times (1 min each) with PBST (10 mM phosphate buffer, 2.7 mM KCl, 137 mM NaCl, and 0.05% Tween 20, [pH 7.4]) and blocked for 1 h at 37°C with 100 µl of 1× ELISA Diluent. Next, wells were washed three times and incubated with 50 µl of each sample for 2 h at room temperature. Thereafter, wells were washed as described above and incubated with 50 µl of each detection antibody solution for 1 h at room temperature, washed again, and incubated with 50 µl of streptavidin‐HRP for 30 min at room temperature in the dark. Finally, wells were washed five times and incubated with 50 µl of substrate solution for 15 min at room temperature in the dark. After color development, 25 µl of 2 M H_2_SO_4_ was added to stop the reaction, and absorbance at 450 nm was measured using a SpectraMax 190 Microplate Reader (Molecular Devices).

### Transfection and knockdown

The human AKT present on pCMV3‐C‐FLAG was purchased from Sino Biological (Beijing, China). Threonine at 305 and 312 of AKT was replaced to alanine using a commercial gene synthesis service (Bioneer, Daejeon, Korea). For overexpressing of Akt, THP‐1 cells (5.0 × 10^5^ cells/well) were seeded into six‐well plates in RPMI 1640 (Corning) containing 5% FBS (HyClone) at 37°C in a humidified incubator containing 5% CO_2_ at 24 h before transfection. Thereafter, 250 µl of Opti‐MEM I Reduced Serum Medium (Gibco‐Invitrogen, Waltham, MA, USA) was mixed with 500 ng of plasmid and 1.5 µl of TransIT‐X2 and incubated at room temperature for 30 min. The TransIT‐X2:plasmid complexes were added to each well and incubated for 24 h, followed by exposure to Stx2a for 9 h.

siRNA for OGT (siOGT) and scrambled siRNA (siScr) (ThermoFisher Scientific, Waltham, MA, USA) were transfected using TransIT‐X2 (Mirus Bio, Madison, WI, USA) following the manufacturer’s manual. In brief, THP‐1 cells (5.0 × 10^5^ cells/well) were seeded into 12‐well plates in Dulbecco’s Modified Eagle’s Medium (Corning) containing 10% FBS (HyClone), 5.0 µg/ml streptomycin, and 5 U/ml penicillin at 37°C in a humidified incubator containing 5% CO_2_ at 24 h before transfection. Thereafter, 250 µl of Opti‐MEM Ⅰ Reduced‐Serum Medium (Gibco‐Invitrogen, Waltham, MA, USA) was mixed with 6.8 µl of 10 µM siScr or siOGT in sterile tubes (final concentration 25 nM). Next, 7.5 µl of TransIT‐X2 was added to each tube and incubated at room temperature for 30 min. The TransIT‐X2:siRNA complexes were added to each well and incubated for 24 h. Finally, cells were washed with cold sterile DPBS and re‐suspended in fresh RPMI 1640 medium containing 5% FBS. All experiments were performed after re‐suspension of cells.

### High‐throughput RNA sequencing

#### RNA isolation

Total RNA from the Stx2a‐treated HRPTEpi cells in the presence or absence of OSMI‐1 was isolated using Trizol reagent (Invitrogen Corp., Carlsbad, CA, USA). RNA quality was assessed by Agilent 2100 bioanalyzer (Agilent Technologies, Amstelveen, The Netherlands), and RNA quantification was performed using ND‐2000 Spectrophotometer (ThermoFisher Scientific, Waltham, MA, USA).

#### Library preparation and sequencing

Libraries were prepared from total RNA using the NEBNext Ultra II Directional RNA‐Seq Kit (NEW ENGLAND BioLabs, Inc., UK). The isolation of mRNA was performed using the Poly(A) RNA Selection Kit (LEXOGEN, Inc., Austria). The isolated mRNAs were used for the cDNA synthesis and shearing, following the manufacturer’s instructions. Indexing was performed using the Illumina indexes 1––12. The enrichment step was carried out using PCR. Subsequently, libraries were checked using the Agilent 2100 bioanalyzer (DNA High Sensitivity Kit) to evaluate the mean fragment size. Quantification was performed using the library quantification kit with a StepOne Real‐Time PCR System (Life Technologies, Inc., Carlsbad, CA, USA). High‐throughput sequencing was performed as paired‐end 100 sequencing using NovaSeq 6000 (Illumina, Inc., San Diego, CA, USA).

#### Data analysis

A quality control of raw sequencing data was performed using FastQC (http://www.bioinformatics.babraham.ac.uk/projects/fastqc/). Adapter and low‐quality reads (< Q20) were removed using FASTX_Trimmer (http://hannonlab.cshl.edu/fastx_toolkit/) and BBMap (https://sourceforge.net/projects/bbmap/). Then, the trimmed reads were mapped to the UCSC Human genome (hg19) using TopHat (Trapnell *et al*, 2009). Gene expression levels were estimated based on read count and Fragments Per kb per Million (FPKM) values calculated using Cufflinks (Roberts *et al*, 2011). The FPKM values were normalized based on quantile normalization method using EdgeR within R (https://www.r‐project.org/). Data mining and graphic visualization were performed using ExDEGA (Ebiogen Inc., Korea). To define differentially expressed genes (DEGs), adjusted |log2fold‐change (FC)| ≥ 1.5 and *P* < 0.05 were selected as the cut‐off values. The functions and associated pathways of the DEGs were further analyzed using the Gene Ontology (GO) and Kyoto Encyclopedia of Genes and Genomes (KEGG) pathway using the DAVID database (https://david.ncifcrf.gov/).

### Kidney organoid cell culture

The differentiation of hiPSCs (human‐induced pluripotent stem cells) (Jeon *et al*, 2018) into human kidney organoids was performed using STEMdiff™ Kidney Organoid Kit (StemCell Technologies, Vancouver, BC, Canada; 05160) following the manufacturer’s instructions. Briefly, hiPSCs (5.0 × 10^3^ cells/wells) were seeded in a black 96‐well Corning^®^ Matrigel^®^‐coated plate (Corning, Corning, NY, USA; 356231) in mTeSR™1 medium (StemCell Technologies, Vancouver, BC, Canada; 85850) supplemented with 5× mTeSR™1 supplement (StemCell Technologies, Vancouver, BC, Canada; 85850) and 10 µM of Y‐27632 (StemCell Technologies, Vancouver, BC, Canada; 72302) and incubated in a 5% CO_2_ incubator at 37°C. After 24 h, all media of the wells were replaced with 0.25 mg/ml of Matrigel^®^ and mTeSR™1 medium supplemented with 5× mTeSR™1 supplement without Y‐27632 to generate cavitated hPSCs spheroids. After 24 h, all media of the wells were replaced with 200 µl of STEMdiff™ kidney basal medium supplemented with 100× STEMdiff™ kidney supplement SG which was included in the kit to induce late primitive streak. After 36 h, all media were changed to STEMdiff™ kidney supplemented with STEMdiff™ kidney supplement DM (Stage 2 medium) in the kit. All media were changed with Stage 2 medium every 2–3 days during an 18‐day incubation period. Experiments were performed on the 18th day.

### Kidney organoid immunostaining

All kidney organoids were washed in 1× DPBS and fixed for 15 min at room temperature in 4% paraformaldehyde in 1× DPBS. After fixation, all wells were washed three times with 1× DPBS and incubated with 0.2% Triton™ X‐100 (Promega, Madison, WI, USA; PRH5141) in 1× DPBS for 15 min at room temperature to permeate kidney organoids. After permeabilization, 200 µl of 10% donkey serum (MilliporeSigma, St. Louis, MO, USA) in PBS‐T (blocking buffer) was added to each well and incubated for 30 min at room temperature to block wells. After blocking, each primary antibody diluted in blocking buffer (Table 2) was added and incubated at 4°C in a dark room for 12 h. After incubation, all wells were washed six times with DPBS for 5 min. After washing, each secondary antibody, 1 in 1,000 in blocking buffer, was added to each well and incubated at 4°C in a dark room for 12 h. After incubation, all wells were washed six times with DPBS for 5 min. After the washing step, all images were acquired using a confocal microscope (OLYMPUS IX83).

**Table 2:** Primary and secondary antibodies for immunostaining kidney organoids (Subramanian *et al*, 2019).

Primary antibody	Dilution	Catalog number	Secondary antibody	Catalog number
LTL	1:200	Vector Laboratories B‐13225	Alexa Fluor^®^ 488‐conjugated Streptavidin	Jackson ImmunoResearch 016‐540‐084
Pax8	1:300	VWR 10091‐826	Alexa Fluor^®^ 647‐conjugated Donkey Anti‐Rabbit IgG	Jackson ImmunoResearch 711‐605‐152i
NPHS1	1:300	R&D Systems AF4269‐SP	Alexa Fluor^®^ 594‐conjugated Donkey Anti‐Sheep IgG	Jackson ImmunoResearch 713‐585‐147
SIX2	1:100	Proteintech 11562‐1‐AP	Alexa Fluor^®^ 647‐conjugated Donkey Anti‐Rabbit IgG	Jackson ImmunoResearch 711‐605‐152i
WT1	1:200	Cell Signaling Technology 83535	Alexa Fluor^®^ 647‐conjugated Donkey Anti‐Rabbit IgG	Jackson ImmunoResearch 711‐605‐152i

### Human apoptosis antibody array

Lysates prepared from 3D human mini‐kidneys or iPSC‐derived human kidney organoids were analyzed with a semi‐quantitative Human Apoptosis Array (RayBiotech, Inc., Norcross, GA, USA; AAH‐APO‐G1‐4) that detects 40 proteins in one experiment. All the following processes were performed according to the manufacturer’s instructions. In brief, protein lysates were added to each array and incubated with a biotinylated antibody mixture that binds each target protein. After washing, Cy3‐conjugated streptavidin was added for fluorescence detection. The signals were detected using a laser scanner (GenePix 4100A, Molecular Devices, Menlo Park, CA, USA) and analyzed with GenePro7.0 software (Molecular Devices, Menlo Park, CA, USA).

### Mice and Stx2a challenge

All mice were kept under a specific pathogen‐free facility in the Korea Research Institute of Bioscience and Biotechnology (KRIBB, Daejeon, Korea), and all animal studies were performed in accordance with protocols approved by the Institutional Animal Care and Use Committee at KRIBB (approval number: KRIBB‐AEC‐19044). Mice were maintained under a 12‐h light–dark cycle at 23°C and fed a standard diet and water *ad libitum*. Four‐week‐old C57BL/6 mice were challenged with an intraperitoneal (IP) injection of Stx2a (132.5 ng/kg of body weight). In addition, 0.3 or 1.0 mg of OSMI‐1 (Cayman Chemical, Ann Arbor, MI, USA) was solubilized in water containing 4.5% DMSO (Thermo Fisher Scientific, Waltham, MA, USA) and 5% Tween 80 (MilliporeSigma, St. Louis, MO, USA) as described previously (Lee *et al*, 2020). Mice were administered 100 µl of OSMI‐1 solution daily by IP injection from 1 day prior to Stx2a treatment. Body weight changes and mortality were monitored every day for 1 week to determine the survival rate. Blood and tissue samples were collected from three mice per group at day 3 after Stx2a challenge. For clinical pathology examination, blood was drawn from the retro‐orbital sinus of each mouse and transferred to a EDTA MiniCollect tube (Greiner Bio‐One, Frickenhausen, Germany). All chemical and hematological analyses of blood were performed by K‐Bio (Osong Medical Innovation Foundation, Osong, Korea). BUN and creatinine levels were measured using Konelab PRIME 60i (ThermoFisher Scientific, Vantaa, Finland), and the platelet count was analyzed using ADVIA 2120i (Siemens Healthcare Diagnostics, Tarrytown, NY, USA) by a flow cytometry‐based method.

### Statistical analysis

All data are expressed as the mean ± SEM or SD Analyses were performed using GraphPad Prism version 5.00 (GraphPad Software, Inc., La Jolla, CA, USA) or Microsoft Excel. The Student’s *t*‐test (for paired or unpaired samples) was used for all statistical analyses, except for the comparison of mouse survival rate assessed using a log‐rank test. The animals/samples were randomly allocated into experimental groups. Detailed information on statistical tests and replicates is provided in each figure legend. *P*‐values < 0.05 were considered statistically significant (*P < 0.05; **P < 0.01; and ***P < 0.001).

## Author contributions

Designed the study, M‐SL and S‐KP; performed the experiments, K‐SL, JEL, PL, BCJ, MYS, SK, JL, J‐SK, D‐JK, and JHK; provided critical reagents and scientific insight, JL, J‐SK, D‐JK, JHK, and VLT; analyzed the data, K‐SL, JL, VLT, M‐SL, and S‐KP; and wrote the paper, VLT, M‐SL, and S‐KP.

## Conflict of interest

The authors declare that they have no conflict of interest.

## For more information

1. https://apps.who.int/iris/discover?query=STEC
2. https://oglcnac.org/search/

## Supporting information



Expanded View Figures PDF

Dataset EV1

Source Data for Expanded View

Source Data for Figure 1

Source Data for Figure 2

Source Data for Figure 3

Source Data for Figure 4

Source Data for Figure 5

Source Data for Figure 6

Source Data for Figure 7

## Data Availability

The raw data including uncropped western blots from the entire manuscript are included in Source data files. Any additional information/data underlying this study will be made available by the corresponding author upon request. The datasets produced in this study are available in the following databases: RNA‐Seq data: Sequence Read Archive PRJNA778611 (https://www.ncbi.nlm.nih.gov/sra/?term=PRJNA778611).
