# Supplementary material for: Inhibition of O‐GlcNAcylation protects from Shiga toxin‐mediated cell injury and lethality in host
Source: EMBO Mol Med. 2021 Nov 29;14(1):e14678. doi: 10.15252/emmm.202114678 (PMC8749473; doi:10.15252/emmm.202114678)
Supplement: Supplementary file 1 — Expanded View Figures PDF [file EMMM-14-e14678-s010.pdf]

Expanded View Figures

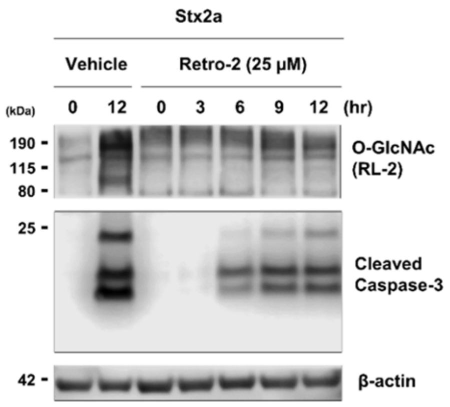

Figure EV1. Inhibition of retrograde trafficking blocks the aberrant Stx2a-induced increase in cellular O-GlcNAcylation and consequently delays caspase-3 activation.

Representative western blot showing changes in O-GlcNAcylation and caspase-3 activation in THP-1 cells treated with Stx2a (10 ng/ml) for 12 h in the presence or absence of the retrograde trafficking inhibitor Retro-2 (25 μM final).

Source data are available online for this figure.

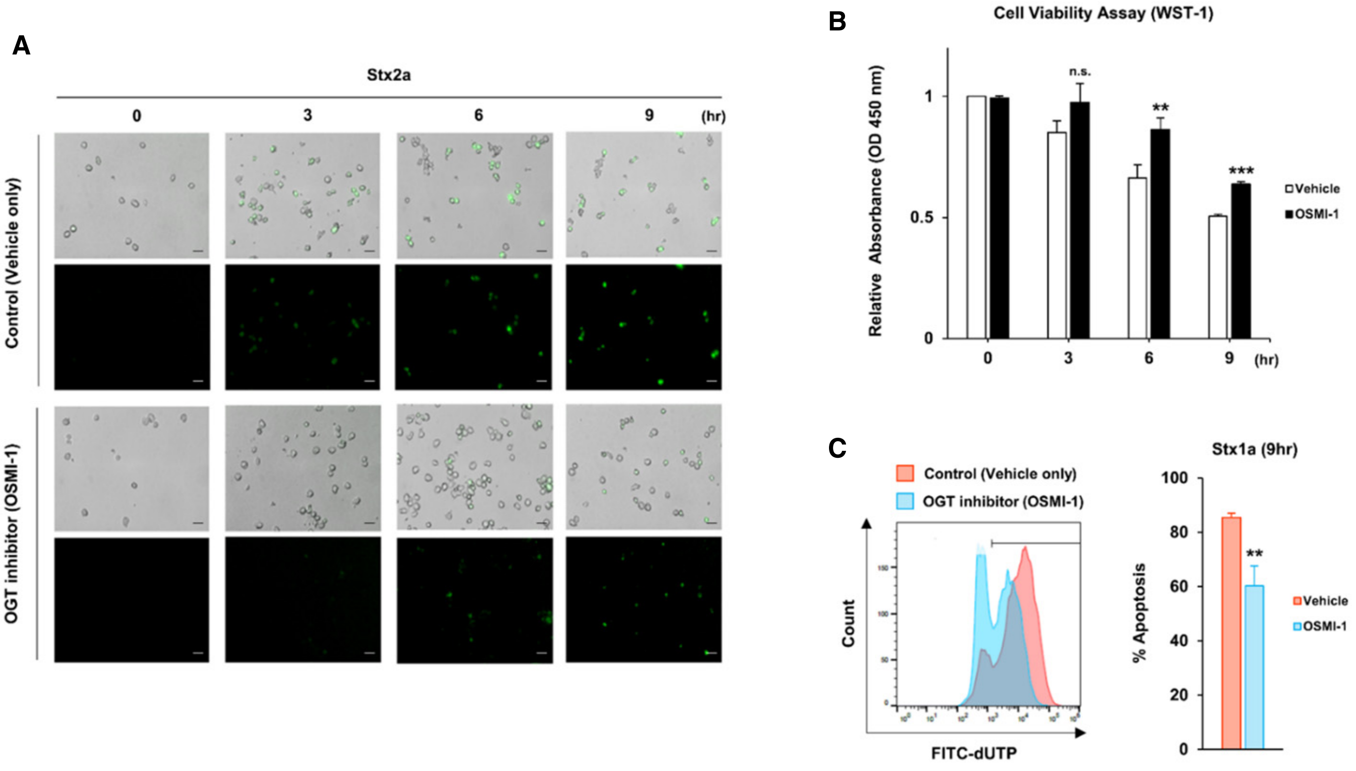

Figure EV2. Suppression of Stx2a- and Stx1a-induced O-GlcNAcylation rescues cells from apoptosis.

A Representative images showing TUNEL staining of Stx2a-treated THP-1 cells cultured in the presence of OSMI-1 or the vehicle control at 0, 3, 6, and 9 h time point each. FITC-dUTP staining (green fluorescence) in the TUNEL assay indicates active progression of apoptosis. Scale bars: 40 μm.

B WST-1 dye-based cell viability assay of Stx2a-exposed THP-1 cells treated with or without OSMI-1 (10 μM final) at 0, 3, 6, and 9 h time point each (n = 3 biological replicates).

C Representative flow cytometric plot showing apoptosis progression in THP-1 cells detected by TUNEL upon treatment with Stx1a (100 ng/ml) in the presence or absence of OSMI-1 (10 μM final), and quantification of the percentage of apoptotic cells at 9 h (n = 3 biological replicates). The effects of OSMI-1 treatment were compared with those of the vehicle (DMSO) control.

Data information: Error bars for bar graphs are presented as mean ± SD Statistical analysis was performed using two-tailed Student's t-test. *$P < 0.05$; **$P < 0.01$; and ***$P < 0.001$.
Source data are available online for this figure.

 © 2021 The Authors

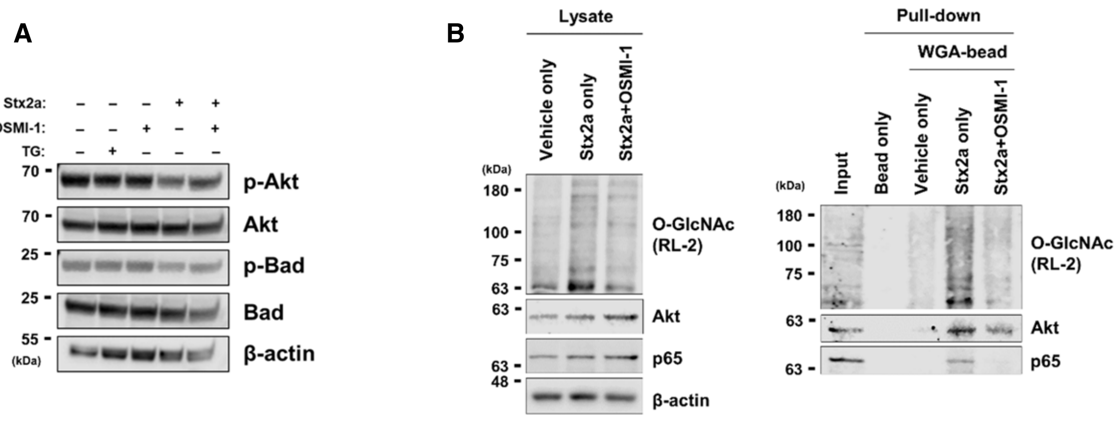

Figure EV3. Akt and p65 were directly O-GlcNAcylated in Stx2a-treated THP-1 cells.

A Representative western blot showing changes in phosphorylation status of Akt or Bad in THP-1 cells treated with Stx2a (10 ng/ml) for 3 h in the presence or absence of OSMI-1 (10 μM, final) or OGA inhibitor Thiamet G (2 μM, final).

B Representative western blot images, before (left) and after (right) pull down using WGA-lectin conjugated to agarose beads or bead-only control, to determine O-GlcNAc attachment to Akt and p65 in lysates from Stx2a (10 ng/ml)-exposed THP-1 cells for 9 h in the presence or absence of OSMI-1 (10 μM, final).

Source data are available online for this figure.

A Immune response

Gene	Fold change over (-)Stx2a (+)Stx2a Vehicle	Fold change over (-)Stx2a (+)Stx2a OSMI-1	OSMI-1 /Vehicle
UBD	35.208	14.477	0.411
LCP1	25.662	13.729	0.535
NPPB	16.079	5.943	0.37
CXCL8	14.602	3.847	0.263
LTB	13.403	5.696	0.425
IL34	11.623	6.627	0.57
APOBEC3B	10.774	7.075	0.657
TNFSF18	10.355	3.073	0.297
EBI3	8.726	2.976	0.341
IL23A	7.892	4.689	0.594
IL1RL1	7.573	4.151	0.548
BCL3	7.229	4.498	0.622
CX3CL1	7.005	4.178	0.596
GLIPR1	5.550	3.318	0.598
CCL20	5.519	3.367	0.61
PTX3	4.763	2.210	0.464
LIME1	4.513	2.741	0.607
PIK3CG	4.458	2.830	0.635
IL32	4.362	2.853	0.654
KYNU	4.156	2.691	0.647
CCL2	3.446	1.530	0.444
IL6	3.318	1.488	0.448
IL7R	2.819	1.219	0.432
GBP1	2.806	1.677	0.598
CXCL11	2.629	1.597	0.607
IFI27	2.602	1.550	0.596
BCL2	2.467	1.617	0.655
CXCL1	2.278	0.753	0.33
SEMA3C	2.049	1.330	0.649

Gene	Fold change over (-)Stx2a (+)Stx2a Vehicle	Fold change over (-)Stx2a (+)Stx2a OSMI-1	OSMI-1 /Vehicle
TNF	1.894	1.232	0.65
IST1	1.888	1.244	0.659
ITGB2	1.731	1.120	0.647
ICOSLG	1.685	2.704	1.605
TNFAIP6	1.643	0.998	0.608
OAS3	1.641	1.013	0.617
IFI6	1.533	0.618	0.403
TRIM29	1.401	0.752	0.537
ITM2A	1.189	0.653	0.549
EDN1	1.114	0.642	0.576
RAB9B	1.054	0.683	0.648
CXCL6	0.962	0.593	0.617
SEMA7A	0.880	0.575	0.653
TEC	0.868	1.342	1.546
CSF2	0.857	0.200	0.234
HSPA1A	0.854	1.302	1.524
HSPA1B	0.832	1.369	1.645
ULBP3	0.830	1.491	1.795
TRIM68	0.815	1.232	1.513
RHOG	0.704	0.439	0.624
IFI30	0.573	0.924	1.612
VCAM1	0.559	0.360	0.643
S100A7	0.442	1.100	2.49
SIGLEC15	0.442	0.285	0.644
PTGDS	0.334	0.662	1.983
RAB20	0.252	0.443	1.751
FTL	0.252	0.390	1.551
FTH1	0.150	0.233	1.553

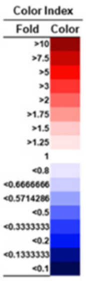

B Inflammatory response

Gene	(-)Stx2a OSMI-1 /Vehicle
PROK2	1
CXCL8	0.63
IL34	1
IL23A	0.932
CX3CL1	1.205
CCL20	0.995
PTX3	0.793
PIK3CG	2.081
CCL2	0.82
IL6	0.551
CXCL11	1
CXCL1	0.557
TSPAN2	0.653
TNF	1.161
PTGS1	1.045
ALOX5AP	0.658
ITGB2	0.966
TNFAIP6	0.771
NOX4	0.826
UGT1A1	0.981
CXCL6	0.777
SEMA7A	0.895
ITGB6	0.995
VCAM1	0.977
HMOX1	1.338

Apoptotic process

Gene	(-)Stx2a OSMI-1 /Vehicle
TRAF1	1.317
TNFRSF9	1.289
TOP2A	0.995
BCL3	1.057
PIK3CG	2.081
IL6	0.551
IFI27	1.536
BCL2	0.942
TNFRSF19	0.849
TGFB2	0.645
CAV1	0.910
INHBA	0.646
TNF	1.161
PPP1R13L	0.859
ITGB2	0.966
GDF6	0.459
IFI6	1.028
PARP1	1.006
HK2	0.869
TP53INP1	1.154
SGK1	0.552
DNASE2	1.126
SST	0.404
HMOX1	1.338
MAL	1.124
TNFRSF11B	0.736

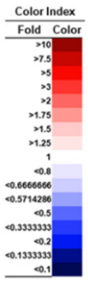

Figure EV4. Stx2a-mediated immune responses in primary human renal cells are regulated through O-GlcNAcylation.

A Heatmaps representing the comparative expression levels for DEGs in the presence or absence of OSMI-1 treatment upon Stx2a intoxication related to the immune responses.

B Heatmaps representing the comparative expression levels for DEGs in the presence or absence of OSMI-1 treatment without Stx2a exposure related to the inflammatory response (left) and apoptotic process (right).

Data information: The numbers within the tables are normalized gene expression level compared to HRPTEpi cells maintained in the absence of either Stx2a or OSMI-1. Expression values are represented with red (upregulation) or blue (downregulation) color using FPKM values by Cufflinks; the cutoffs used a fold change of at least 1.5 followed by pairwise comparison and Student's *t*-test with a Benjamini and Hochberg correction. The FPKM values were normalized using EdgeR within R and visualized using ExDEGA. Data are the means from two independent replicates.

© 2021 The Authors

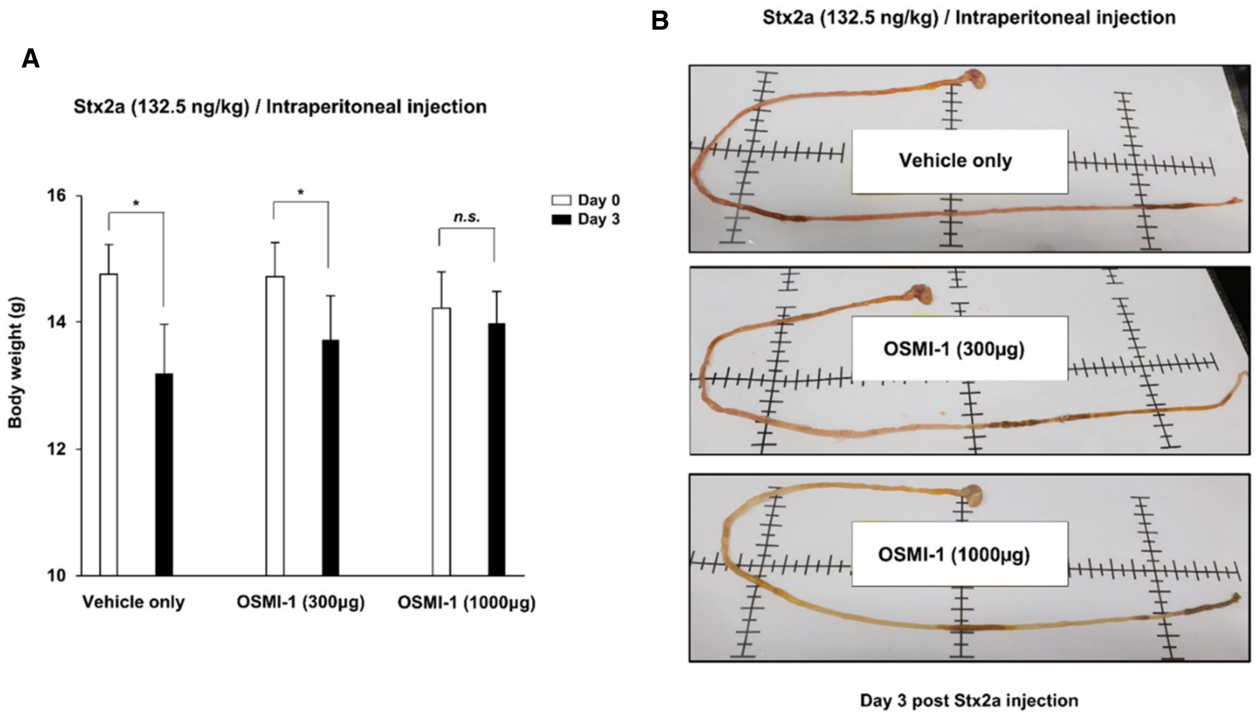

Figure EV5. Treatment with an OGT inhibitor improves severe loss of body weight due to the hemorrhagic symptoms in the intestines of mice challenged with Stx2a.

A Body weight changes in mice challenged with Stx2a in the presence or absence of OSMI-1 (300 or 1,000 μg/mouse). Data are presented as mean ± SD (n = 5 biological replicates per group, two-tailed Student's t-test). Comparisons between days 0 and 3 were indicated for each group. *$P < 0.05$; **$P < 0.01$; and ***$P < 0.001$.

B Representative images of the intestines of mice at day 3 after Stx2a injection and combinatorial treatment with two doses of OSMI-1 or the vehicle alone.

Source data are available online for this figure.

© 2021 The Authors
