# Supplementary material for: Inhibition of O‐GlcNAcylation protects from Shiga toxin‐mediated cell injury and lethality in host
Source: EMBO Mol Med. 2021 Nov 29;14(1):e14678. doi: 10.15252/emmm.202114678 (PMC8749473; doi:10.15252/emmm.202114678)
Supplement: Supplementary file 3 — Source Data for Expanded View [file EMMM-14-e14678-s009.zip › Source_data_Figure_EV1/Blots_Figure_EV1.pptx]

## Slide 1
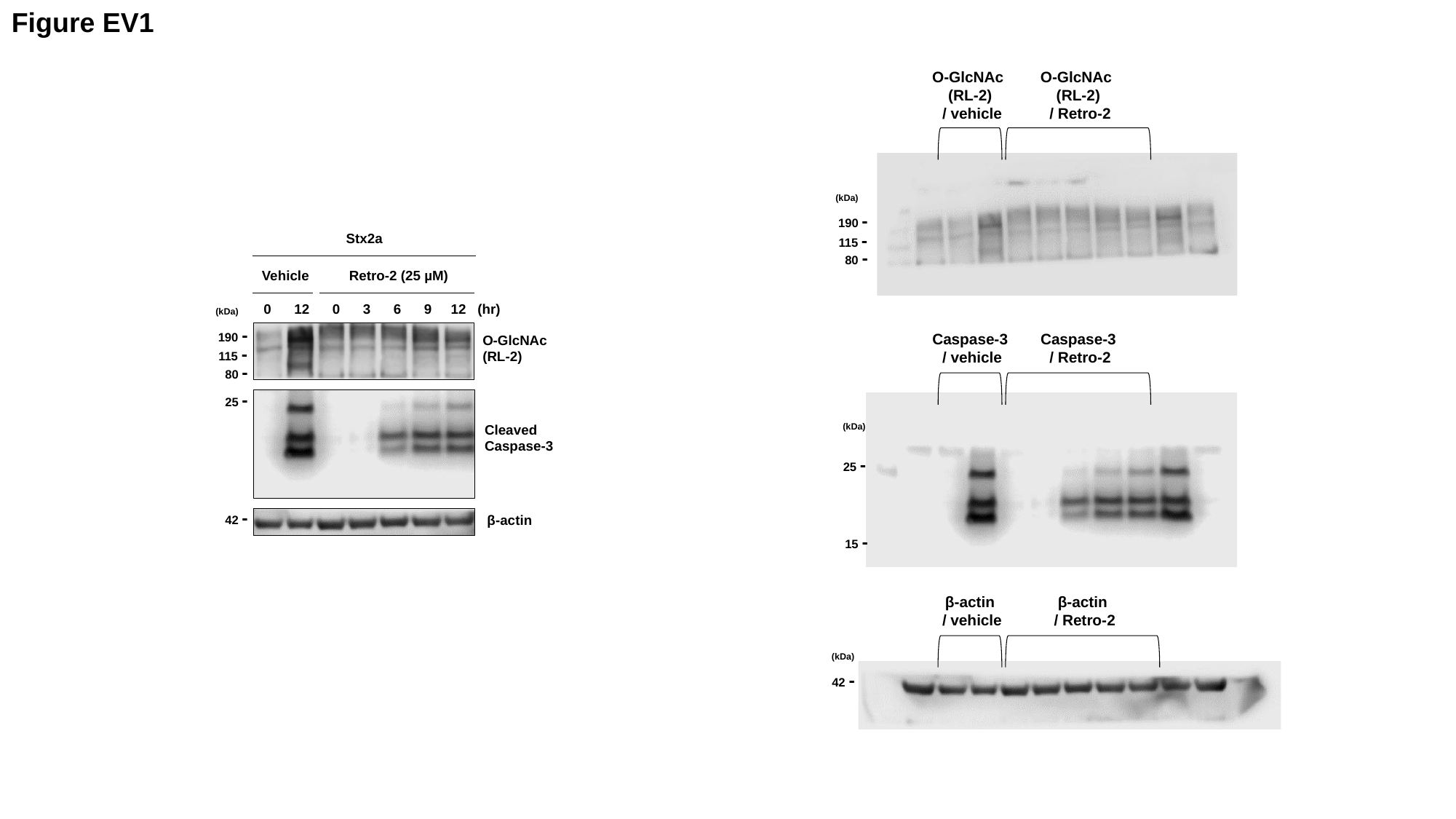

Figure EV1
O-GlcNAc
(RL-2)
 / vehicle
O-GlcNAc
(RL-2)
 / Retro-2
(kDa)
190 -
115 -
Stx2a
80 -
Vehicle
Retro-2 (25 µM)
 0 12 0 3 6 9 12 (hr)
(kDa)
190 -
Caspase-3
 / vehicle
Caspase-3
 / Retro-2
O-GlcNAc
(RL-2)
115 -
80 -
25 -
(kDa)
Cleaved
Caspase-3
25 -
42 -
β-actin
15 -
β-actin
 / vehicle
β-actin
 / Retro-2
(kDa)
42 -
