# Supplementary material for: Inhibition of O‐GlcNAcylation protects from Shiga toxin‐mediated cell injury and lethality in host
Source: EMBO Mol Med. 2021 Nov 29;14(1):e14678. doi: 10.15252/emmm.202114678 (PMC8749473; doi:10.15252/emmm.202114678)
Supplement: Supplementary file 3 — Source Data for Expanded View [file EMMM-14-e14678-s009.zip › Source_data_Figure_EV3/Blots_Figure_EV3.pptx]

## Slide 1
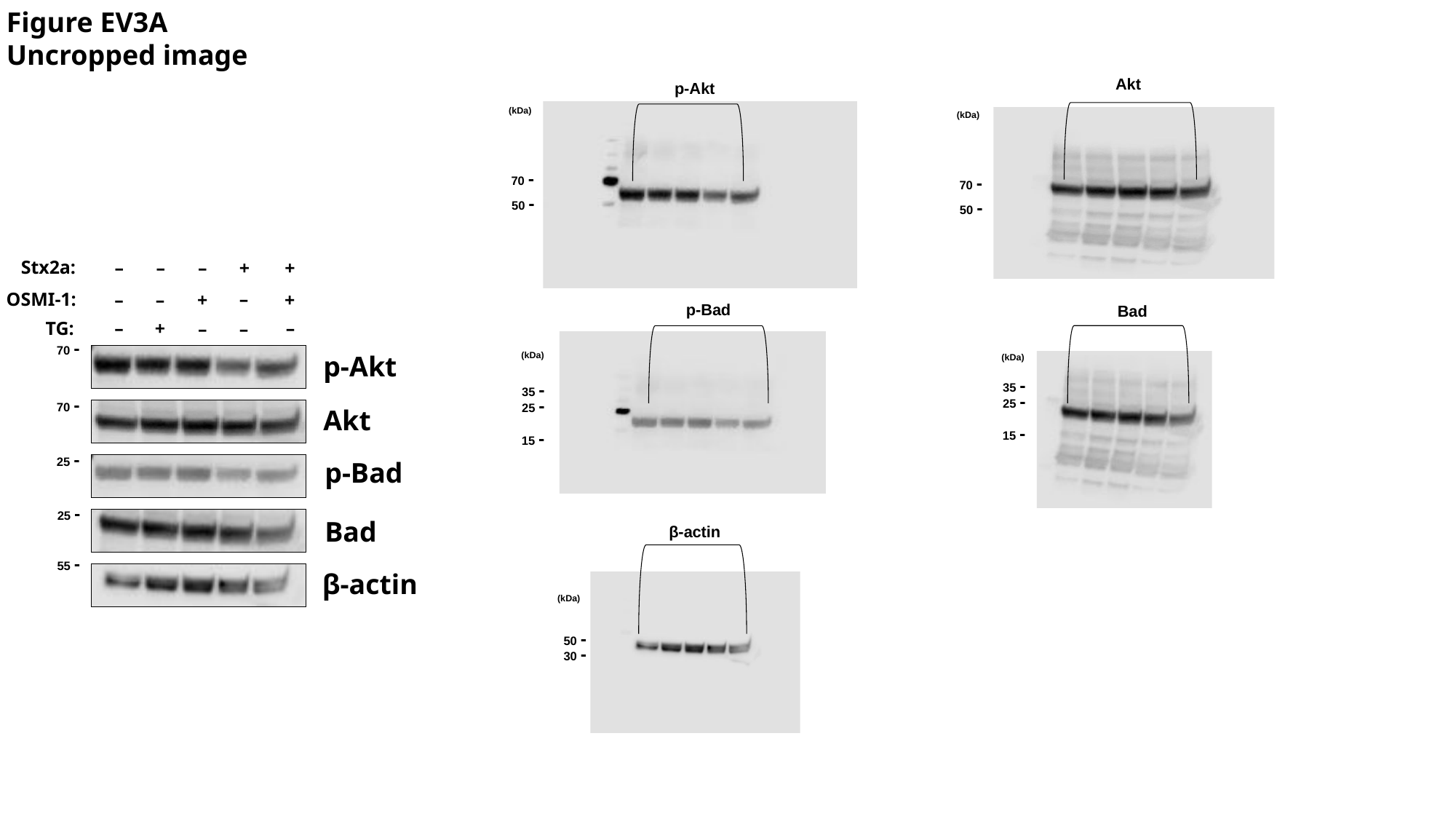

Figure EV3A
Uncropped image
Akt
p-Akt
(kDa)
(kDa)
70 -
70 -
50 -
50 -
Stx2a:
–
–
–
+
+
–
OSMI-1:
–
–
+
+
p-Bad
Bad
–
–
+
TG:
–
–
70 -
(kDa)
p-Akt
(kDa)
35 -
35 -
25 -
70 -
25 -
Akt
15 -
15 -
25 -
p-Bad
25 -
Bad
β-actin
55 -
β-actin
(kDa)
50 -
30 -

## Slide 2
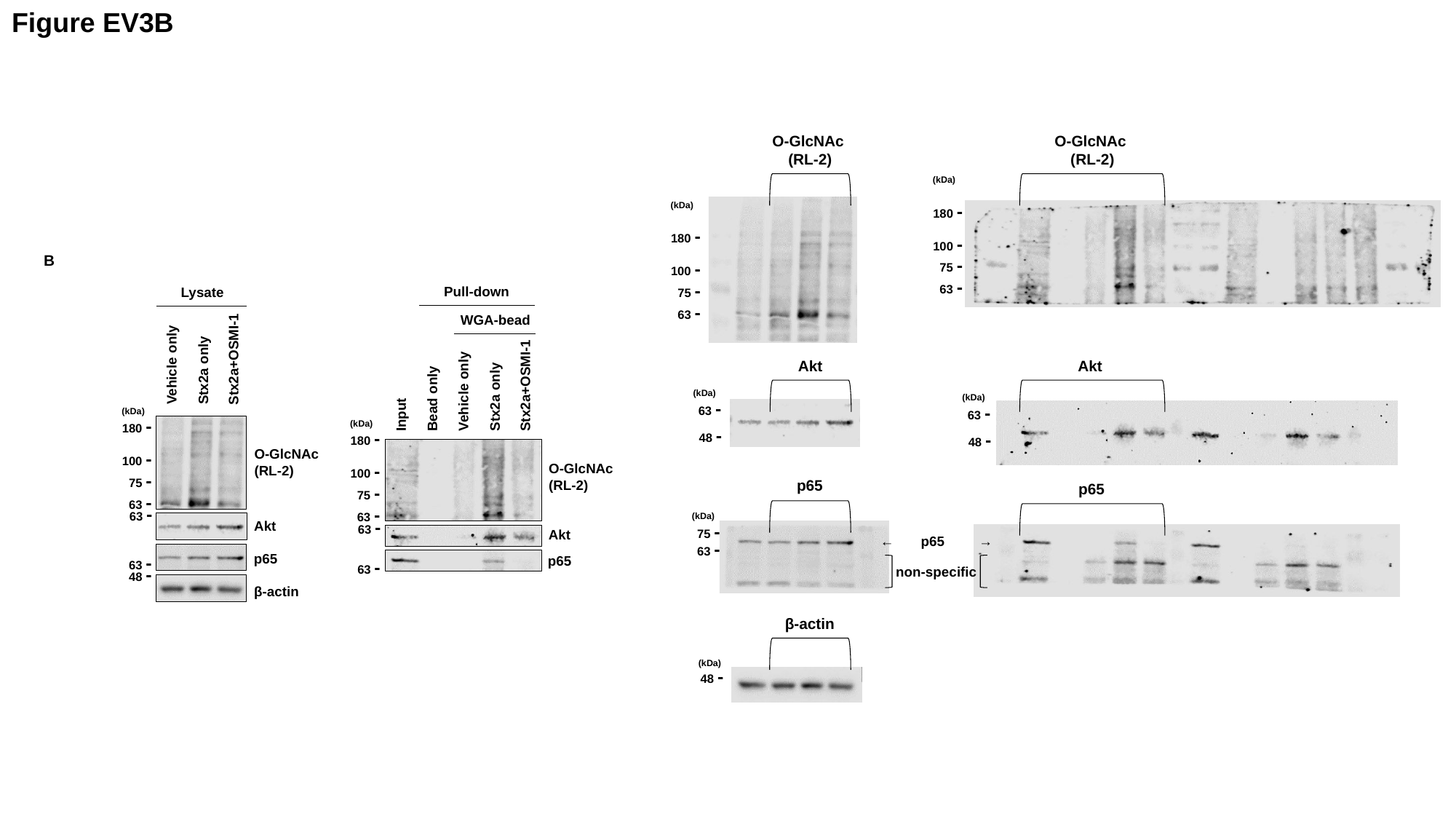

Figure EV3B
O-GlcNAc
(RL-2)
O-GlcNAc
(RL-2)
(kDa)
(kDa)
180 -
100 -
75 -
63 -
180 -
100 -
75 -
63 -
B
Pull-down
Lysate
WGA-bead
Stx2a+OSMI-1
Akt
Akt
Vehicle only
Stx2a only
Stx2a+OSMI-1
Vehicle only
(kDa)
Stx2a only
Bead only
(kDa)
63 -
63 -
(kDa)
Input
180 -
100 -
75 -
63 -
(kDa)
48 -
180 -
100 -
75 -
63 -
48 -
O-GlcNAc
(RL-2)
O-GlcNAc
(RL-2)
p65
p65
63 -
(kDa)
63 -
Akt
75 -
Akt
← p65 →
63 -
p65
63 -
p65
63 -
non-specific
48 -
β-actin
β-actin
(kDa)
48 -
