# Supplementary figures and images for: Inhibition of O‐GlcNAcylation protects from Shiga toxin‐mediated cell injury and lethality in host
Source: EMBO Mol Med. 2021 Nov 29;14(1):e14678. doi: 10.15252/emmm.202114678 (PMC8749473; doi:10.15252/emmm.202114678)

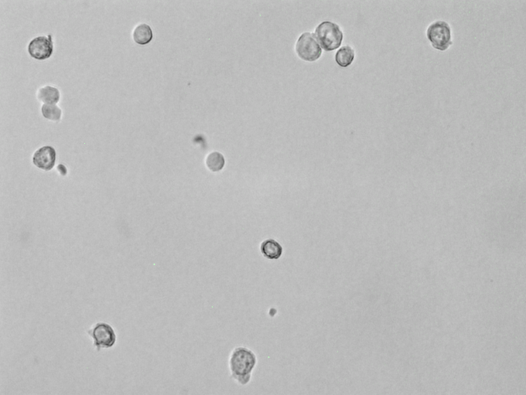

Supplement: Supplementary file 3 — Source Data for Expanded View [file EMMM-14-e14678-s009.zip › Source_data_Figure_EV2/Fig_EV2._TUNEL_assay_Microscopy_uncropped_images/OSMI-1_0h-1.tif]

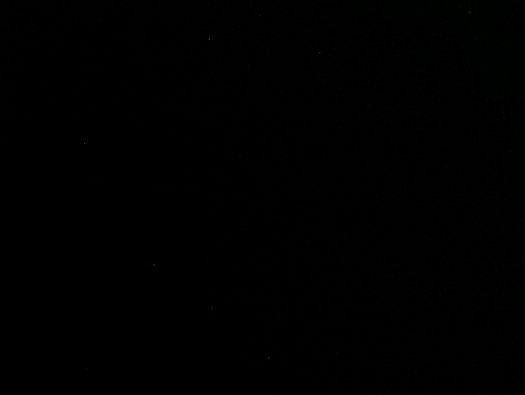

Supplement: Supplementary file 3 — Source Data for Expanded View [file EMMM-14-e14678-s009.zip › Source_data_Figure_EV2/Fig_EV2._TUNEL_assay_Microscopy_uncropped_images/OSMI-1_0h-2.tif]

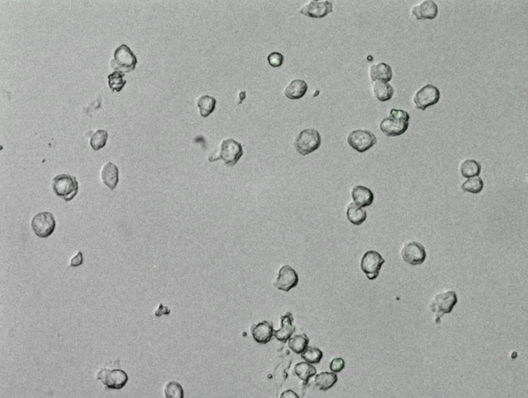

Supplement: Supplementary file 3 — Source Data for Expanded View [file EMMM-14-e14678-s009.zip › Source_data_Figure_EV2/Fig_EV2._TUNEL_assay_Microscopy_uncropped_images/OSMI-1_3h-1.tif]

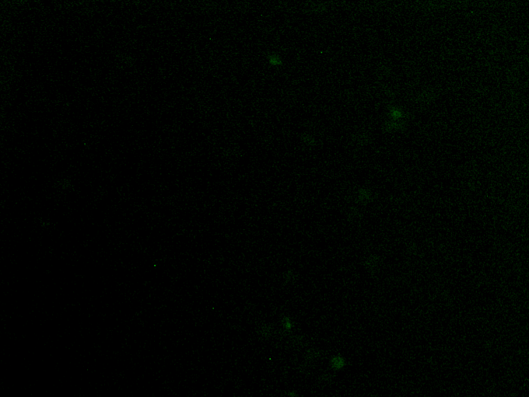

Supplement: Supplementary file 3 — Source Data for Expanded View [file EMMM-14-e14678-s009.zip › Source_data_Figure_EV2/Fig_EV2._TUNEL_assay_Microscopy_uncropped_images/OSMI-1_3h-2.tif]

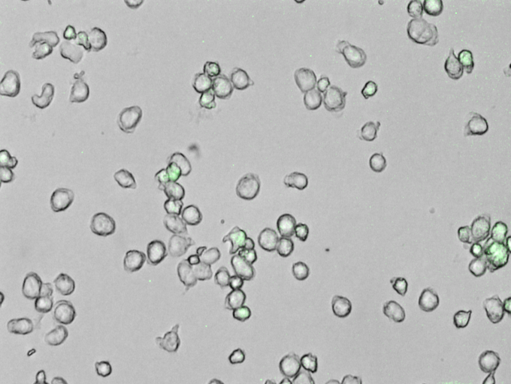

Supplement: Supplementary file 3 — Source Data for Expanded View [file EMMM-14-e14678-s009.zip › Source_data_Figure_EV2/Fig_EV2._TUNEL_assay_Microscopy_uncropped_images/OSMI-1_6h-1.tif]

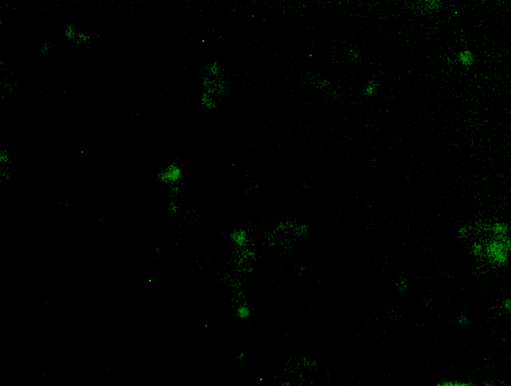

Supplement: Supplementary file 3 — Source Data for Expanded View [file EMMM-14-e14678-s009.zip › Source_data_Figure_EV2/Fig_EV2._TUNEL_assay_Microscopy_uncropped_images/OSMI-1_6h-2.tif]

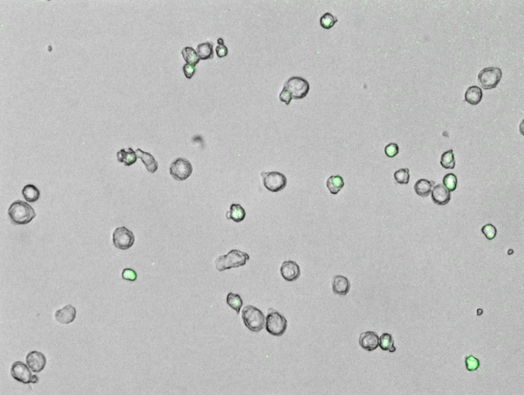

Supplement: Supplementary file 3 — Source Data for Expanded View [file EMMM-14-e14678-s009.zip › Source_data_Figure_EV2/Fig_EV2._TUNEL_assay_Microscopy_uncropped_images/OSMI-1_9h-1.tif]

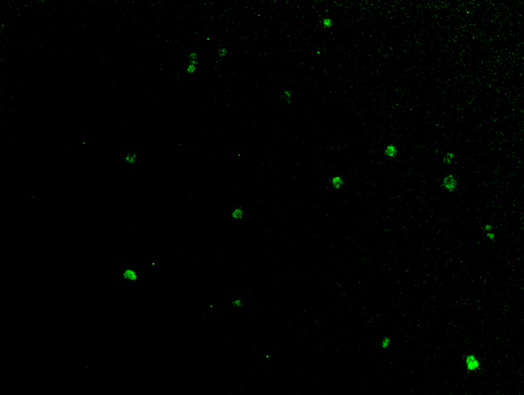

Supplement: Supplementary file 3 — Source Data for Expanded View [file EMMM-14-e14678-s009.zip › Source_data_Figure_EV2/Fig_EV2._TUNEL_assay_Microscopy_uncropped_images/OSMI-1_9h-2.tif]

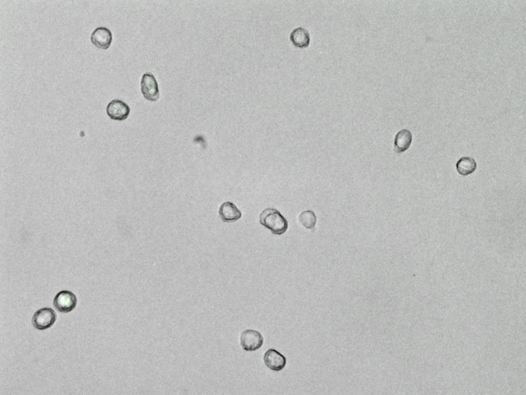

Supplement: Supplementary file 3 — Source Data for Expanded View [file EMMM-14-e14678-s009.zip › Source_data_Figure_EV2/Fig_EV2._TUNEL_assay_Microscopy_uncropped_images/Vehicle_0h-1.tif]

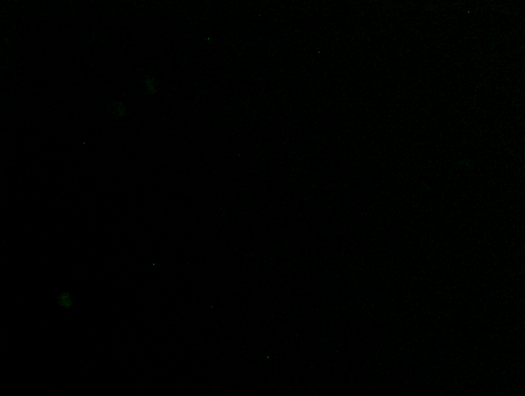

Supplement: Supplementary file 3 — Source Data for Expanded View [file EMMM-14-e14678-s009.zip › Source_data_Figure_EV2/Fig_EV2._TUNEL_assay_Microscopy_uncropped_images/Vehicle_0h-2.tif]

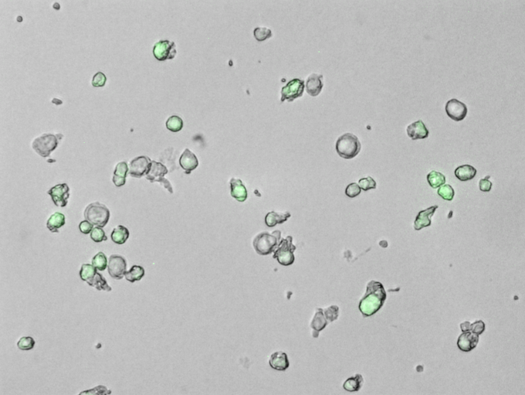

Supplement: Supplementary file 3 — Source Data for Expanded View [file EMMM-14-e14678-s009.zip › Source_data_Figure_EV2/Fig_EV2._TUNEL_assay_Microscopy_uncropped_images/Vehicle_3h-1.tif]

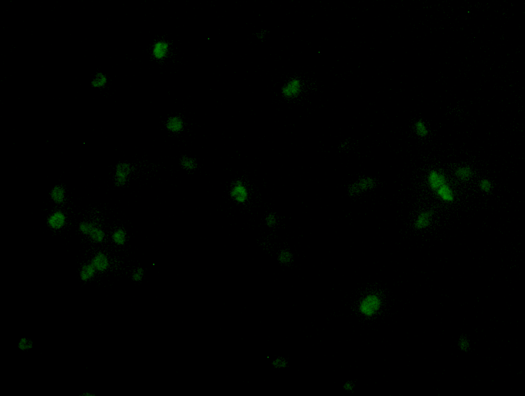

Supplement: Supplementary file 3 — Source Data for Expanded View [file EMMM-14-e14678-s009.zip › Source_data_Figure_EV2/Fig_EV2._TUNEL_assay_Microscopy_uncropped_images/Vehicle_3h-2.tif]

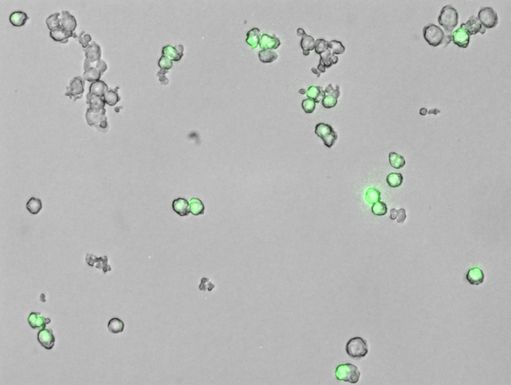

Supplement: Supplementary file 3 — Source Data for Expanded View [file EMMM-14-e14678-s009.zip › Source_data_Figure_EV2/Fig_EV2._TUNEL_assay_Microscopy_uncropped_images/Vehicle_6h-1.tif]

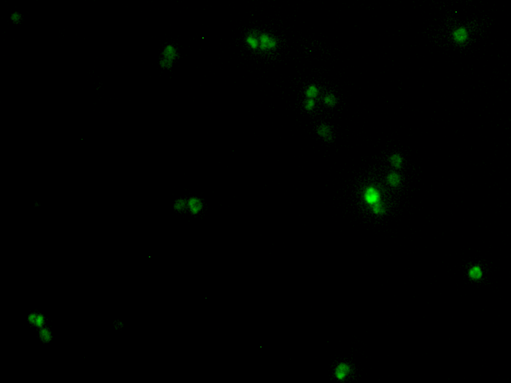

Supplement: Supplementary file 3 — Source Data for Expanded View [file EMMM-14-e14678-s009.zip › Source_data_Figure_EV2/Fig_EV2._TUNEL_assay_Microscopy_uncropped_images/Vehicle_6h-2.tif]

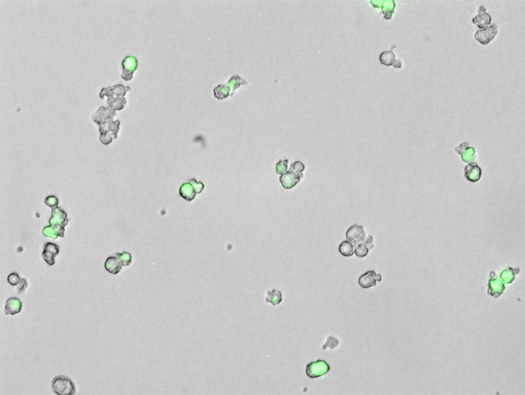

Supplement: Supplementary file 3 — Source Data for Expanded View [file EMMM-14-e14678-s009.zip › Source_data_Figure_EV2/Fig_EV2._TUNEL_assay_Microscopy_uncropped_images/Vehicle_9h-1.tif]

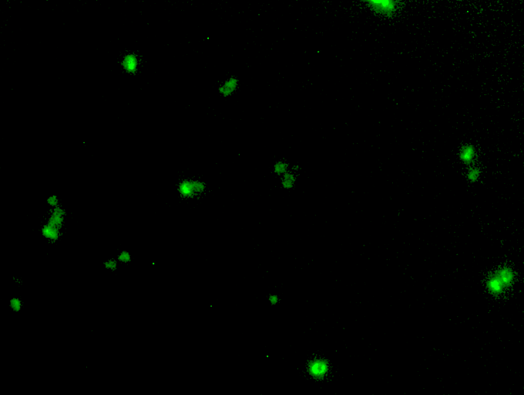

Supplement: Supplementary file 3 — Source Data for Expanded View [file EMMM-14-e14678-s009.zip › Source_data_Figure_EV2/Fig_EV2._TUNEL_assay_Microscopy_uncropped_images/Vehicle_9h-2.tif]

## Slide 1
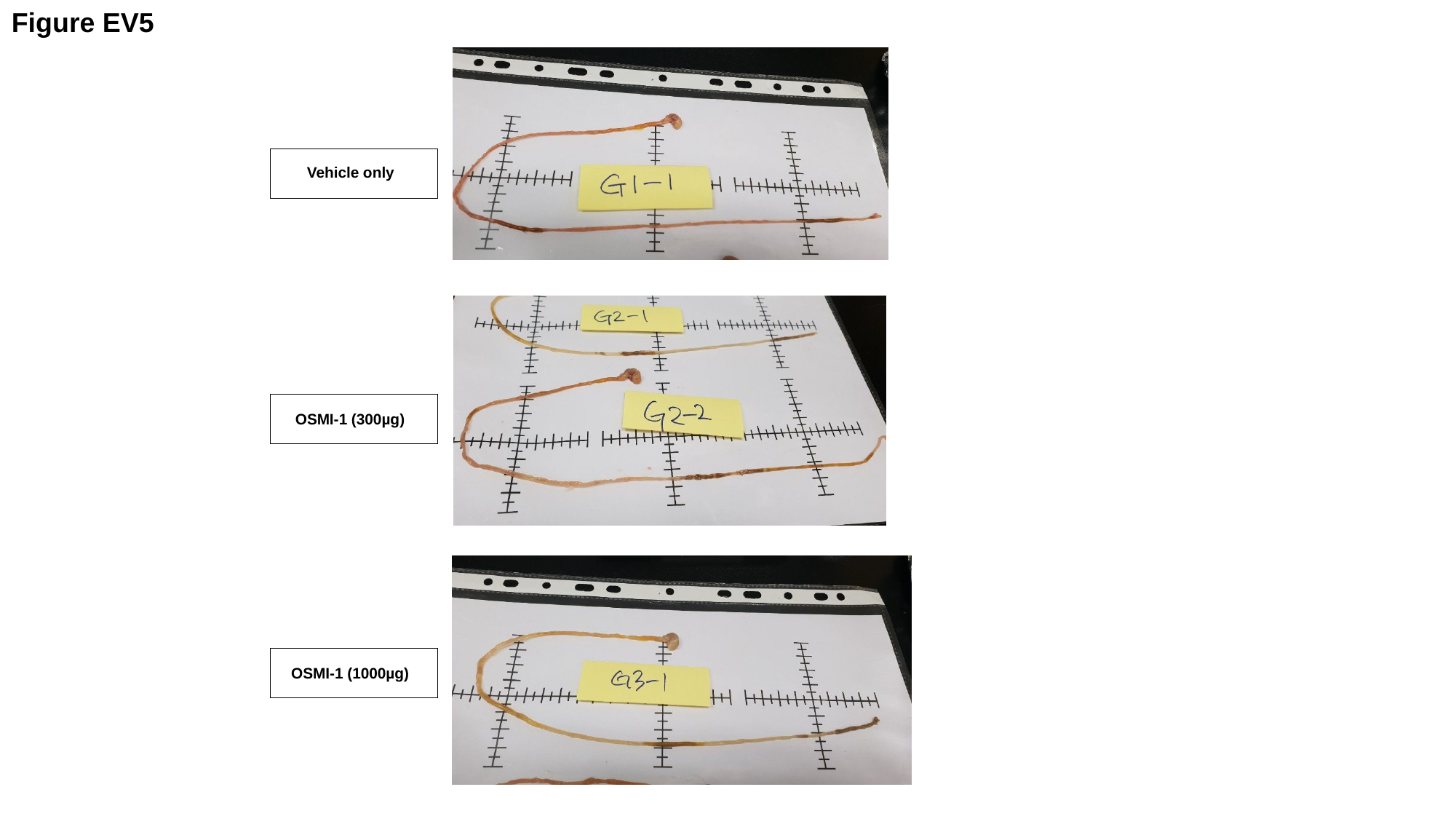

Figure EV5
Vehicle only
OSMI-1 (300µg)
OSMI-1 (1000µg)

Supplement: Supplementary file 3 — Source Data for Expanded View [file EMMM-14-e14678-s009.zip › Source_data_Figure_EV5/Source_data_uncropped_images_for_Fig_EV5B.pptx]

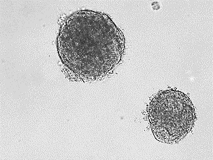

Supplement: Supplementary file 8 — Source Data for Figure 5 [file EMMM-14-e14678-s008.zip › 3D-Spheroids_Microscopy_uncropped_images/3D_HRPTEC/10_OSMI1_Stx2.tif]

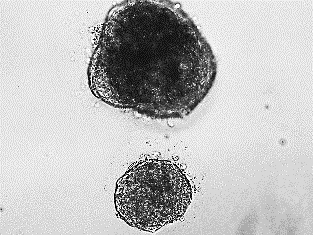

Supplement: Supplementary file 8 — Source Data for Figure 5 [file EMMM-14-e14678-s008.zip › 3D-Spheroids_Microscopy_uncropped_images/3D_HRPTEC/11_OSMI1_Stx2.tif]

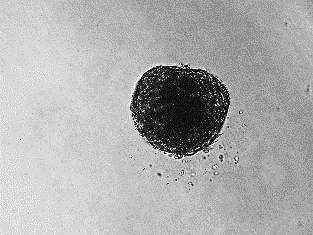

Supplement: Supplementary file 8 — Source Data for Figure 5 [file EMMM-14-e14678-s008.zip › 3D-Spheroids_Microscopy_uncropped_images/3D_HRPTEC/12_OSMI1_Stx2.tif]

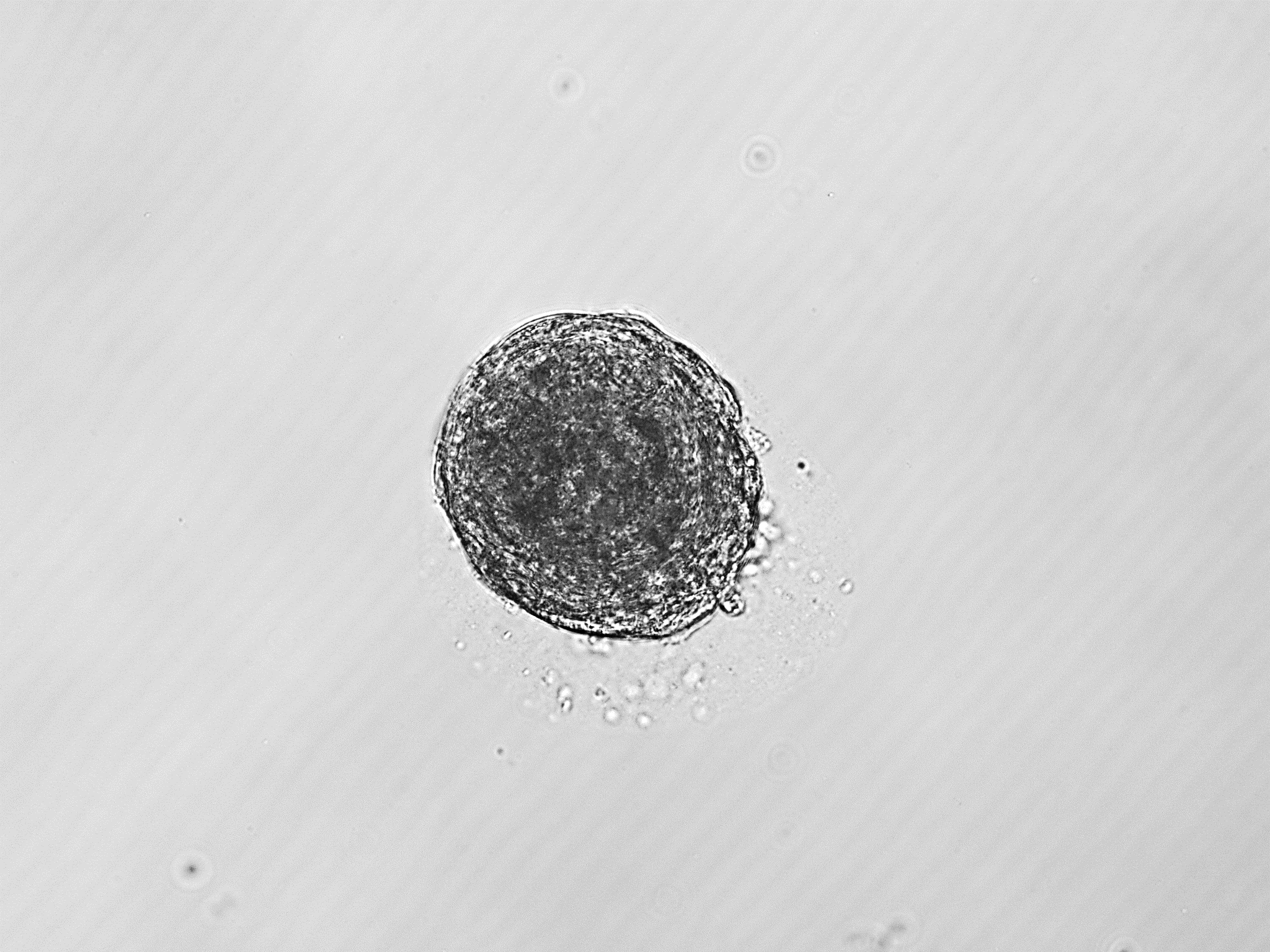

Supplement: Supplementary file 8 — Source Data for Figure 5 [file EMMM-14-e14678-s008.zip › 3D-Spheroids_Microscopy_uncropped_images/3D_HRPTEC/1_Vehicle_Control.tif]

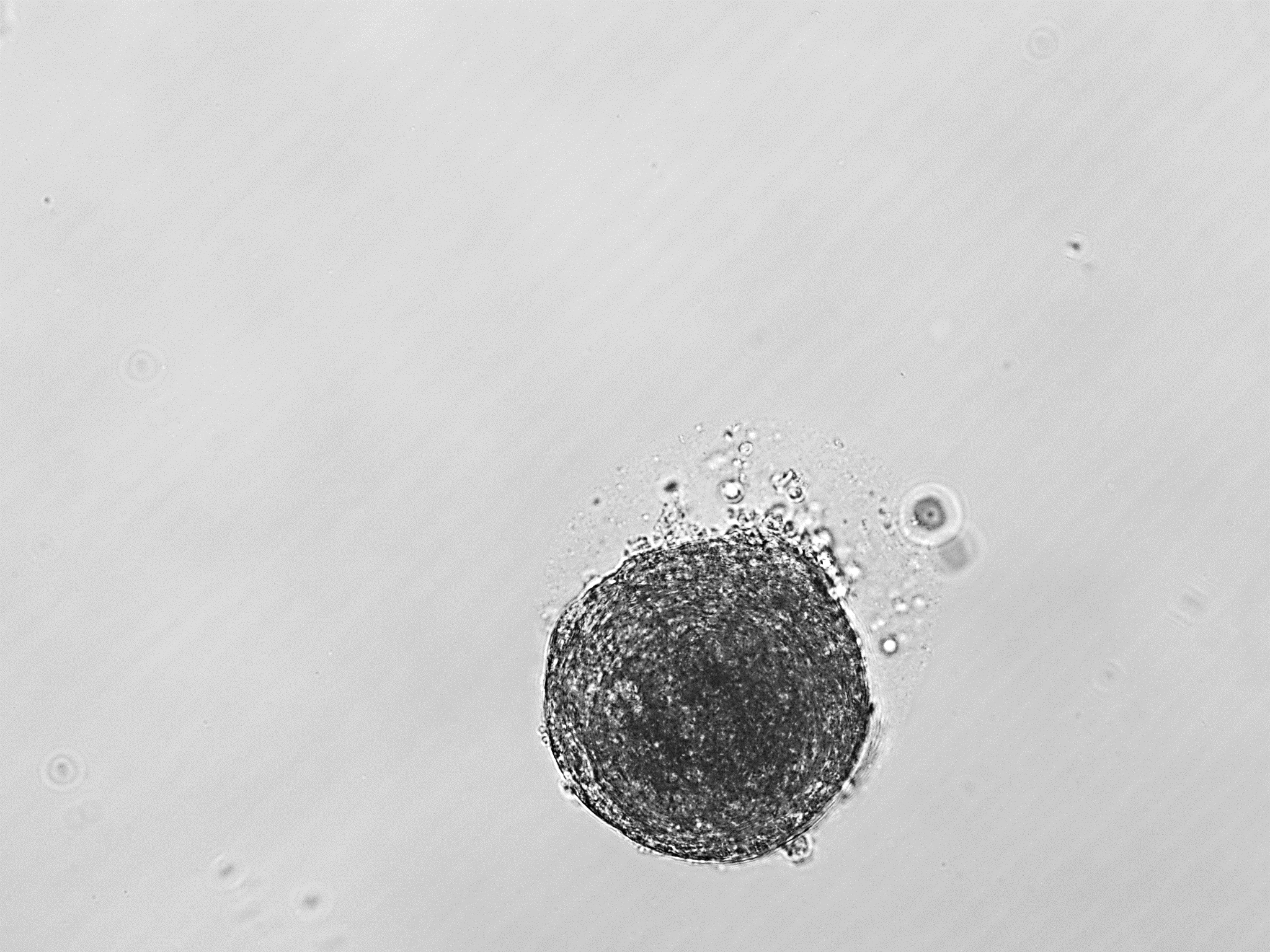

Supplement: Supplementary file 8 — Source Data for Figure 5 [file EMMM-14-e14678-s008.zip › 3D-Spheroids_Microscopy_uncropped_images/3D_HRPTEC/2_Vehicle_Control.tif]

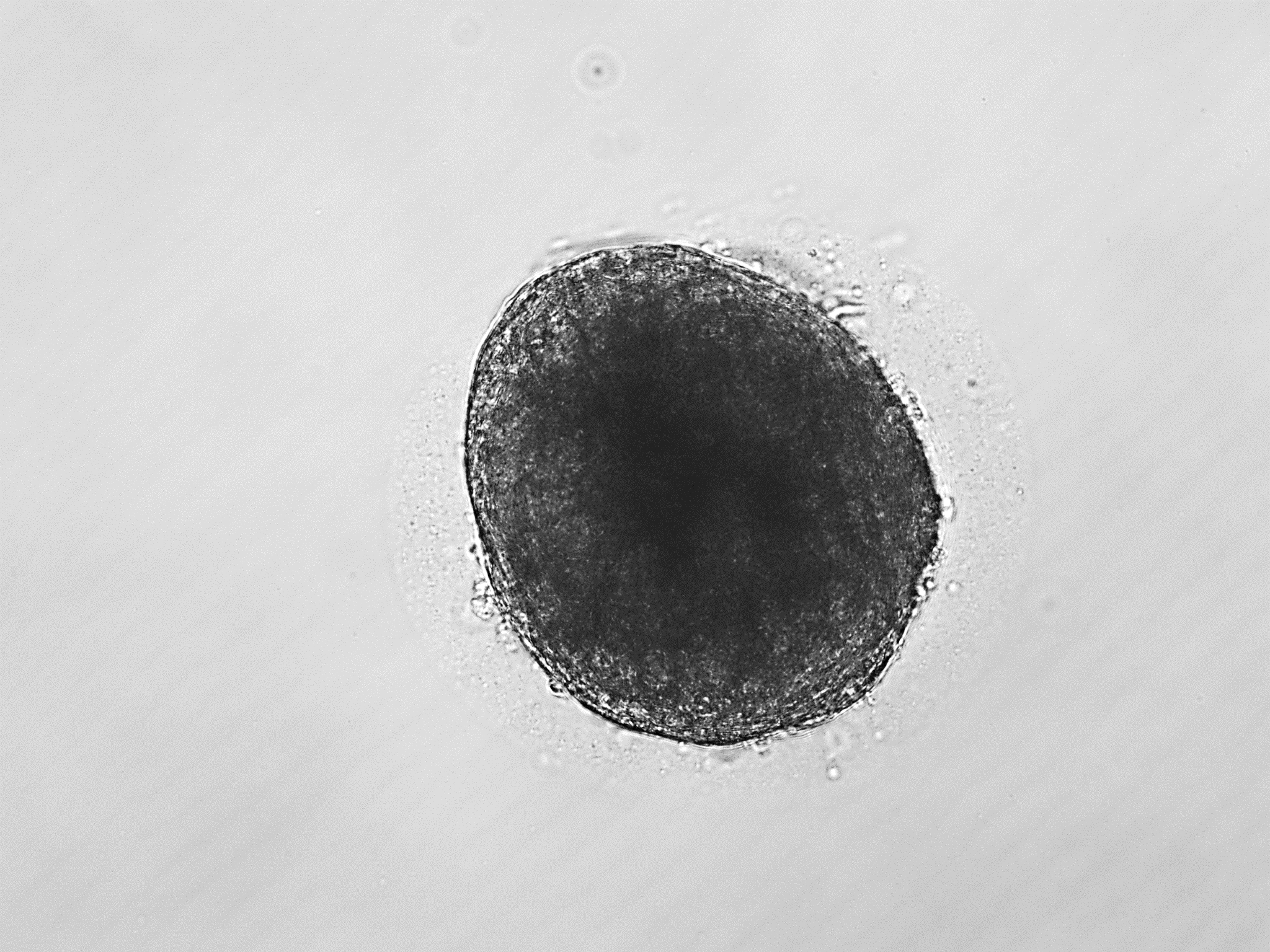

Supplement: Supplementary file 8 — Source Data for Figure 5 [file EMMM-14-e14678-s008.zip › 3D-Spheroids_Microscopy_uncropped_images/3D_HRPTEC/3_Vehicle_Control.tif]

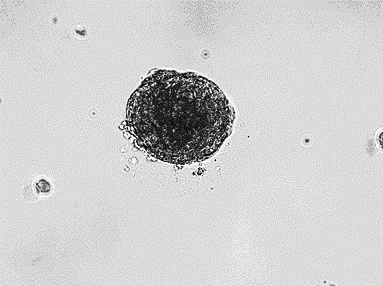

Supplement: Supplementary file 8 — Source Data for Figure 5 [file EMMM-14-e14678-s008.zip › 3D-Spheroids_Microscopy_uncropped_images/3D_HRPTEC/4_OSMI1_Control.tif]

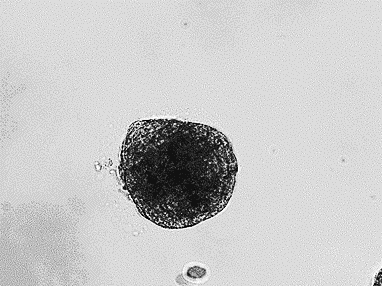

Supplement: Supplementary file 8 — Source Data for Figure 5 [file EMMM-14-e14678-s008.zip › 3D-Spheroids_Microscopy_uncropped_images/3D_HRPTEC/5_OSMI1_Control.tif]

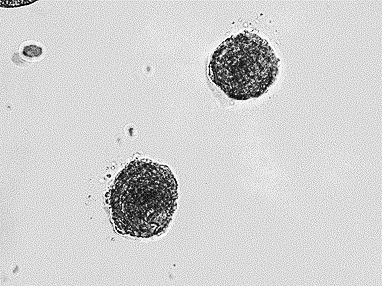

Supplement: Supplementary file 8 — Source Data for Figure 5 [file EMMM-14-e14678-s008.zip › 3D-Spheroids_Microscopy_uncropped_images/3D_HRPTEC/6_OSMI1_Control.tif]

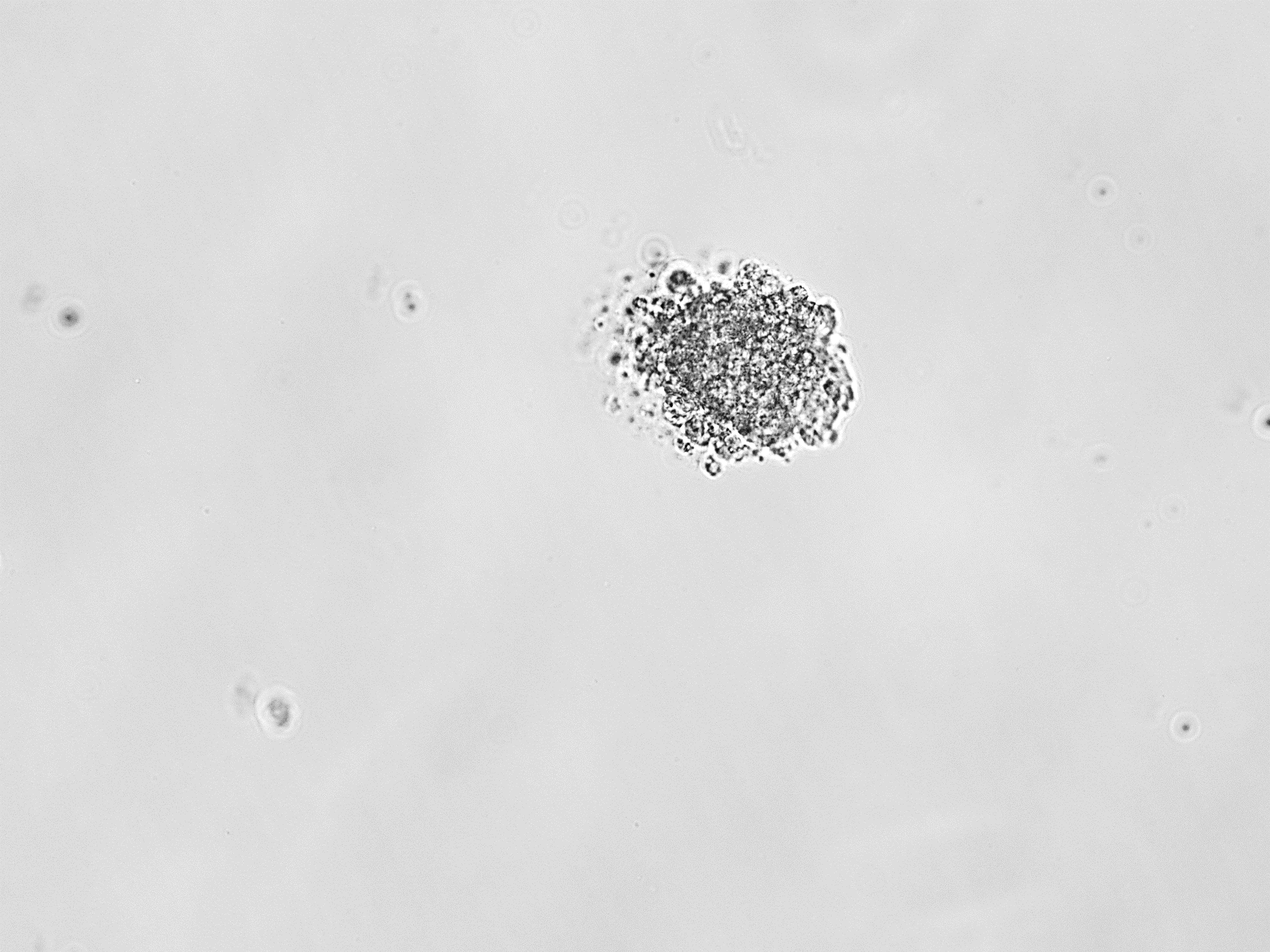

Supplement: Supplementary file 8 — Source Data for Figure 5 [file EMMM-14-e14678-s008.zip › 3D-Spheroids_Microscopy_uncropped_images/3D_HRPTEC/7_Vehicle_Stx2.tif]

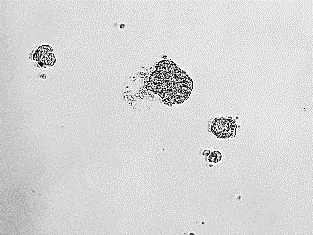

Supplement: Supplementary file 8 — Source Data for Figure 5 [file EMMM-14-e14678-s008.zip › 3D-Spheroids_Microscopy_uncropped_images/3D_HRPTEC/8_Vehicle_Stx2.tif]

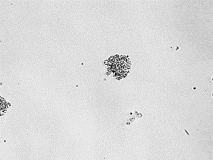

Supplement: Supplementary file 8 — Source Data for Figure 5 [file EMMM-14-e14678-s008.zip › 3D-Spheroids_Microscopy_uncropped_images/3D_HRPTEC/9_Vehicle_Stx2.tif]

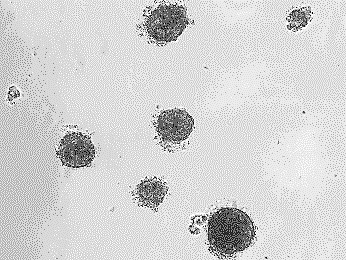

Supplement: Supplementary file 8 — Source Data for Figure 5 [file EMMM-14-e14678-s008.zip › 3D-Spheroids_Microscopy_uncropped_images/3D_Mini_Kidney/10_OSMI1_Stx2_M.K..tif]

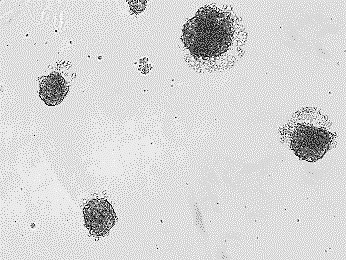

Supplement: Supplementary file 8 — Source Data for Figure 5 [file EMMM-14-e14678-s008.zip › 3D-Spheroids_Microscopy_uncropped_images/3D_Mini_Kidney/11_OSMI1_Stx2_M.K..tif]

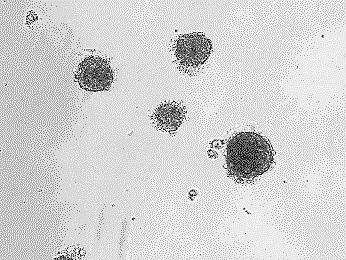

Supplement: Supplementary file 8 — Source Data for Figure 5 [file EMMM-14-e14678-s008.zip › 3D-Spheroids_Microscopy_uncropped_images/3D_Mini_Kidney/12_OSMI1_Stx2_M.K..tif]

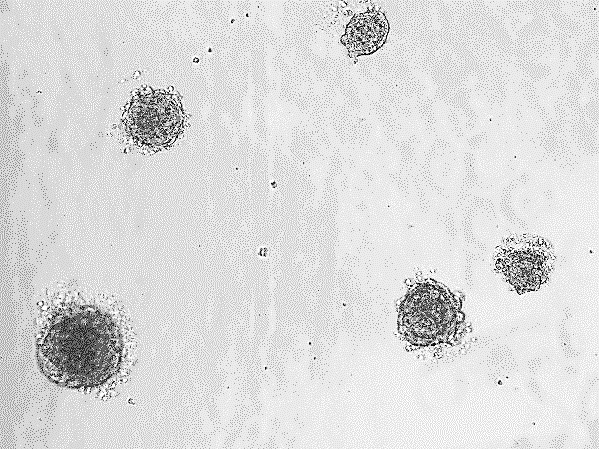

Supplement: Supplementary file 8 — Source Data for Figure 5 [file EMMM-14-e14678-s008.zip › 3D-Spheroids_Microscopy_uncropped_images/3D_Mini_Kidney/1_Vehicle_Control_M.K..tif]

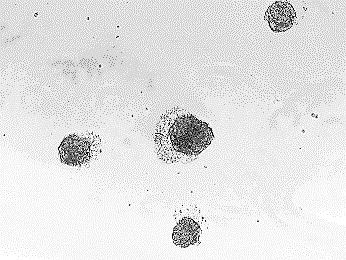

Supplement: Supplementary file 8 — Source Data for Figure 5 [file EMMM-14-e14678-s008.zip › 3D-Spheroids_Microscopy_uncropped_images/3D_Mini_Kidney/2_Vehicle_Control_M.K..tif]

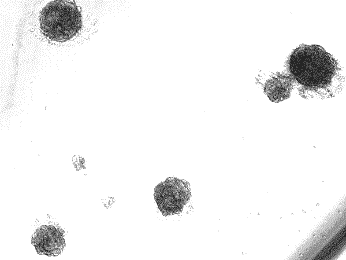

Supplement: Supplementary file 8 — Source Data for Figure 5 [file EMMM-14-e14678-s008.zip › 3D-Spheroids_Microscopy_uncropped_images/3D_Mini_Kidney/3_Vehicle_Control_M.K..tif]

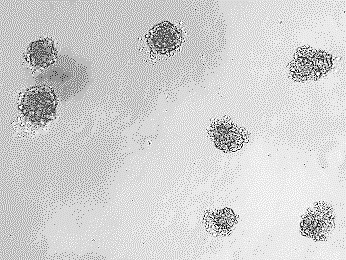

Supplement: Supplementary file 8 — Source Data for Figure 5 [file EMMM-14-e14678-s008.zip › 3D-Spheroids_Microscopy_uncropped_images/3D_Mini_Kidney/4_OSMI1_Control_M.K..tif]

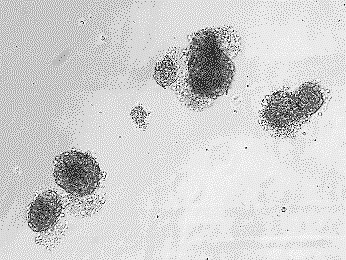

Supplement: Supplementary file 8 — Source Data for Figure 5 [file EMMM-14-e14678-s008.zip › 3D-Spheroids_Microscopy_uncropped_images/3D_Mini_Kidney/5_OSMI1_Control_M.K..tif]

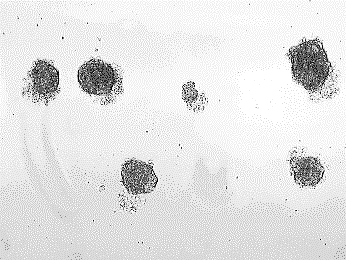

Supplement: Supplementary file 8 — Source Data for Figure 5 [file EMMM-14-e14678-s008.zip › 3D-Spheroids_Microscopy_uncropped_images/3D_Mini_Kidney/6_OSMI1_Control_M.K..tif]

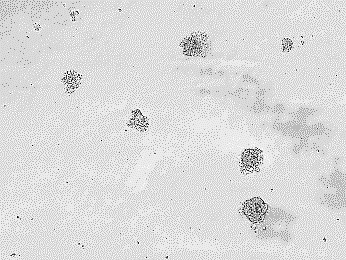

Supplement: Supplementary file 8 — Source Data for Figure 5 [file EMMM-14-e14678-s008.zip › 3D-Spheroids_Microscopy_uncropped_images/3D_Mini_Kidney/7_Vehicle_Stx2_M.K..tif]

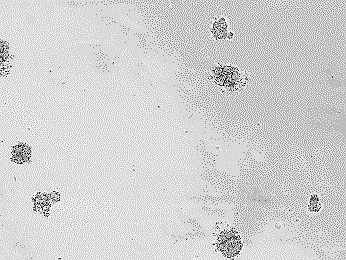

Supplement: Supplementary file 8 — Source Data for Figure 5 [file EMMM-14-e14678-s008.zip › 3D-Spheroids_Microscopy_uncropped_images/3D_Mini_Kidney/8_Vehicle_Stx2_M.K..tif]

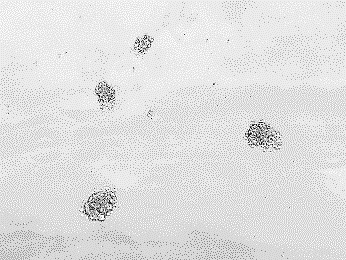

Supplement: Supplementary file 8 — Source Data for Figure 5 [file EMMM-14-e14678-s008.zip › 3D-Spheroids_Microscopy_uncropped_images/3D_Mini_Kidney/9_Vehicle_Stx2_M.K..tif]

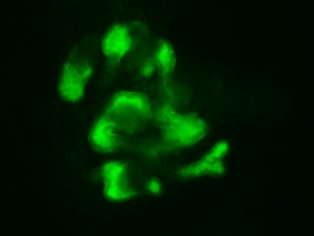

Supplement: Supplementary file 9 — Source Data for Figure 6 [file EMMM-14-e14678-s006.zip › Fig_6._Organoids_Microscopy_uncropped_images/A/LTL.jpg]

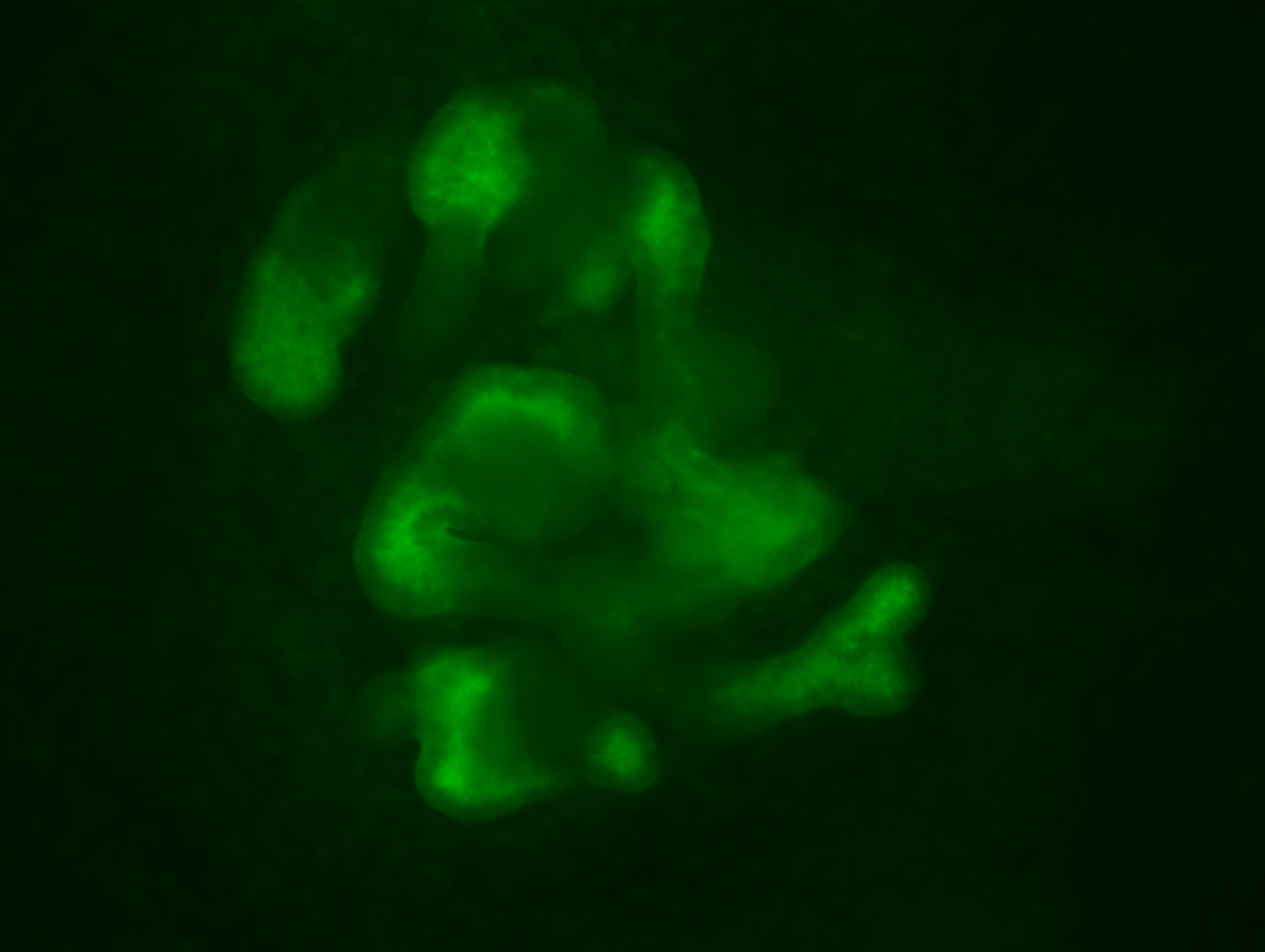

Supplement: Supplementary file 9 — Source Data for Figure 6 [file EMMM-14-e14678-s006.zip › Fig_6._Organoids_Microscopy_uncropped_images/A/LTL.tif]

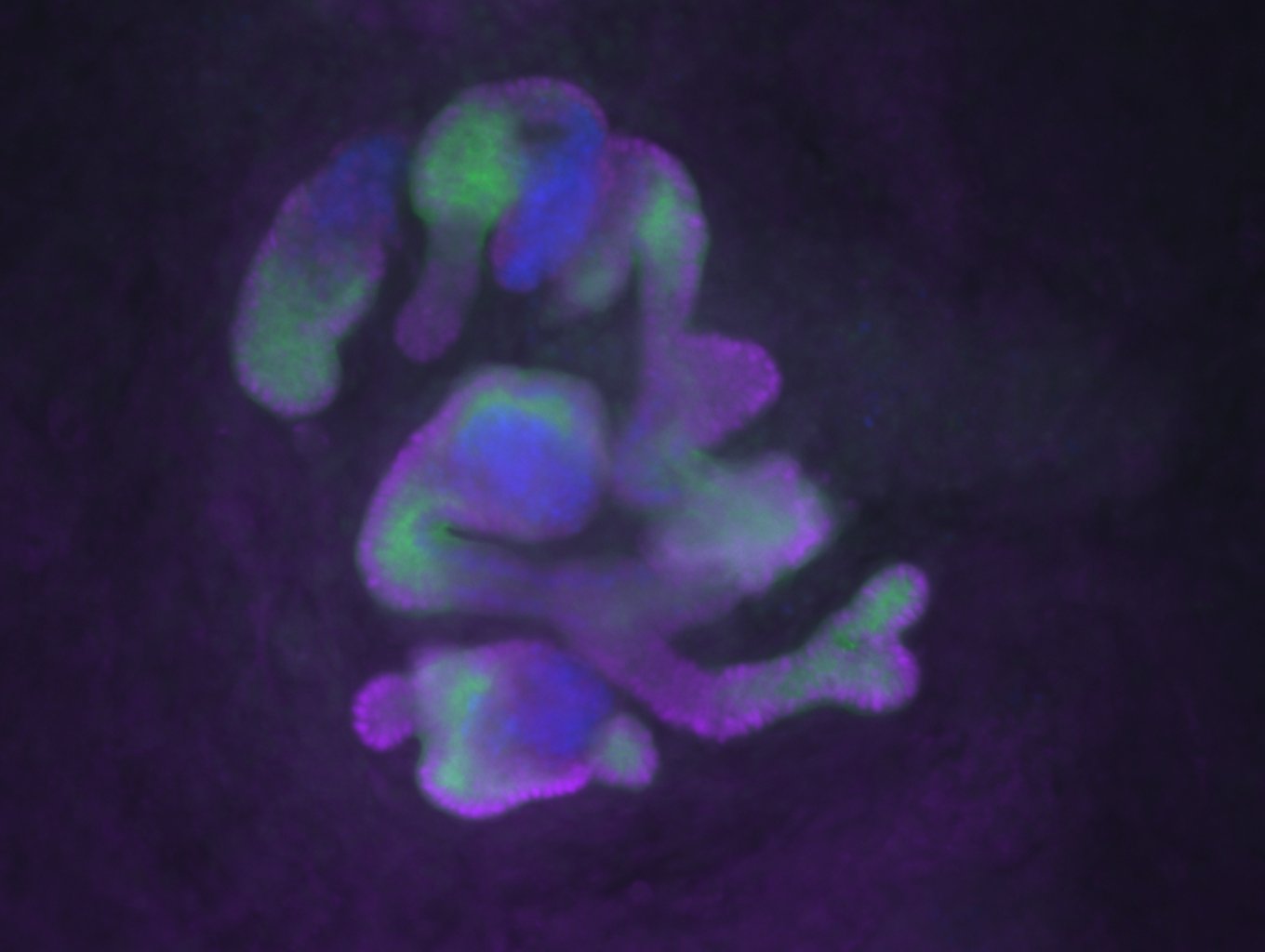

Supplement: Supplementary file 9 — Source Data for Figure 6 [file EMMM-14-e14678-s006.zip › Fig_6._Organoids_Microscopy_uncropped_images/A/LTL_Composite.jpg]

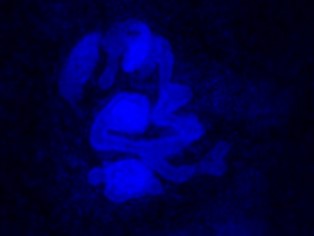

Supplement: Supplementary file 9 — Source Data for Figure 6 [file EMMM-14-e14678-s006.zip › Fig_6._Organoids_Microscopy_uncropped_images/A/LTL_dapi.jpg]

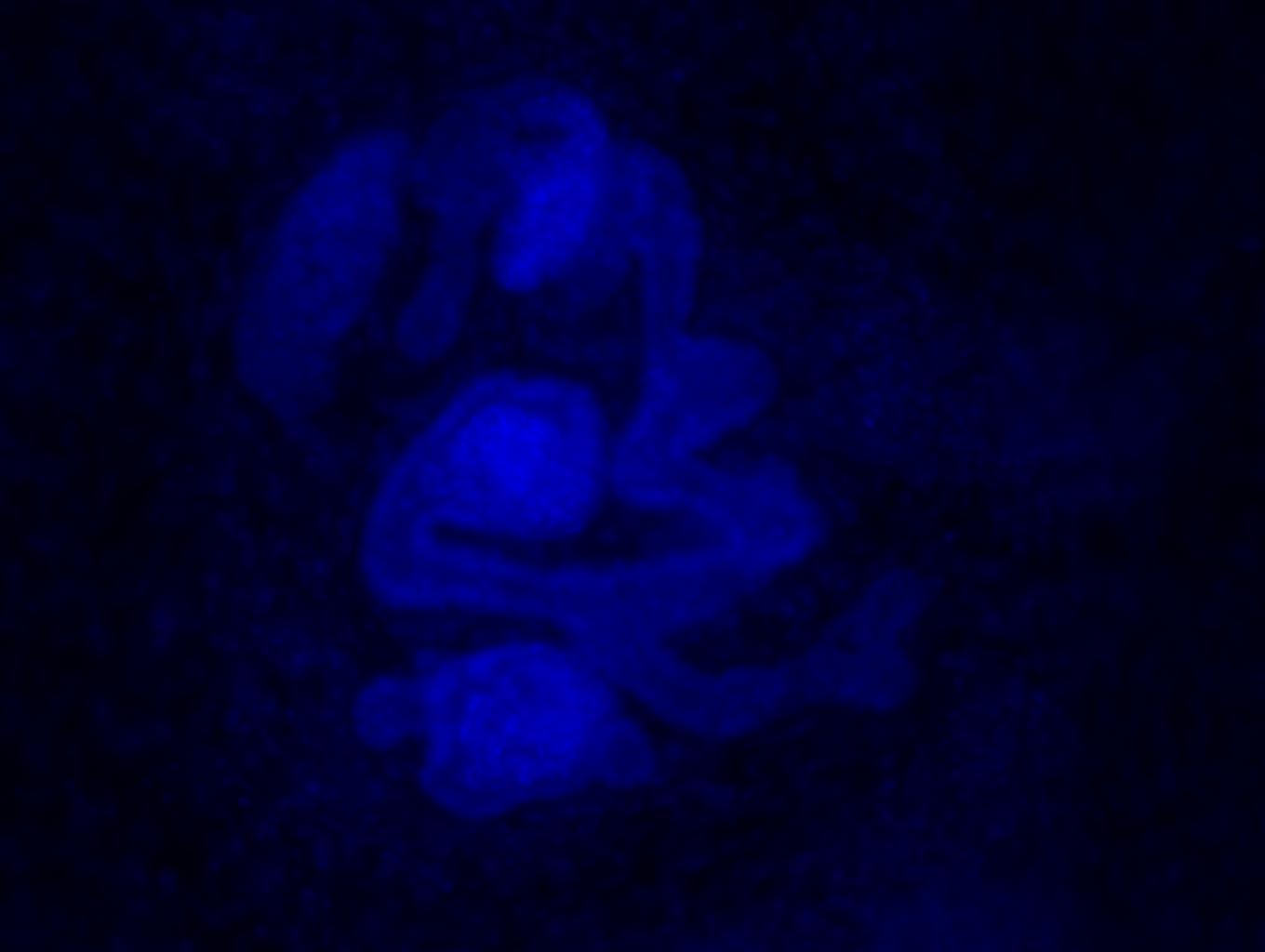

Supplement: Supplementary file 9 — Source Data for Figure 6 [file EMMM-14-e14678-s006.zip › Fig_6._Organoids_Microscopy_uncropped_images/A/LTL_DAPI.tif]

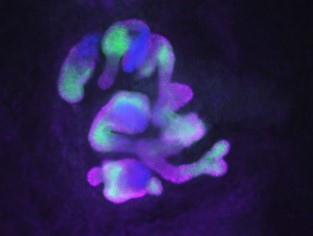

Supplement: Supplementary file 9 — Source Data for Figure 6 [file EMMM-14-e14678-s006.zip › Fig_6._Organoids_Microscopy_uncropped_images/A/LTL_merge.jpg]

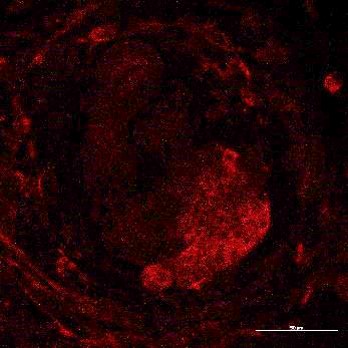

Supplement: Supplementary file 9 — Source Data for Figure 6 [file EMMM-14-e14678-s006.zip › Fig_6._Organoids_Microscopy_uncropped_images/A/NPHS1.jpg]

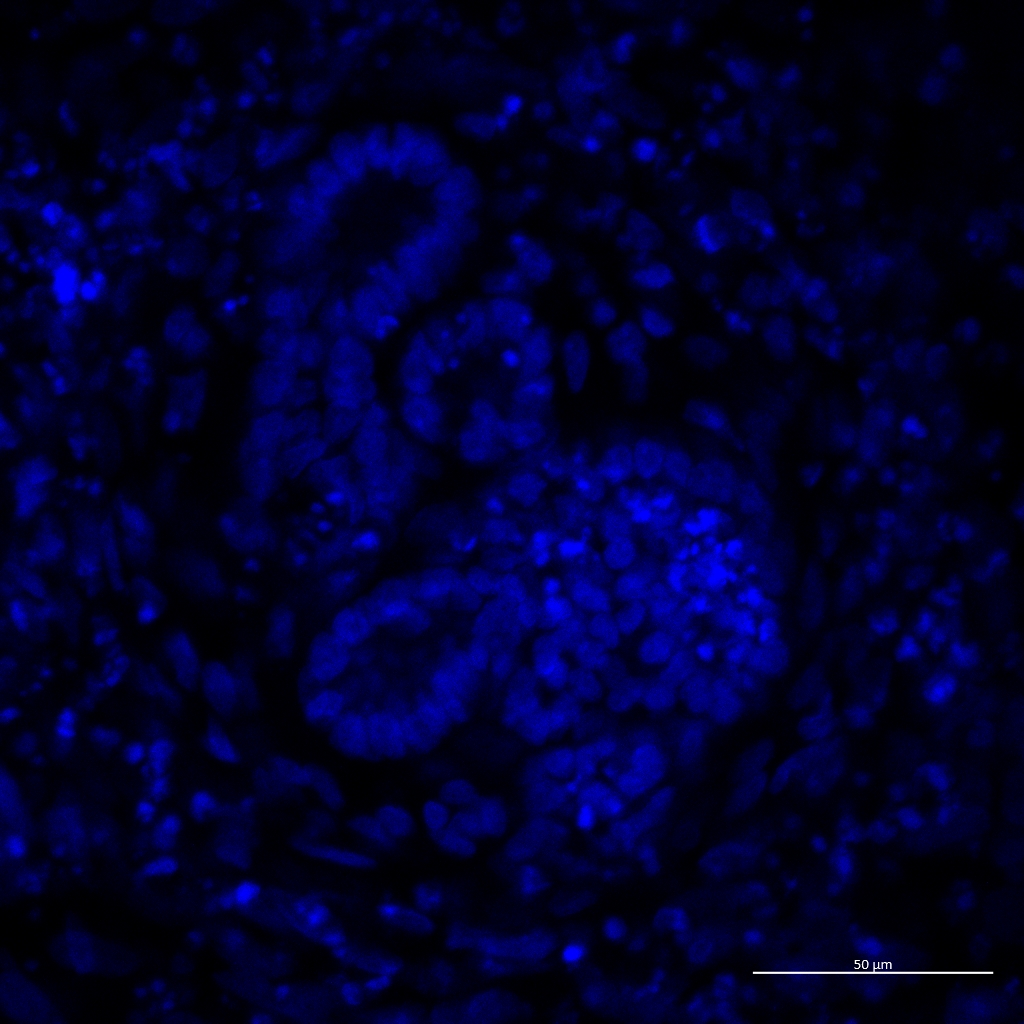

Supplement: Supplementary file 9 — Source Data for Figure 6 [file EMMM-14-e14678-s006.zip › Fig_6._Organoids_Microscopy_uncropped_images/A/NPHS1_dapi.jpg]

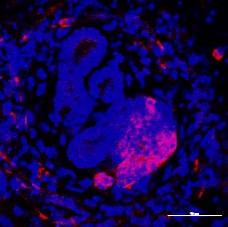

Supplement: Supplementary file 9 — Source Data for Figure 6 [file EMMM-14-e14678-s006.zip › Fig_6._Organoids_Microscopy_uncropped_images/A/NPHS1_merge.jpg]

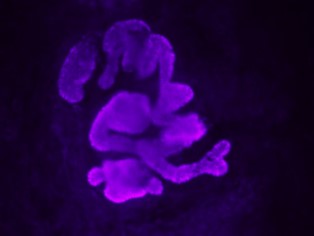

Supplement: Supplementary file 9 — Source Data for Figure 6 [file EMMM-14-e14678-s006.zip › Fig_6._Organoids_Microscopy_uncropped_images/A/PAX8.jpg]

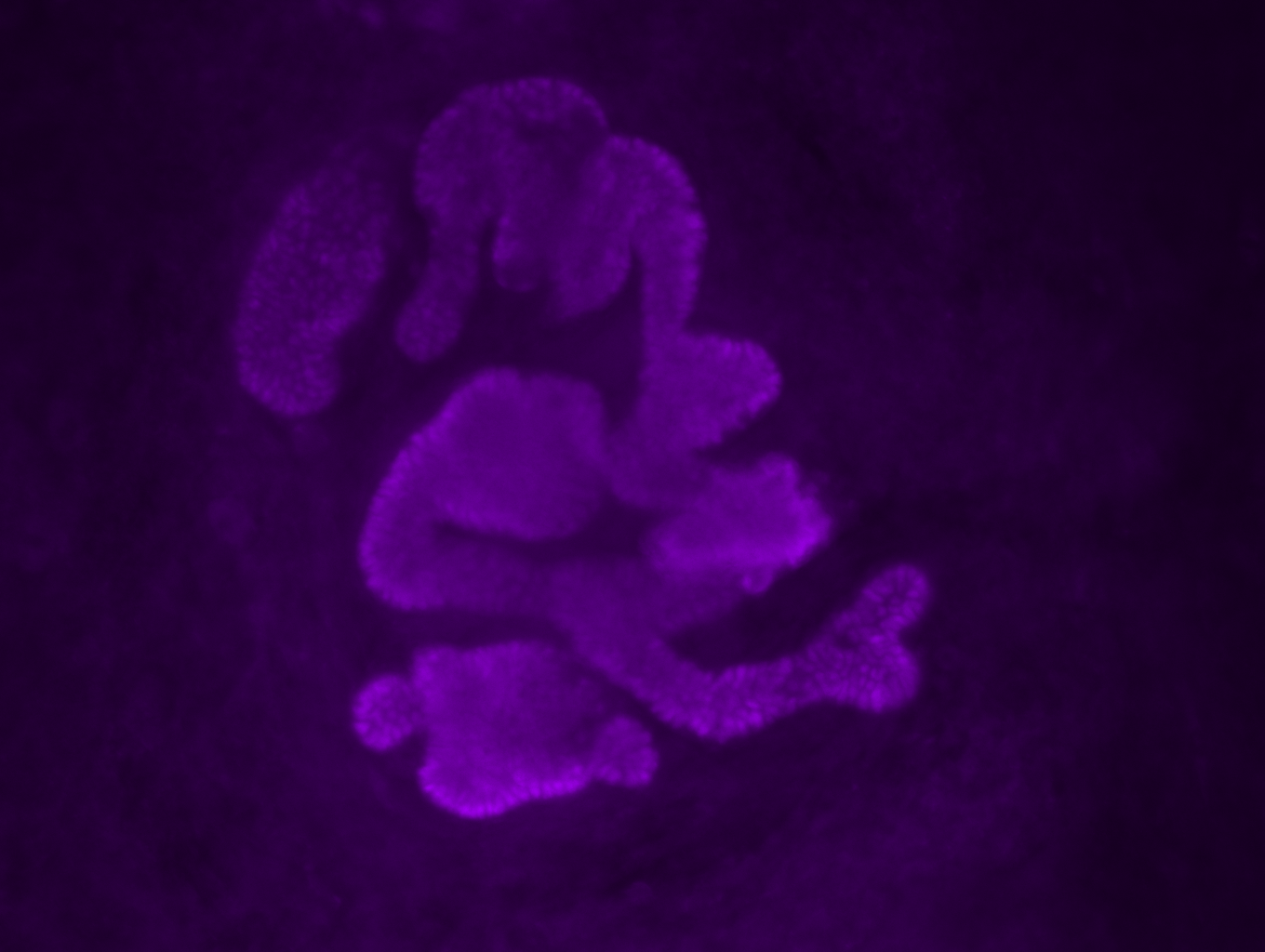

Supplement: Supplementary file 9 — Source Data for Figure 6 [file EMMM-14-e14678-s006.zip › Fig_6._Organoids_Microscopy_uncropped_images/A/PAX8.tif]

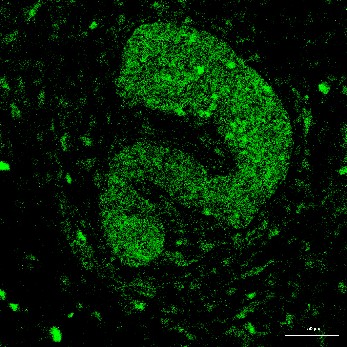

Supplement: Supplementary file 9 — Source Data for Figure 6 [file EMMM-14-e14678-s006.zip › Fig_6._Organoids_Microscopy_uncropped_images/A/SIX3.jpg]

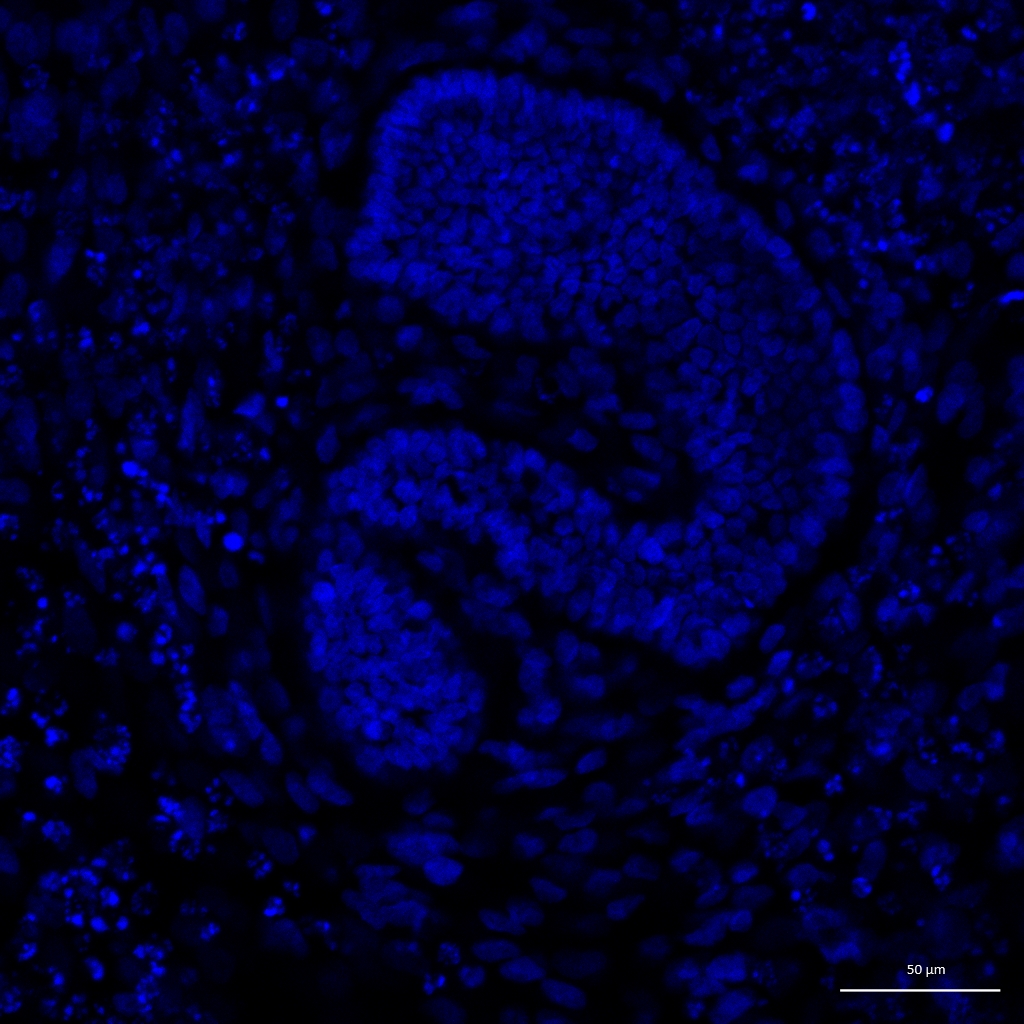

Supplement: Supplementary file 9 — Source Data for Figure 6 [file EMMM-14-e14678-s006.zip › Fig_6._Organoids_Microscopy_uncropped_images/A/SIX3_dapi.jpg]

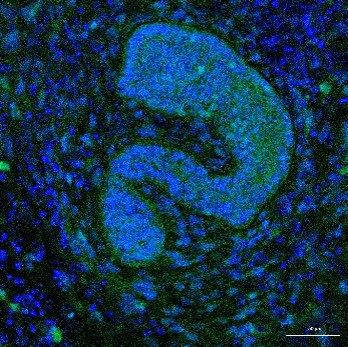

Supplement: Supplementary file 9 — Source Data for Figure 6 [file EMMM-14-e14678-s006.zip › Fig_6._Organoids_Microscopy_uncropped_images/A/SIX3_merge.jpg]

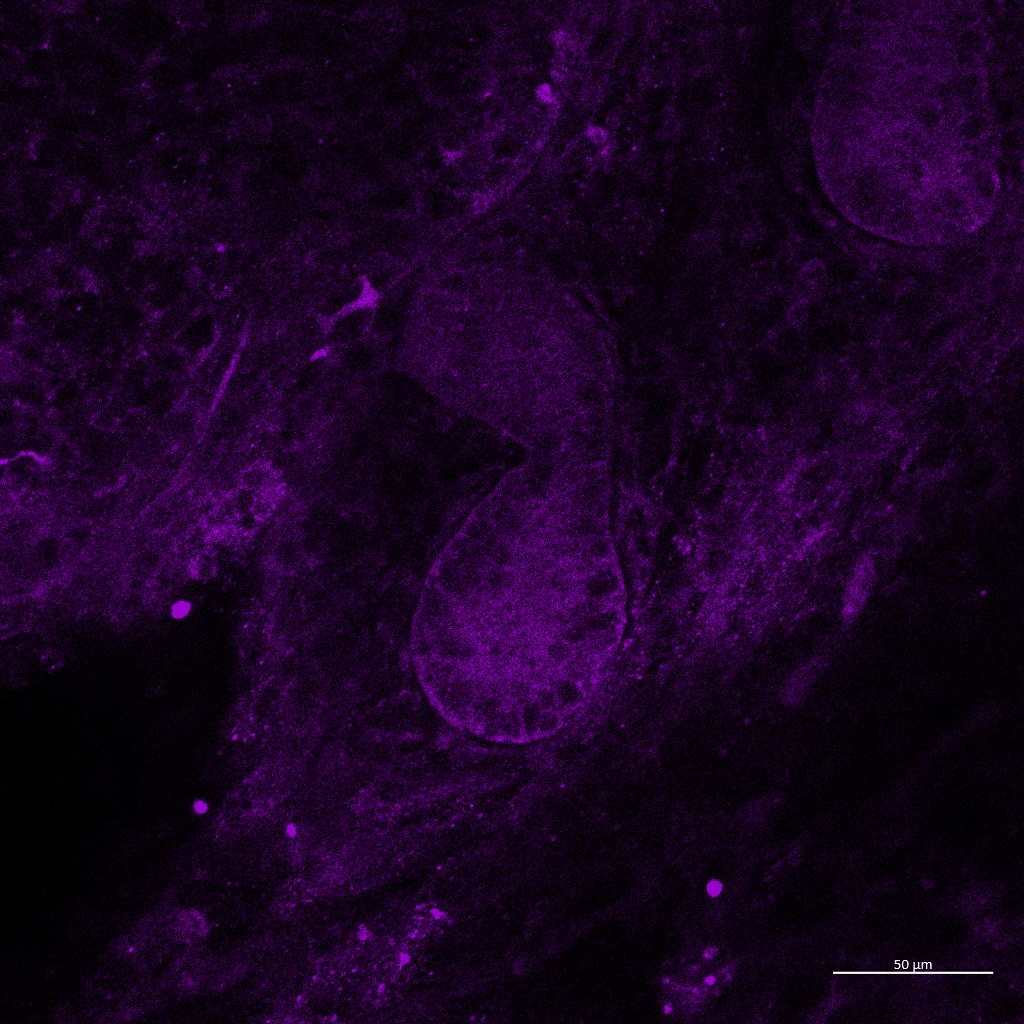

Supplement: Supplementary file 9 — Source Data for Figure 6 [file EMMM-14-e14678-s006.zip › Fig_6._Organoids_Microscopy_uncropped_images/A/WT1(647)_c1.jpg]

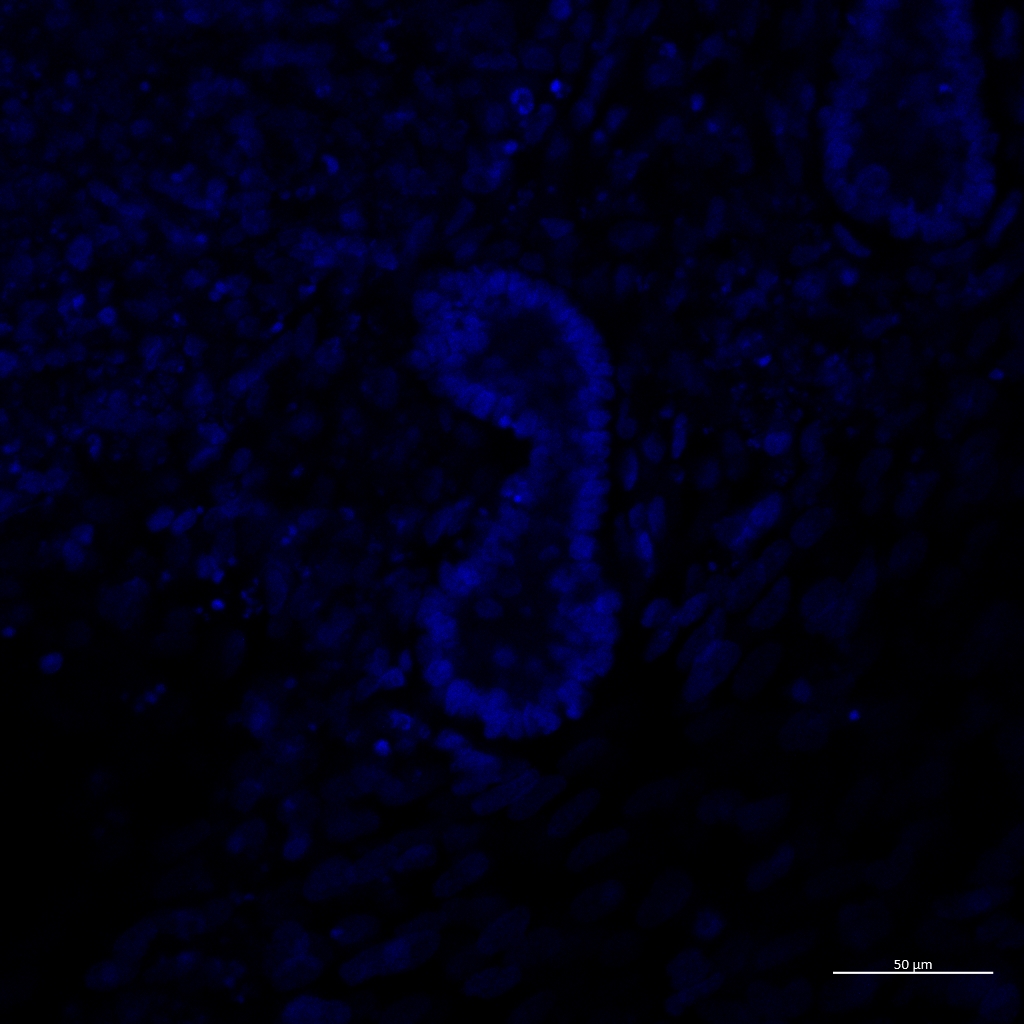

Supplement: Supplementary file 9 — Source Data for Figure 6 [file EMMM-14-e14678-s006.zip › Fig_6._Organoids_Microscopy_uncropped_images/A/WT1(647)_dapi.jpg]

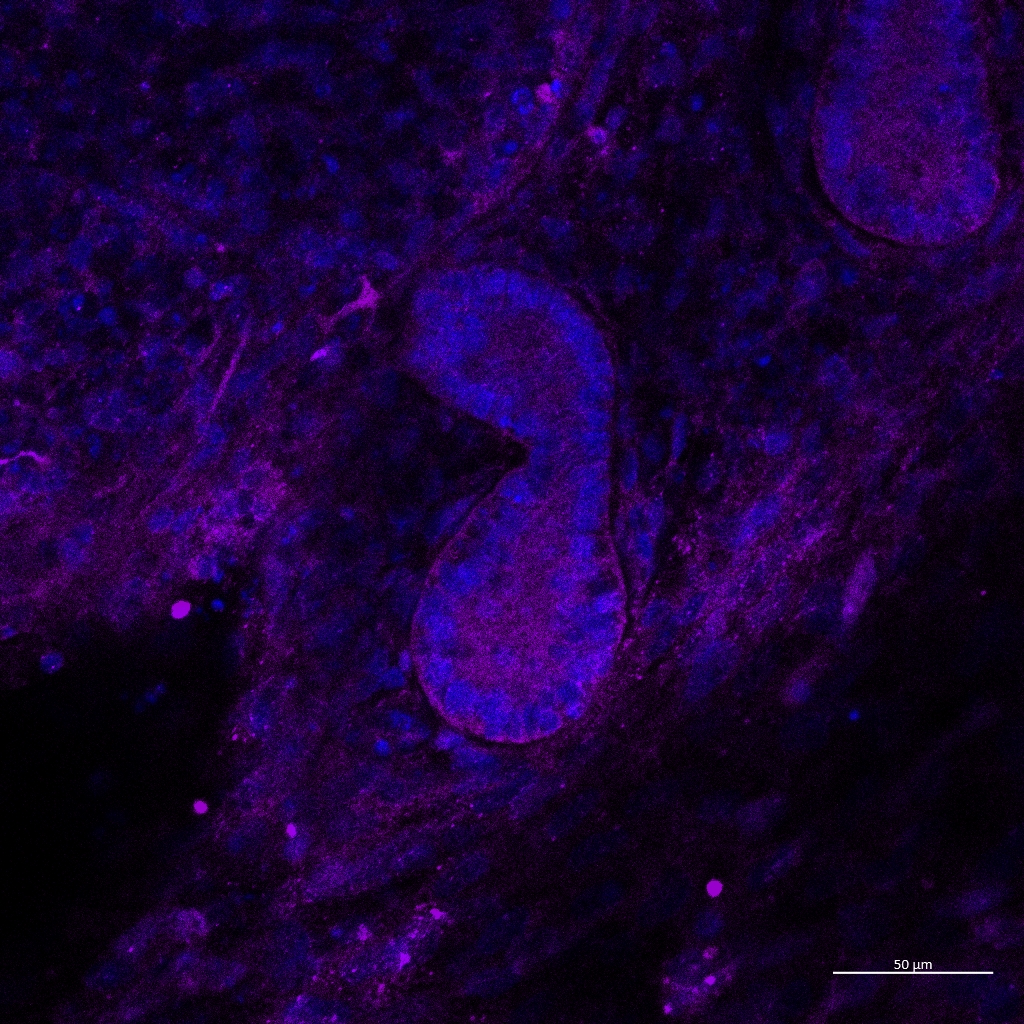

Supplement: Supplementary file 9 — Source Data for Figure 6 [file EMMM-14-e14678-s006.zip › Fig_6._Organoids_Microscopy_uncropped_images/A/WT1(647)_merged.jpg]

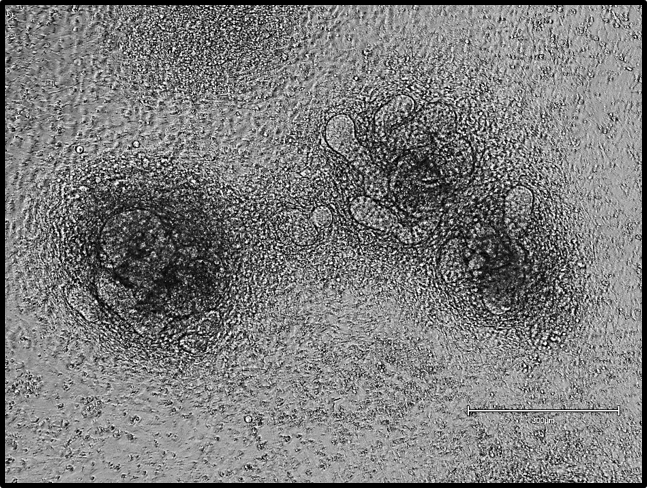

Supplement: Supplementary file 9 — Source Data for Figure 6 [file EMMM-14-e14678-s006.zip › Fig_6._Organoids_Microscopy_uncropped_images/B/OSMI-1_only_1.tif]

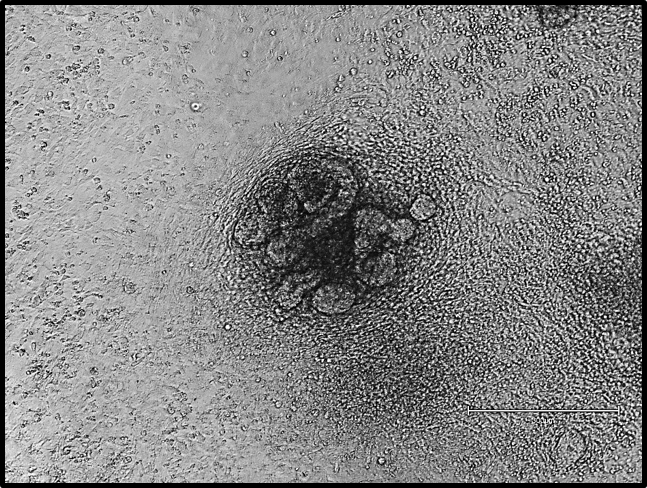

Supplement: Supplementary file 9 — Source Data for Figure 6 [file EMMM-14-e14678-s006.zip › Fig_6._Organoids_Microscopy_uncropped_images/B/OSMI-1_only_2.tif]

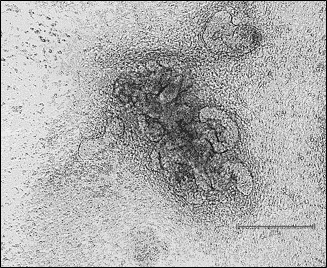

Supplement: Supplementary file 9 — Source Data for Figure 6 [file EMMM-14-e14678-s006.zip › Fig_6._Organoids_Microscopy_uncropped_images/B/OSMI_Stx2_1.tif]

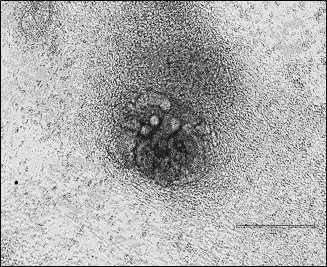

Supplement: Supplementary file 9 — Source Data for Figure 6 [file EMMM-14-e14678-s006.zip › Fig_6._Organoids_Microscopy_uncropped_images/B/OSMI_Stx2_2.tif]

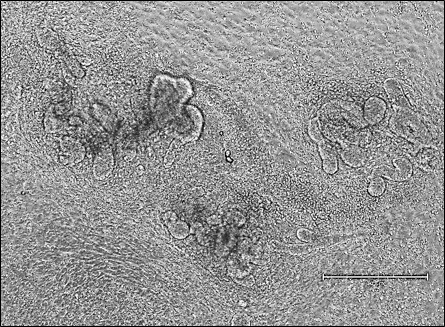

Supplement: Supplementary file 9 — Source Data for Figure 6 [file EMMM-14-e14678-s006.zip › Fig_6._Organoids_Microscopy_uncropped_images/B/Vehicle_only_1.tif]

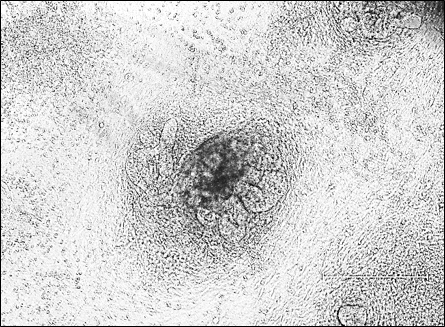

Supplement: Supplementary file 9 — Source Data for Figure 6 [file EMMM-14-e14678-s006.zip › Fig_6._Organoids_Microscopy_uncropped_images/B/Vehicle_only_2.tif]

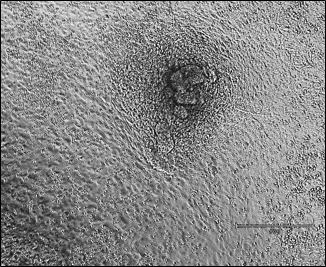

Supplement: Supplementary file 9 — Source Data for Figure 6 [file EMMM-14-e14678-s006.zip › Fig_6._Organoids_Microscopy_uncropped_images/B/Vehicle_Stx2_1.tif]

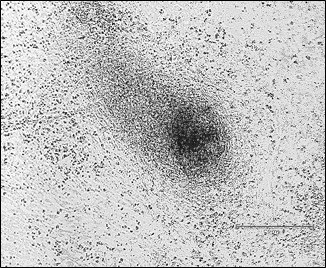

Supplement: Supplementary file 9 — Source Data for Figure 6 [file EMMM-14-e14678-s006.zip › Fig_6._Organoids_Microscopy_uncropped_images/B/Vehicle_Stx2_2.tif]
