# Supplementary material for: Inhibition of O‐GlcNAcylation protects from Shiga toxin‐mediated cell injury and lethality in host
Source: EMBO Mol Med. 2021 Nov 29;14(1):e14678. doi: 10.15252/emmm.202114678 (PMC8749473; doi:10.15252/emmm.202114678)
Supplement: Supplementary file 4 — Source Data for Figure 1 [file EMMM-14-e14678-s005.zip › blots_Figure_1.pptx]

## Slide 1
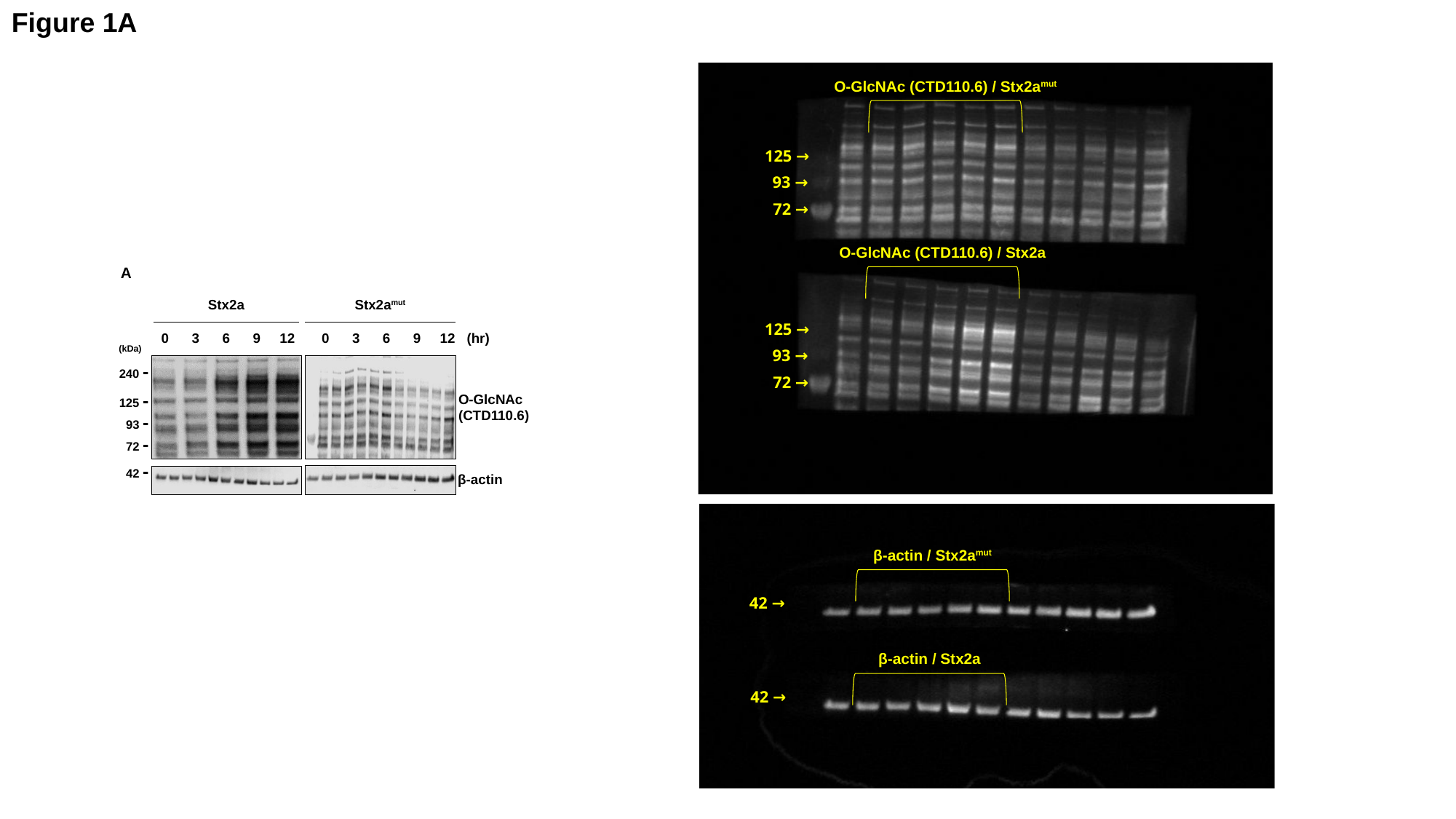

Figure 1A
O-GlcNAc (CTD110.6) / Stx2amut
125 →
93 →
72 →
O-GlcNAc (CTD110.6) / Stx2a
A
Stx2amut
Stx2a
125 →
 0 3 6 9 12 0 3 6 9 12 (hr)
(kDa)
93 →
240 -
125 -
93 -
72 -
42 -
72 →
O-GlcNAc
(CTD110.6)
β-actin
β-actin / Stx2amut
42 →
β-actin / Stx2a
42 →

## Slide 2
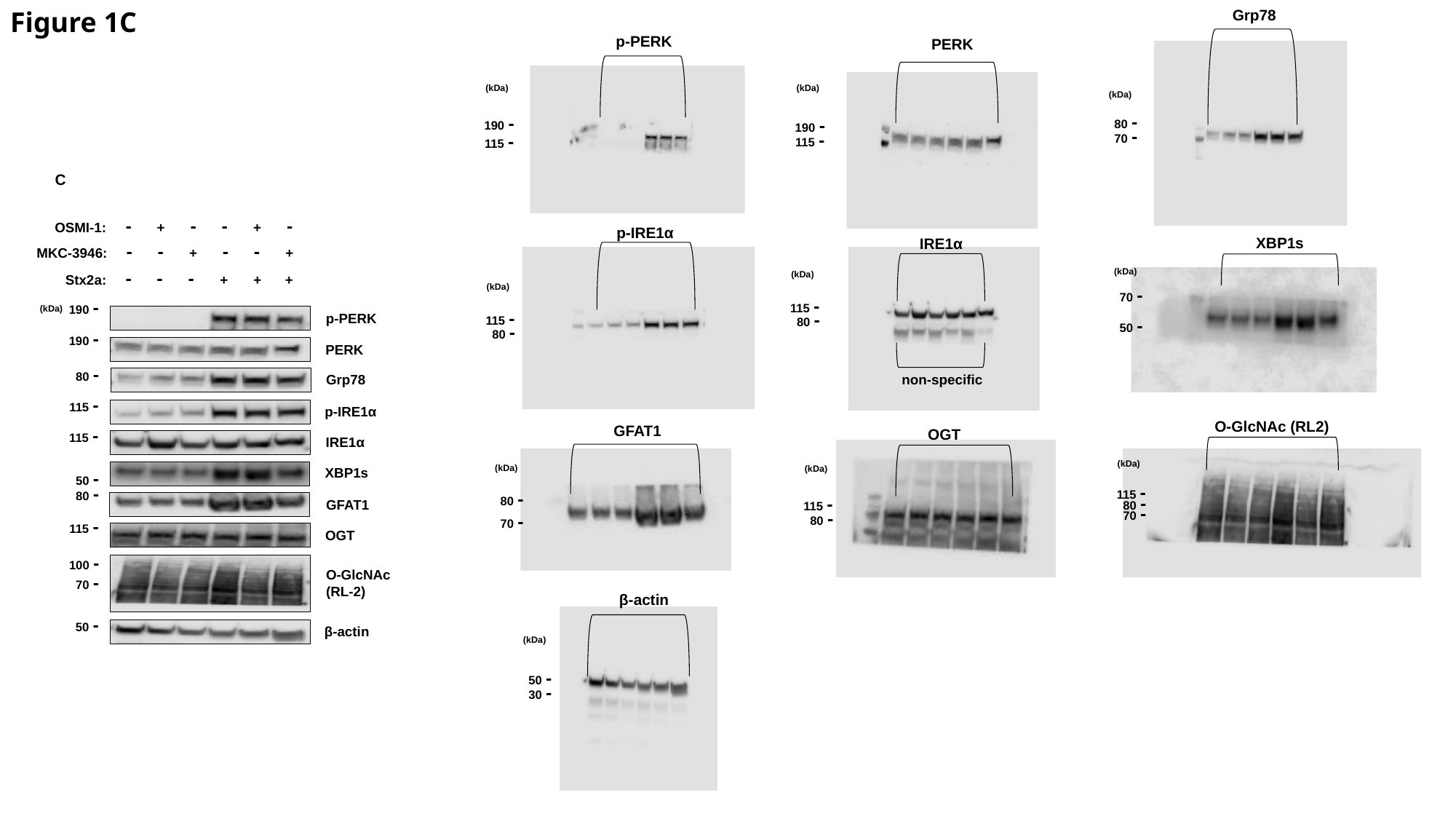

Figure 1C
Grp78
p-PERK
PERK
(kDa)
(kDa)
(kDa)
80 -
190 -
190 -
70 -
115 -
115 -
C
OSMI-1: - + - - + - ­
p-IRE1α
XBP1s
IRE1α
MKC-3946: - - + - - +
(kDa)
Stx2a: - - - + + + ­
(kDa)
(kDa)
70 -
115 -
190 -
(kDa)
115 -
80 -
p-PERK
50 -
80 -
190 -
PERK
80 -
Grp78
non-specific
115 -
p-IRE1α
O-GlcNAc (RL2)
GFAT1
115 -
OGT
IRE1α
(kDa)
(kDa)
(kDa)
XBP1s
50 -
115 -
80 -
80 -
80 -
115 -
GFAT1
70 -
80 -
70 -
115 -
OGT
100 -
O-GlcNAc
(RL-2)
70 -
β-actin
50 -
β-actin
(kDa)
50 -
30 -

## Slide 3
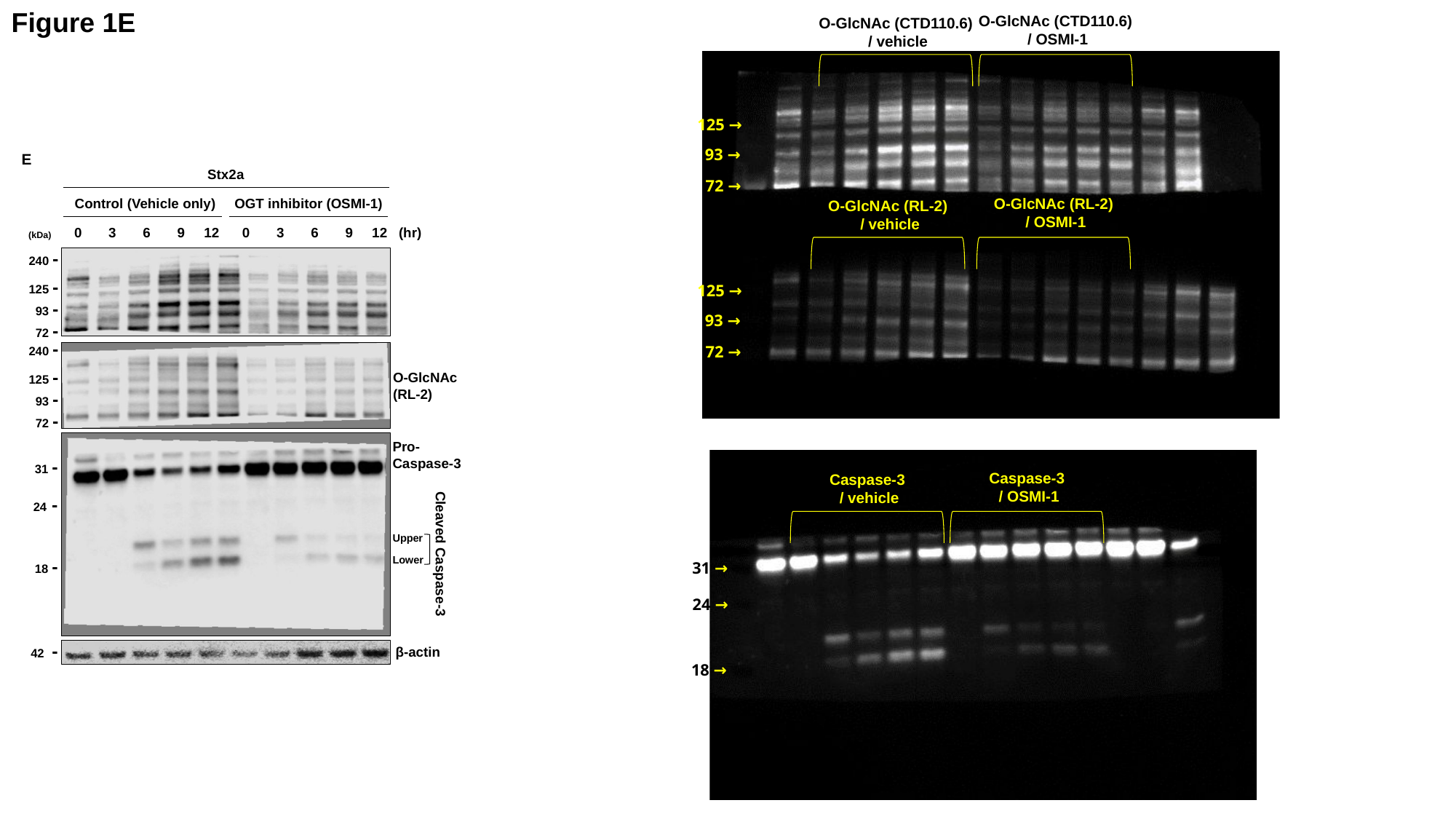

Figure 1E
O-GlcNAc (CTD110.6)
 / OSMI-1
O-GlcNAc (CTD110.6)
 / vehicle
125 →
93 →
E
Stx2a
72 →
O-GlcNAc (RL-2)
 / OSMI-1
Control (Vehicle only)
OGT inhibitor (OSMI-1)
O-GlcNAc (RL-2)
 / vehicle
 0 3 6 9 12 0 3 6 9 12 (hr)
(kDa)
240 -
125 -
93 -
72 -
125 →
93 →
240 -
125 -
93 -
72 -
72 →
O-GlcNAc
(RL-2)
Pro-
Caspase-3
Upper
Lower
31 -
24 -
18 -
Caspase-3
 / OSMI-1
Caspase-3
 / vehicle
Cleaved Caspase-3
31 →
24 →
42 -
β-actin
18 →

## Slide 4
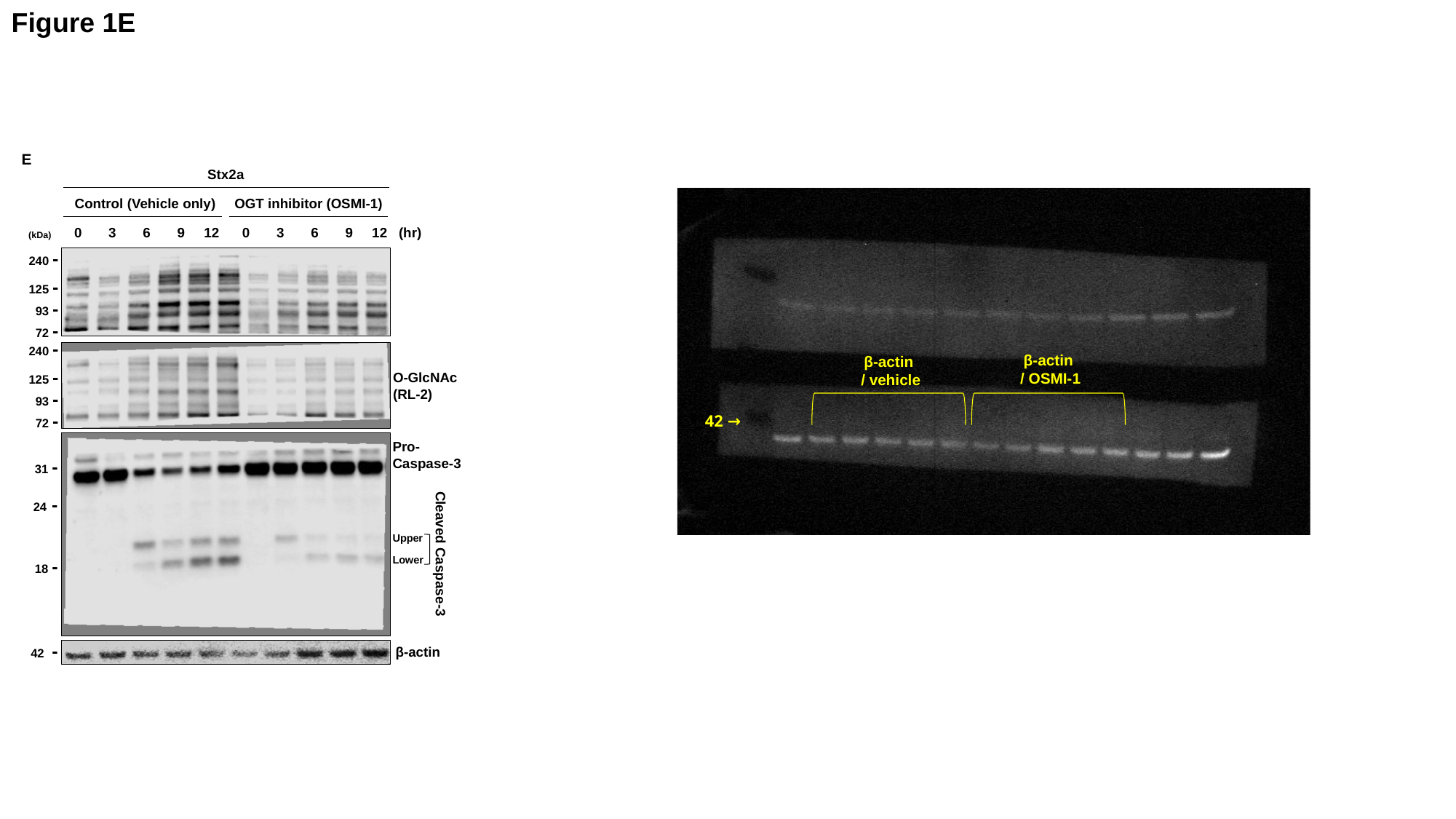

Figure 1E
E
Stx2a
Control (Vehicle only)
OGT inhibitor (OSMI-1)
 0 3 6 9 12 0 3 6 9 12 (hr)
(kDa)
240 -
125 -
93 -
72 -
240 -
125 -
93 -
72 -
β-actin
 / OSMI-1
β-actin
 / vehicle
O-GlcNAc
(RL-2)
42 →
Pro-
Caspase-3
Upper
Lower
31 -
24 -
18 -
Cleaved Caspase-3
42 -
β-actin
