# Supplementary material for: Inhibition of O‐GlcNAcylation protects from Shiga toxin‐mediated cell injury and lethality in host
Source: EMBO Mol Med. 2021 Nov 29;14(1):e14678. doi: 10.15252/emmm.202114678 (PMC8749473; doi:10.15252/emmm.202114678)
Supplement: Supplementary file 6 — Source Data for Figure 3 [file EMMM-14-e14678-s004.zip › blots_Figure_3.pptx]

## Slide 1
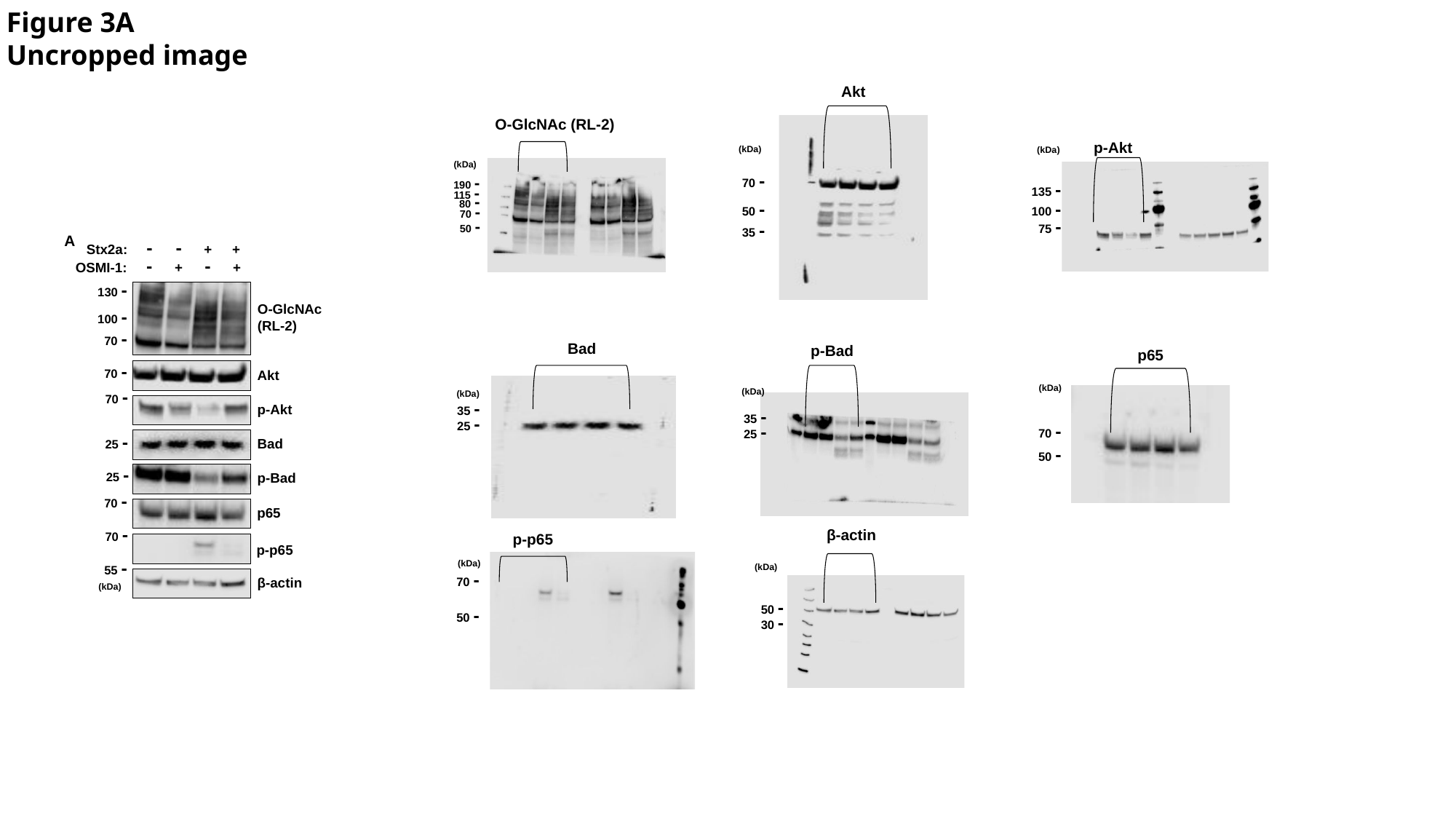

Figure 3A
Uncropped image
Akt
O-GlcNAc (RL-2)
p-Akt
(kDa)
(kDa)
(kDa)
70 -
190 -
135 -
115 -
80 -
100 -
50 -
70 -
75 -
50 -
35 -
A
Stx2a: - - + + ­
OSMI-1: - + - +
130 -
100 -
70 -
O-GlcNAc
(RL-2)
Bad
p-Bad
p65
70 -
Akt
(kDa)
70 -
(kDa)
(kDa)
35 -
p-Akt
35 -
25 -
70 -
25 -
25 -
Bad
50 -
25 -
p-Bad
70 -
p65
70 -
β-actin
p-p65
p-p65
55 -
(kDa)
(kDa)
70 -
β-actin
(kDa)
50 -
50 -
30 -

## Slide 2
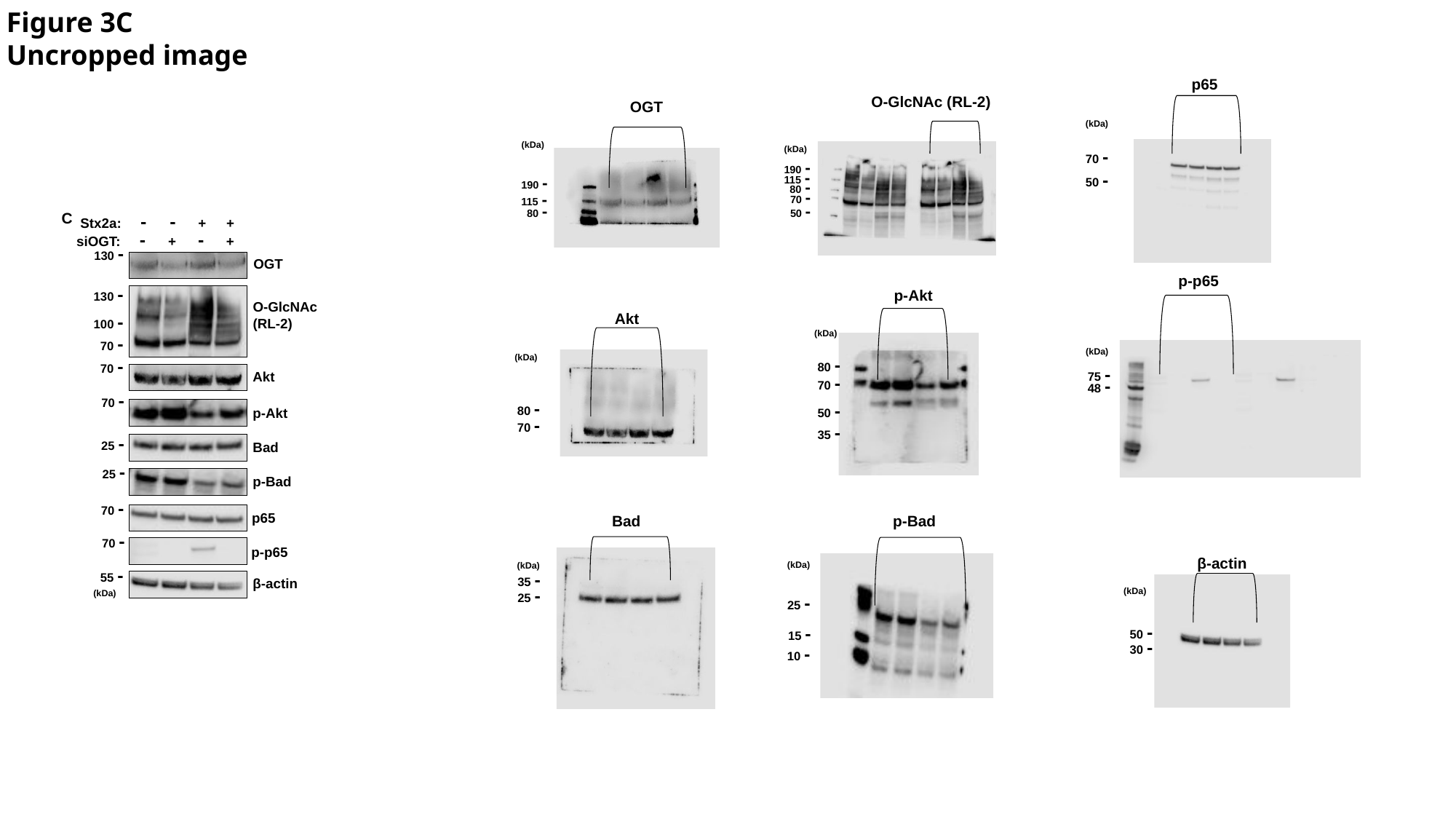

Figure 3C
Uncropped image
p65
O-GlcNAc (RL-2)
OGT
(kDa)
(kDa)
(kDa)
70 -
190 -
115 -
50 -
190 -
80 -
70 -
115 -
80 -
50 -
C
Stx2a: - - + + ­
siOGT: - + - +
130 -
OGT
p-p65
130 -
100 -
70 -
p-Akt
O-GlcNAc
(RL-2)
Akt
(kDa)
(kDa)
(kDa)
80 -
70 -
75 -
Akt
70 -
48 -
70 -
80 -
50 -
p-Akt
70 -
35 -
25 -
Bad
25 -
p-Bad
70 -
p65
Bad
p-Bad
70 -
p-p65
β-actin
(kDa)
(kDa)
55 -
35 -
β-actin
25 -
(kDa)
(kDa)
25 -
50 -
15 -
30 -
10 -

## Slide 3
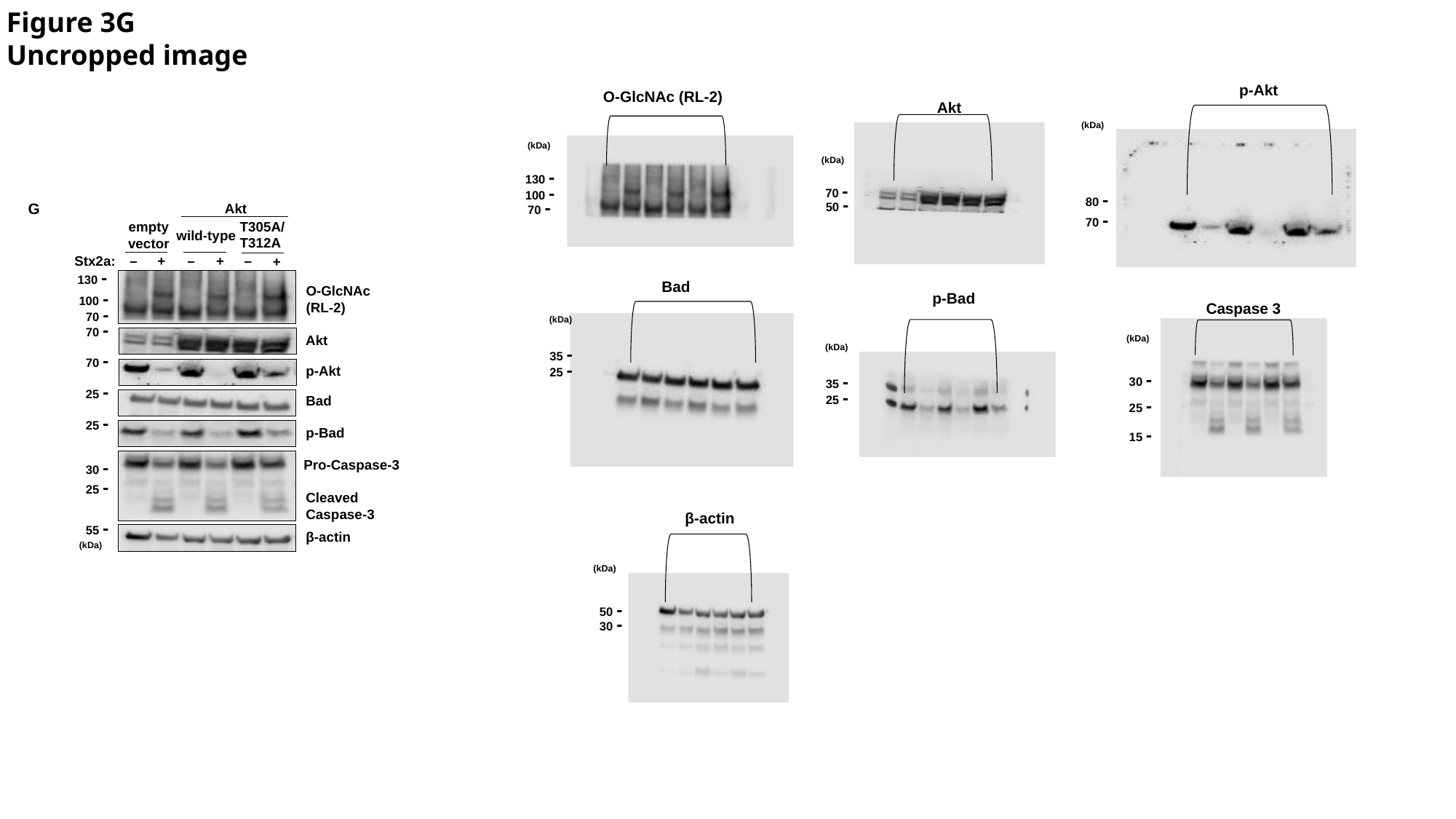

Figure 3G
Uncropped image
p-Akt
O-GlcNAc (RL-2)
Akt
(kDa)
(kDa)
(kDa)
130 -
70 -
100 -
80 -
50 -
70 -
G
 Akt
70 -
T305A/
T312A
empty
vector
wild-type
Stx2a:
–
+
–
+
–
+
130 -
Bad
O-GlcNAc
(RL-2)
100 -
p-Bad
Caspase 3
70 -
(kDa)
70 -
Akt
(kDa)
(kDa)
35 -
70 -
25 -
p-Akt
30 -
35 -
25 -
25 -
Bad
25 -
25 -
15 -
p-Bad
Pro-Caspase-3
30 -
25 -
Cleaved
Caspase-3
β-actin
55 -
β-actin
(kDa)
(kDa)
50 -
30 -
