# Supplementary material for: Inhibition of O‐GlcNAcylation protects from Shiga toxin‐mediated cell injury and lethality in host
Source: EMBO Mol Med. 2021 Nov 29;14(1):e14678. doi: 10.15252/emmm.202114678 (PMC8749473; doi:10.15252/emmm.202114678)
Supplement: Supplementary file 7 — Source Data for Figure 4 [file EMMM-14-e14678-s002.zip › Blots_Figure_4.pptx]

## Slide 1
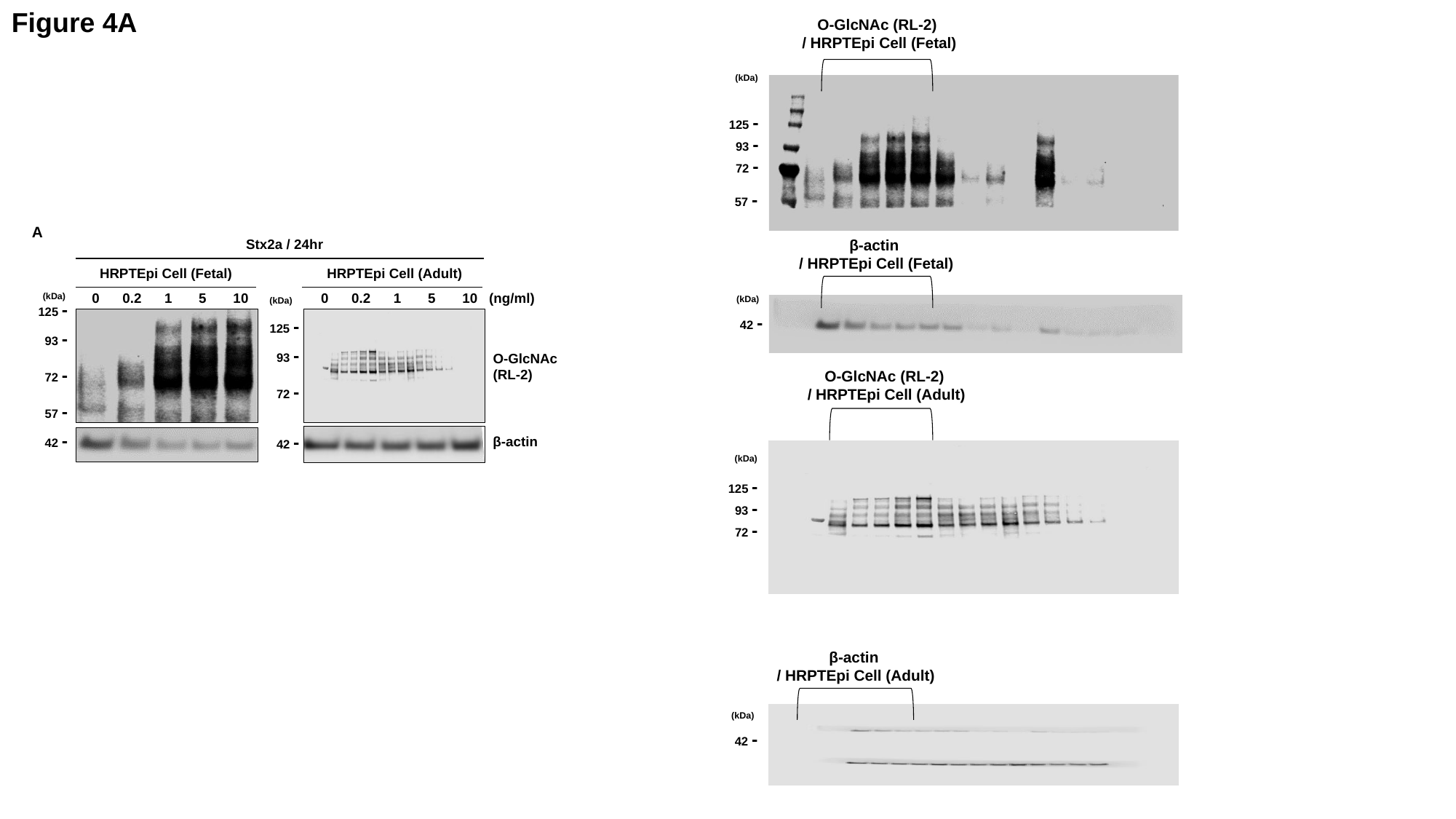

Figure 4A
O-GlcNAc (RL-2)
 / HRPTEpi Cell (Fetal)
(kDa)
125 -
93 -
72 -
57 -
A
Stx2a / 24hr
β-actin
 / HRPTEpi Cell (Fetal)
HRPTEpi Cell (Fetal)
HRPTEpi Cell (Adult)
 0 0.2 1 5 10 0 0.2 1 5 10 (ng/ml)
(kDa)
(kDa)
(kDa)
125 -
93 -
72 -
57 -
42 -
42 -
125 -
93 -
72 -
42 -
O-GlcNAc
(RL-2)
O-GlcNAc (RL-2)
 / HRPTEpi Cell (Adult)
β-actin
(kDa)
125 -
93 -
72 -
β-actin
 / HRPTEpi Cell (Adult)
(kDa)
42 -
