# Supplementary material for: Inhibition of O‐GlcNAcylation protects from Shiga toxin‐mediated cell injury and lethality in host
Source: EMBO Mol Med. 2021 Nov 29;14(1):e14678. doi: 10.15252/emmm.202114678 (PMC8749473; doi:10.15252/emmm.202114678)
Supplement: Supplementary file 11 [file EMMM-14-e14678-s003.pdf]

EMBO Molecular Medicine

Inhibition of O-GlcNAcylation protects from Shiga toxin-mediated cell injury and lethality in host

SUNG-KYUN PARK, Kyung-Soo Lee, Jieun Lee, Pureum Lee, Bong Chan Jeon, Min Yeong Song, Sojung Kwak, Jungwoon Lee, Jun-Seob Kim, Doo-Jin Kim, Ji Hyung Kim, Vernon Tesh, and Moo-Seung Lee
DOI: 10.15252/emmm.202114678

Corresponding authors: SUNG-KYUN PARK (skpark@kribb.re.kr) , Moo-Seung Lee (msl031000@kribb.re.kr)

Review Timeline:

Submission Date:	9th Jun 21
Editorial Decision:	1st Jul 21
Revision Received:	16th Oct 21
Editorial Decision:	5th Nov 21
Revision Received:	8th Nov 21
Accepted:	11th Nov 21

Editors: Lise Roth / Zeljko Durdevic

Transaction Report:

(Note: With the exception of the correction of typographical or spelling errors that could be a source of ambiguity, letters and reports are not edited. Depending on transfer agreements, referee reports obtained elsewhere may or may not be included in this compilation. Referee reports are anonymous unless the Referee chooses to sign their reports.)

1st Jul 2021

Dear Dr. Park,

Thank you for the submission of your manuscript to EMBO Molecular Medicine. We have now received feedback from the three reviewers who agreed to evaluate your manuscript. As you will see from the reports below, the referees acknowledge the interest of the study and are overall supporting publication of your work pending appropriate revisions.

Addressing the reviewers' concerns in full will be necessary for further considering the manuscript in our journal, and acceptance of the manuscript will entail a second round of review. EMBO Molecular Medicine encourages a single round of revision only and therefore, acceptance or rejection of the manuscript will depend on the completeness of your responses included in the next, final version of the manuscript. For this reason, and to save you from any frustrations in the end, I would strongly advise against returning an incomplete revision.

Revised manuscripts should be submitted within three months of a request for revision; they will otherwise be treated as new submissions, except under exceptional circumstances in which a short extension is obtained from the editor.

When submitting your revised manuscript, please carefully review the instructions that follow below. We perform an initial quality control of all revised manuscripts before re-review; failure to include requested items will delay the evaluation of your revision.

We require:

1) A .docx formatted version of the manuscript text (including legends for main figures, EV figures and tables). Please make sure that the changes are highlighted to be clearly visible.

2) Individual production quality figure files as .eps, .tif, .jpg (one file per figure). For guidance, download the 'Figure Guide PDF' (https://www.embopress.org/page/journal/17574684/authorguide#figureformat).

3) A .docx formatted letter INCLUDING the reviewers' reports and your detailed point-by-point responses to their comments. As part of the EMBO Press transparent editorial process, the point-by-point response is part of the Review Process File (RPF), which will be published alongside your paper.

4) A complete author checklist, which you can download from our author guidelines (https://www.embopress.org/page/journal/17574684/authorguide#submissionofrevisions). Please insert information in the checklist that is also reflected in the manuscript. The completed author checklist will also be part of the RPF.

5) It is mandatory to include a 'Data Availability' section after the Materials and Methods. Before submitting your revision, primary datasets produced in this study need to be deposited in an appropriate public database, and the accession numbers and database listed under 'Data Availability'. Please remember to provide a reviewer password if the datasets are not yet public (see https://www.embopress.org/page/journal/17574684/authorguide#dataavailability).

In case you have no data that requires deposition in a public database, please state so in this section. Note that the Data Availability Section is restricted to new primary data that are part of this study.

6) For data quantification: please specify the name of the statistical test used to generate error bars and P values, the number (n) of independent experiments (specify technical or biological replicates) underlying each data point and the test used to calculate p-values in each figure legend. The figure legends should contain a basic description of n, P and the test applied. Graphs must include a description of the bars and the error bars (s.d., s.e.m.).

7) We would also encourage you to include the source data for figure panels that show essential data. Numerical data should be provided as individual .xls or .csv files (including a tab describing the data). For blots or microscopy, uncropped images should be submitted (using a zip archive if multiple images need to be supplied for one panel). Additional information on source data and instruction on how to label the files are available at
.

8) Our journal encourages inclusion of *data citations in the reference list* to directly cite datasets that were re-used and obtained from public databases. Data citations in the article text are distinct from normal bibliographical citations and should directly link to the database records from which the data can be accessed. In the main text, data citations are formatted as follows: "Data ref: Smith et al, 2001" or "Data ref: NCBI Sequence Read Archive PRJNA342805, 2017". In the Reference list, data citations must be labeled with "[DATASET]". A data reference must provide the database name, accession number/identifiers and a resolvable link to the landing page from which the data can be accessed at the end of the reference.

Further instructions are available at .

9) We replaced Supplementary Information with Expanded View (EV) Figures and Tables that are collapsible/expandable online. A maximum of 5 EV Figures can be typeset. EV Figures should be cited as 'Figure EV1, Figure EV2" etc... in the text and their respective legends should be included in the main text after the legends of regular figures.
- For the figures that you do NOT wish to display as Expanded View figures, they should be bundled together with their legends in a single PDF file called *Appendix*, which should start with a short Table of Content. Appendix figures should be referred to in the main text as: "Appendix Figure S1, Appendix Figure S2" etc.
- Additional Tables/Datasets should be labeled and referred to as Table EV1, Dataset EV1, etc. Legends have to be provided in a separate tab in case of .xls files. Alternatively, the legend can be supplied as a separate text file (README) and zipped together with the Table/Dataset file.

See detailed instructions here:
.

10) The paper explained: EMBO Molecular Medicine articles are accompanied by a summary of the articles to emphasize the major findings in the paper and their medical implications for the non-specialist reader. Please provide a draft summary of your article highlighting
- the medical issue you are addressing,
- the results obtained and
- their clinical impact.

This may be edited to ensure that readers understand the significance and context of the research. Please refer to any of our published articles for an example.

11) For more information: There is space at the end of each article to list relevant web links for further consultation by our readers. Could you identify some relevant ones and provide such information as well? Some examples are patient associations, relevant databases, OMIM/proteins/genes links, author's websites, etc...

12) Every published paper now includes a 'Synopsis' to further enhance discoverability. Synopses are displayed on the journal webpage and are freely accessible to all readers. They include a short stand first (maximum of 300 characters, including space) as well as 2-5 one-sentences bullet points that summarizes the paper. Please write the bullet points to summarize the key NEW findings. They should be designed to be complementary to the abstract - i.e. not repeat the same text. We encourage inclusion of key acronyms and quantitative information (maximum of 30 words / bullet point). Please use the passive voice. Please attach these in a separate file or send them by email, we will incorporate them accordingly.

Please also suggest a striking image or visual abstract to illustrate your article as a PNG file 550 px wide x 300-600 px high.

13) As part of the EMBO Publications transparent editorial process initiative (see our Editorial at http://embomolmed.embopress.org/content/2/9/329), EMBO Molecular Medicine will publish online a Review Process File (RPF) to accompany accepted manuscripts.
In the event of acceptance, this file will be published in conjunction with your paper and will include the anonymous referee reports, your point-by-point response and all pertinent correspondence relating to the manuscript. Let us know whether you agree with the publication of the RPF and as here, if you want to remove or not any figures from it prior to publication.
Please note that the Authors checklist will be published at the end of the RPF.

EMBO Molecular Medicine has a "scooping protection" policy, whereby similar findings that are published by others during review or revision are not a criterion for rejection. Should you decide to submit a revised version, I do ask that you get in touch after three months if you have not completed it, to update us on the status.

I look forward to receiving your revised manuscript.

Yours sincerely,

Lise Roth

Lise Roth, PhD
Editor
EMBO Molecular Medicine

***** Reviewer's comments *****

Referee #1 (Remarks for Author):

This manuscript reports the findings that Shiga toxins can induce upregulation of O-GlcNAcylation, a form of post-translational modification of proteins on serine/threonine residues. Inhibition of O-GlcNAcylation reduced toxin-induced apoptosis and release of pro-inflammatory cytokines. The author proposed that this effect is linked with Akt and NF-γB pathways due to changes in O-GlcNAcylation and phosphorylation in Akt and p65 proteins. The authors utilized a small molecule inhibitor of O-GlcNAcylation transferase, OSMI-1, and validated that inhibition of O-GlcNAcylation is protective against Shiga toxins on THP-1 cells, primary human renal proximal tubular epithelial cells, 3D human kidney spheroids, and iPSC-derived human renal organoids. Finally, the authors showed that OSMI-1 injection reduced the toxicity of Shiga toxin in mice in vivo.
The study is well organized and focused. Although the mechanistic link to Akt and NF-γB remains relatively weak, the findings provide new insight to Stx pathogenesis and potential new therapeutic approaches.
Major point: Fig1 and 2, particularly Fig. 2 need additional controls to examine whether apoptosis and pro-inflammatory cytokines are affected by OSMI-1, independent of Stx2a treatment, for instance, under the ER-stress conditions induced by other types of stimuli? In addition, ER stress markers need to be validated in the immunoblots.
Minor points:
1. Citation needed for "induction page 1, second line from the bottom".
2. Page 4: line 3 from the bottom: "small G protein" to "small GTPase".
3. the label of "Stx2a-/-" should be changed to clearly label it as a mutant, as "Stx2a-/-" could be confusing with knockout.
4. Page 6, last two lines change to "suggest" instead of "indicate", as there is no evidence on bacterial infection, only data on toxins.
5. Page 12, line 4: should be "3D"?
6. What is the rationale of choosing 132.5 ng/Kg dose?

Referee #2 (Comments on Novelty/Model System for Author):

Overall this manuscript describes a unique finding that occurs in cells after exposure to Shiga toxins that appears to contribute to the cellular response to the toxin. The study is fairly complete (with one additional control desired). They even took the inhibitor into an animal model and appeared to get positive results, though the statistics were not reported.

Referee #2 (Remarks for Author):

The manuscript demonstrates that undifferentiated THP-1 cells, differentiated THP-1 cells, and HRPTEpi cells show elevated O-GlcNAcylation in response to treatment with Stx2a, but not to an Stx2a enzymatic mutant. Intriguingly, the enzyme (OGT) responsible for O-GlcNAcylation was not elevated upon Stx2a exposure. Rather, upstream enzyme GFAT1 was increased. Therefore, the authors propose that there is more substrate for OGT available, which leads to the increased O-GlcNAcylation. The elevated O-GlcNAcylation could be reduced with OSMI-1 treatment or inhibitory RNA. The increase in O-GlcNAcylation was correlated with retrograde transport of Stx2a and with the ER stress response. Additionally, the authors conclude that the increased O-GlcNAcylation is what leads to the reduced p-Akt and increased p-p65 after Stx2a treatment, but that link was not definitively shown. Finally, the OGT inhibitor was tested in spheroid and organoid models and showed the capacity to reduce elevations in cytokine responses after Stx2a exposure. There was a lack of controls with OSMI-1 alone overall; this lack was surprising since the authors spent time in the discussion describing the different affects that O-GlcNAcylation may have in different cell types. A control of OSMI-1 treated THP-1 cells at least is appropriate, and could be reported in the text if there are no differences.
The animal data was suggestive, but no statistics were shown for those data, so the reader must assume the survival curves and clinical parameters were not different. It will be interesting in the future to see if OSMI-1 or another O-GlcNAcylation inhibitor could be used as a treatment in an animal model rather than as a preventative therapy.

Additional concerns
1. The authors state that severe clinical symptoms of mice were improved (page 5). No stats were done, so this is not clear.
2. Page 6 - both XBP1 and GFAT were elevated-presumably linked, but not shown by this study, rather, based on Wang reference. Also, since no bacteria were used, and since EHEC express other factors, including those injected by a type III secretion system, the hypothesis should be about toxin, not bacteria. Speculation can be in the discussion.
3. Page 7. OSMI-1 reduced the elevated O-GlcNAcylation, but did not "suppress" it.
4. Fig EV2A - the OSMI-1 treatment is significant, but modest.
5. Not clear why Stx1a was suddenly brought in? It is not a completely different toxin type-it traffics similarly and has the same mechanism of action as Stx2a.
6. Figure 2 - what are the no toxin or OSMI alone control levels?
7. Figure 3B - do statistical comparisons for OSMI-1 treatment with and without Stx2a-should help distinguish if the treatment

restores the levels of the proteins-it looks like it for p65 and perhaps p-Akt (though it does not look like it looking at the blots themselves??), but not p-Bad
8. RNA-seq, no OSMI-1-alone control - what does OSMI do to cells on its own.
9. P9 how do we know the O-GlcNAcylation leads to the phosphorylation changes? Figure 3A-OSMI1 treatment alone does not lead to an increase in p-p65, for example. Does the increase in p-p65 and decrease in p-Bad occur after the O-GlcNAcylation starts? Or do you see those changes as early as you see the increase in O-GlcNAcylation - 3-6 hours based on Figure 1. Certainly the change in Caspase is observed by 3 hours (Figure 1D).
10. P10 not clear that these results show that the O-GlcNac inhibition works through Akt and p65
11. The authors should consider include the following paper in the discussion: Parello et al. 2015. Shiga toxin2-induced endoplasmic reticulum stress is minimized ... Toxins. 7

Other comments.
1. More references are needed in a few places. For example, second sentence of the introduction and at the end of the 7th line of the results.
2. The introduction, though informative, is rather long and could be shortened without losing relevant background. Additionally, the reader is left wondering why the authors chose to look at this specific post-translational modification?
3. Not clear why the Stx2a"-/-"designation is used for the holotoxoid? There are 3 mutations. Just Stx2a- would be sufficient, or Stx2a-/-/- if the desire is to indicate the # of mutations.
4. Page 6. Figure 1 panel A shows levels of O-GlcNAcylation, not ER stress response.
5. Figure 1 figure legend (page 34)-should describe what is shown from top to bottom, not bottom to top-- O-GlcNAcylation should come before phosphorylation. Panel A before Panel C and so on.
6. Figure 1C it would be nice to see an earlier time point, say 3 hours, for the XBP1 and GFAT1-since presumably an increase in those factors would precede the increase in O-GlcNAcylation that is detected by 6 hours.
7. Figure EV2A - Panels A and B description - panels show 4 time points, not just 9 h.

Referee #3 (Remarks for Author):

This is an interesting paper that reports that inhibiting the OGT enzyme responsible for glycosylating proteins on Ser/Thr residues with GlcNAc can reduce cell injury and mouse lethality due to Shiga toxin. The data could eventually inform therapeutic approaches to use OGT inhibition to reduce Shiga toxin pathology in the context of enterohemorrhagic E. coli infections. The following issues should be corrected and/or clarified:

Abstract - change 'generated' to 'produced'; change 'fine regulation' to 'inhibition'

Results - need a reference for the statement that the Stx2a mutant triggers a limited ER stress response

Results, first paragraph - it is not UPD-GlcNAc production that is necessarily upregulated. This is an awkward statement. Presumably what the others mean at this point in the paper is that the Fig. 1 phenotype could be due to increased OGT activity or reduced OGA activity

Results - need a reference for OSMI-1 activity the first time it is described

Results - need a reference for Retro-2 activity the first time it is described

Figure 1D - describe why both CTD110.6 and RL-2 antibodies were used

Figure 1D - quantify the GlcNAc signals as done in Figs. 1A-B

Figure 3A - the stated difference in GlcNAc signals is not apparent from the blot provided as representative data

Figure 3E - why are p65 data not shown?

Figure 7B - statistical analysis is needed

Figure 7C - statistical analysis is needed

Sep 29, 2021

EMBO Molecular Medicine

Dear Editor:

We have revised our manuscript (EMM-14678), "**Inhibition of O-GlcNAcylation protects from Shiga toxin-mediated cell injury and lethality in host**".

Below, we summarized our revisions according to the reviewers' comments in a point-by-point way. Please note that the figure numbers and panel alphabets in this letter refer to the revised version of our manuscript. The changed or newly-added portions have been highlighted by shading with yellow color in the revised manuscript.

==

Answers and revisions to the comments of reviewer #1:

Reviewer's Remarks for Author:

This manuscript reports the findings that Shiga toxins can induce upregulation of O-GlcNAcylation, a form of post-translational modification of proteins on serine/threonine residues. Inhibition of O-GlcNAcylation reduced toxin-induced apoptosis and release of pro-inflammatory cytokines. The author proposed that this effect is linked with Akt and NF-γB pathways due to changes in O-GlcNAcylation and phosphorylation in Akt and p65 proteins. The authors utilized a small molecule inhibitor of O-GlcNAcylation transferase, OSMI-1, and validated that inhibition of O-GlcNAcylation is protective against Shiga toxins on THP-1 cells, primary human renal proximal tubular epithelial cells, 3D human kidney spheroids, and iPSC-derived human renal organoids. Finally, the authors showed that OSMI-1 injection reduced the toxicity of Shiga toxin in mice in vivo.

The study is well organized and focused. Although the mechanistic link to Akt and NF-γB remains relatively weak, the findings provide new insight to Stx pathogenesis and potential new therapeutic approaches.

***Response**: To supplement the mechanistic link, as the reviewer considered, we performed the additional experiments. Once Akt with mutation for O-GlcNAcylation sites, Thr305 and Thr312, was overexpressed, cells were more resistant to apoptosis under Stx exposure than those with wild-type form due to the lack of induction for phosphorylation at Thr308 (Fig 3G, H and I). We believe that this result more directly proved that the apoptotic effect of Akt through phosphorylation at Thr308 is regulated depending on O-GlcNAcylation status at its adjacent sites, Thr305 and Thr312.*

Major point:

1. Fig1 and 2, particularly Fig. 2 need additional controls to examine whether apoptosis and pro-inflammatory cytokines are affected by OSMI-1, independent of Stx2a treatment, for instance, under the ER-stress conditions induced by other types of stimuli?

***Response**: Basically, the treatment of OSMI-1 alone does not affect apoptotic or pro-inflammatory responses. Because cells were pre-treated with OSMI-1 for 12h before Stx exposure, no increase of either cleaved caspase-3 (Fig 1E and G) or fluorescent cells of the TUNEL assay (Fig 1H) at 0 h time-point under Stx2a plus OSMI-1 condition reflected that the treatment of OSMI-1 alone has no significant effect on inducing apoptosis. In addition, as the reviewer suggested, we replaced original Fig 2B with revised one that includes the basal level of cytokines in THP-1 cells without Stx2a. There is no significant effect on cytokine induction by the treatment of OSMI-1 alone. Furthermore, OSMI-1 treatment did not change global transcriptome expressions in HRPTEpi cells (Fig EV4B).*

As the reviewer considered, we applied Tunicamycin as a different type of stimuli for ER-stress to THP-1 cells. Unexpectedly, once cells were exposed to Tunicamycin instead of Stx to induce ER-stress, O-GlcNAc levels were increased again, similar to Stx exposure, and the treatment of OSMI-1 could reduce the Tunicamycin-medicated LDH release and cytokine production. Although these results are very interesting and could suggest being widely applied to various diseases mediated by ER stress, it should be needed further study to verify it. Thus, we carefully decided not to deal with it in this paper, but the results are attached below for the reviewers' reference.

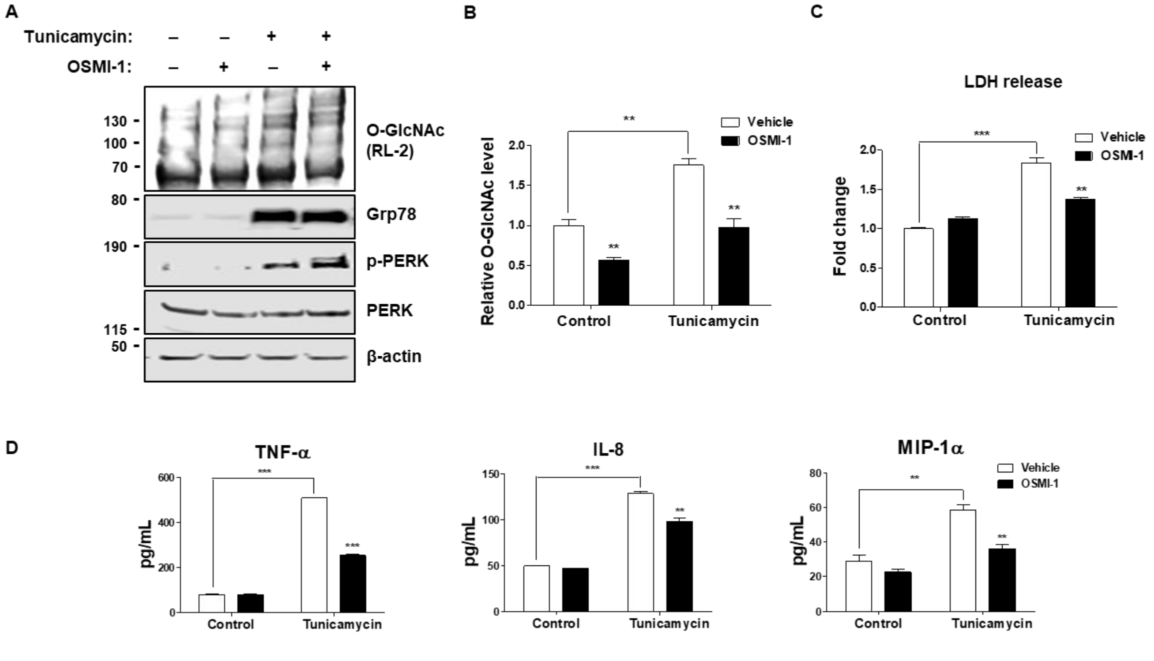

A. Representative western blot showing changes in overall O-GlcNAcylation and various ER stress markers in THP-1 cells treat ed with Tunicamycin (5 µg/mL) for 3 h in the presence or absence of O-linked *N*-acetylglucosamine transferase (OGT) inhibitor OSMI-1 (10 µM final).
B. Quantification of the band intensities for O-GlcNAc levels in (A). Data are presented as mean ± S.E.M. (n = 3, two-tailed Stud ent's *t*-test) normalized against β-actin, which was used as a loading control.
C. LDH cytotoxicity assay performed at 3 h following Tunicamycin (5 µg/mL) treatment of THP-1 cells in the presence or absence of OSMI-1 (10 µM final). Data are presented as mean ± S.E.M. (n = 3, two-tailed Student's t-test).
D. ELISAs were used to analyze the inhibitory effect of OSMI-1 on cytokine/chemokine production from THP-1 cells exposed to Tunicamycin (5 µg/mL) for 3 h in the presence or absence of OSMI-1. Data are presented as mean ± S.E.M. (n = 3, two-tailed Student's t-test).

2. In addition, ER stress markers need to be validated in the immunoblots.

***Response**: We newly added as the reviewer recommended, the validated results for ER stress markers such as PERK, Grp78, and IRE1α (Fig 1C). As already known, expressions of Grp78 or phosphorylation form of PERK or IRE1α were increased by Stx2a intoxication, which indicates that Stx potently induces ER stress in exposed cells. However, OSMI-1 treatment does not affect the expression of XBP1s, GFAT1 as well as ER stress markers (Fig 1C).*

Minor points:

1. Citation needed for "induction page 1, second line from the bottom".

***Response**: We appreciated the reviewer for this comment. We agree. A few references have been added to "introduction page 1" in the revised manuscript.*

Majowicz SE, Scallan E, Jones-Bitton A, Sargeant JM, Stapleton J, Angulo FJ, Yeung DH, Kirk MD (2014) Global incidence of human Shiga toxin-producing Escherichia coli infections and deaths: a systematic review and knowledge synthesis. Foodborne Pathog Dis 11: 447-55

Tesh VL (2012) Activation of cell stress response pathways by Shiga toxins. Cell Microbiol 14: 1-9

2. Page 4: line 3 from the bottom: "small G protein" to "small GTPase".

***Response**: As suggested by the reviewer, we have changed "small G protein" to "small GTPases"*

3. the label of "Stx2a-/-" should be changed to clearly label it as a mutant, as "Stx2a-/-" could be confusing with knockout.

***Response**: We agree. To avoid confusion, we changed "$Stx2a^{-/-}$" to "$Stx2a^{mut}$" throughout the revised manuscript.*

4. Page 6, last two lines change to "suggest" instead of "indicate", as there is no evidence on bacterial infection, only data on toxins.

***Response**: As suggested by the reviewer, we have changed "indicate" to "suggest".*

5. Page 12, line 4: should be "3D"?

***Response**: We respectively disagree. The meaning of the "…made using 2D cell culture…" in line 5 from the top on page 13 in the revised manuscript was clearly confirmed in the 3D culture system, demonstrating pathogenic mechanisms in host cells exposed to Stx are controlled through the regulation of O-GlcNAcylation.*

6. What is the rationale of choosing 132.5 ng/Kg dose?

***Response:** Since we basically used relatively young 4-week-old mice instead of 10- to 12-week-old mice mainly used in previous studies, a preliminary experiment was first performed to confirm the lethal dose of Stx2a close to 50% survival. As a result, when Stx2a was intraperitoneally injected at a concentration of 132.5ng/kg to mice, survival rate of 20% was*

consistently confirmed between days 4 and 5. Because a concentration of 220-290ng/kg in the papers we referenced was used, we started with 265ng/kg, and it has become to use a specific concentration of 132.5ng/kg in the process of diluting it by half.

Answers and revisions to the comments of reviewer #2:

Reviewer's Comments on Novelty/Model System for Author:

Overall this manuscript describes a unique finding that occurs in cells after exposure to Shiga toxins that appears to contribute to the cellular response to the toxin. The study is fairly complete (with one additional control desired). They even took the inhibitor into an animal model and appeared to get positive results, though the statistics were not reported.

Response: *As the reviewer considered, the statistical analyses were done to clarify the significance of in vivo study using mice, as shown in Figures 7B, C and EV5A.*

Reviewer's Remarks for Author:

The manuscript demonstrates that undifferentiated THP-1 cells, differentiated THP-1 cells, and HRPTEpi cells show elevated O-GlcNAcylation in response to treatment with Stx2a, but not to an Stx2a enzymatic mutant. Intriguingly, the enzyme (OGT) responsible for O-GlcNAcylation was not elevated upon Stx2a exposure. Rather, upstream enzyme GFAT1 was increased. Therefore, the authors propose that there is more substrate for OGT available, which leads to the increased O-GlcNAcylation. The elevated O-GlcNAcylation could be reduced with OSMI-1 treatment or inhibitory RNA. The increase in O-GlcNAcylation was correlated with retrograde transport of Stx2a and with the ER stress response. Additionally, the authors conclude that the increased O-GlcNAcylation is what leads to the reduced p-Akt and increased p-p65 after Stx2a treatment, but that link was not definitively shown. Finally, the OGT inhibitor was tested in spheroid and organoid models and showed the capacity to reduce elevations in cytokine responses after Stx2a exposure. There was a lack of controls with OSMI-1 alone overall; this lack was surprising since the authors spent time in the discussion describing the different affects that O-GlcNAcylation may have in different cell types. A control of OSMI-1 treated THP-1 cells at least is appropriate, and could be reported in the text if there are no differences. The animal data was suggestive, but no statistics were shown for those data, so the reader must assume the survival curves and clinical parameters were not different. It will be interesting in the future to see if OSMI-1 or another O-GlcNAcylation inhibitor could be used as a treatment in an animal model rather than as a preventative therapy.

1. Additionally, the authors conclude that the increased O-GlcNAcylation is what leads to the reduced p-Akt and increased p-p65 after Stx2a treatment, but that link was not definitively shown.

Response: *As described in the 3rd paragraph of the Discussion section, since the abnormal increase in O-GlcNAcylation level by Stx probably directly (or indirectly) affected the function or stability of many target proteins at a time, we attempted to analyze the phenotype by connecting to the global effect. Nevertheless, some proteins such as Akt and p65,*

known to be important in the induction of cell apoptosis and inflammation by Stx in previous studies, should be emphasized because they are also well-recognized as the target proteins for O-GlcNAcylation.

In the original manuscript, it was confirmed that the phosphorylation of Akt and p65 was regulated in connection with global O-GlcNAcylation levels (Fig 3A-D), and these two proteins were also directly modified by O-GlcNAcylation in the cell system we used (Fig EV3B).

In the revised manuscript, we performed additional experiments to supplement the mechanistic link for Akt, as the reviewer suggested. Once Akt with mutation for O-GlcNAcylation sites, Thr305 and Thr312, was overexpressed, cells were more resistant to apoptosis under Stx exposure than those with wild-type form due to the lack of induction for phosphorylation at Thr308 (Fig 3G-I). We believe that this result more directly proved that the apoptotic effect of Akt through phosphorylation at Thr308 is regulated depending on O-GlcNAcylation status at its adjacent sites, Thr305 and Thr312.

In the case of p65, approximately ten O-GlcNAcylation sites were found, but a site directly related to serine 536, a phosphorylation site, has not yet been clearly identified. Therefore, although further studies in the future are warranted to elucidate the precise mechanism by which every ten O-GlcNAcylation sites were regulated under Stx exposure, we decided not to deal with it because defining the relationship between O-GlcNAcylation and phosphorylation in p65 by mutating all O-GlcNAcylation sites may be beyond the scope of the present study and also would not fit for the purpose of our paper.

2. There was a lack of controls with OSMI-1 alone overall; this lack was surprising since the authors spent time in the discussion describing the different affects that O-GlcNAcylation may have in different cell types. A control of OSMI-1 treated THP-1 cells at least is appropriate, and could be reported in the text if there are no differences.

Response: *Basically, the treatment of OSMI-1 alone does not affect apoptotic or pro-inflammatory responses. Because cells were pre-treated with OSMI-1 for 12h before Stx exposure, no increase of either cleaved caspase-3 (Fig 1E and G) or fluorescent cells of the TUNEL assay (Fig 1H) at 0 h time-point under Stx2a plus OSMI-1 condition indicated that the treatment of OSMI-1 alone has no significant effect on inducing apoptosis. In addition, as the reviewer suggested, we replaced the original Fig 2B with a revised one that includes the basal level of cytokines in THP-1 cells without Stx2a. The revised Fig 2B shows that there is no significant effect on cytokine induction by the treatment of OSMI-1 alone. Furthermore, OSMI-1 treatment did not change global transcriptome expressions in HRPTEpi cells (Fig EV4B).*

3. The animal data was suggestive, but no statistics were shown for those data, so the reader must assume the survival curves and clinical parameters were not different.

Response: *As reviewer considered, the statistical analyses were done to clarify the significance of in vivo study using mice, as shown in Figures 7B, C and EV5A.*

Additional concerns

1. The authors state that severe clinical symptoms of mice were improved (page 5). No stats were done, so this is not clear.

Response: *As the reviewer considered, the statistical analyses were carried out to clarify the significance of in vivo study using mice, as shown in Figures 7B, C, and EV5A.*

2. Page 6 - both XBP1 and GFAT were elevated-presumably linked, but not shown by this study, rather, based on Wang reference

Response*: To supplement the mechanistic link, as reviewer considered, we performed additional experiments. Since the splicing of XBP1 is known to be induced by IRE1α activation under ER stress, we examined the inhibitory effect for XBP1 splicing by the treatment of IRE1α inhibitor (MKC-3946). As expected, the splicing of XBP1 induced by Stx2a exposure in host cells is inhibited under the treatment of IRE1α inhibitor, which suppresses GFAT1 expression and consequent increase in O-GlcNAcylation level, as shown in Fig 1C.*

3. Also, since no bacteria were used, and since EHEC express other factors, including those injected by a type III secretion system, the hypothesis should be about toxin, not bacteria. Speculation can be in the discussion.

Response*: The reviewer is correct. Our hypothesis should be about the toxin, not EHEC infection. Therefore, we changed to "…following the intoxication by Stx2a" at the end of the first result section on page 7 in the revised version. Typically, the enteric E.coli such as EHEC or EPEC have a conserved type 3 secretion system essential for their virulence and inject between 25 and 50 bacterial effector molecules. The following text has been added to the end of the discussion section: " Although there have been no reports describing the involvement of EHEC-produced effector molecules delivered via the type 3 secretion system for regulating O-GlcNAcylation in the infected mammalian host cells, the increased knowledge of the O-GlcNAcylation mediated-positive or negative pathways regulated by bacterial effector molecules translocated into the infected host may be a future experimental approach to provide additional promising therapeutic targets to prevent HUS disease progression."*

4. Page 7. OSMI-1 reduced the elevated O-GlcNAcylation, but did not "suppress" it.

Response*: We have changed "suppress" to "reduce", as reviewer suggested.*

5. Fig EV2A - the OSMI-1 treatment is significant, but modest.

Response*: We omitted "significantly" from the description in the text for the fluorescent image, as pointed out by the reviewer (page 8; line 8). As an additional interpretation, since the exact quantitative value for this TUNEL assay was clearly verified through FACS experiments (Fig 1H), these fluorescence images were presented as auxiliary visual data, not for quantitative purposes. Considering this, as you can see in Fig EV2A, more cells were observed under OSMI-1 treatment in the image at the same magnification because the cells are resistant to Stx2a exposure compared to the control. Still, there are fewer fluorescently stained cells indicative of apoptotic cells.*

6. Not clear why Stx1a was suddenly brought in? It is not a completely different toxin type-it traffics similarly and has the same mechanism of action as Stx2a.

Response: *As stated by the reviewer, Stx1a and Stx2a subtypes are the most commonly associated with causing pathogenesis in humans. However, they have different toxicity levels in animal models of disease and have different receptor preferences (Karve SS, Weiss AA. 2014. Glycolipid binding preferences of Shiga toxin variants. PLoS One. 9: e101173). Although the present studies have focused on the response of host cells to Stx2a since epidemiological studies have implicated infection with Stx2a-produdcing bacteria to be more likely to progress acute renal injury such as extraintestinal complications, the main purpose of using Stx1a is to verify the elevated O-GlcNAcylation with intoxication by another major subtype of Stx. Therefore, this is the supplementary information as presented in Fig EV2C.*

In addition, in the title and elsewhere, we changed "Shiga toxins-mediated" to "Shiga toxin-mediated". It would seem like the use of the plural would be correct, but here we are using "Shiga toxin" to refer to the entire family of toxins.

7. Figure 2 - what are the no toxin or OSMI alone control levels?

Response*: As suggested by the reviewer, a control panel showing the basal levels of secreted cytokines without Stx2a exposure, according to the presence or absence of OSMI-1 treatment, was added to Fig 2B. There was no significant effect on cytokine induction by the treatment of OSMI-1 alone.*

8. Figure 3B - do statistical comparisons for OSMI-1 treatment with and without Stx2a-should help distinguish if the treatment restores the levels of the proteins-it looks like it for p65 and perhaps p-Akt (though it does not look like it looking at the blots themselves??), but not p-Bad

Response*: As the reviewer suggested, the statistical comparisons were newly added for changes in the presence or absence of Stx2a under OSMI-1 treatment (Fig 3B). According to the statistic analyses, as the reviewer pointed out, p-Akt and p-p65 levels were restored close to the ones without Stx2a treatment, but it seemed not fully recovered at the p-Bad level. However, what to consider here is that Akt and p65 are direct target proteins of O-GlcNAcylation, but Bad is just one of several proteins whose activity is regulated downstream of Akt. Indeed, although the phosphorylation level of Akt (or p65) can be directly and sensitively regulated depending on the change of O-GlcNAcylation level as confirmed in Fig 3A-D, Bad must be regulated indirectly through Akt. Thus, it is presumed that p-Bad may be recovered at a different time-point from that of Akt or difficult to see a complete recovery. Nonetheless, it is considered very important that the phosphorylation of Bad, which was appreciably reduced by exposure to Stx2a, was restored to a statistically significant level under OSMI-1 treatment.*

9. RNA-seq, no OSMI-1-alone control - what does OSMI do to cells on its own.

Response*: As suggested by the reviewer, OSMI-1 alone control was newly added in Fig EV4B. As expected, OSMI-1 alone does not significantly affect the expression of factors related to inflammation or apoptosis, but those of the expression levels increased by Stx2a exposure can be effectively inhibited by OSMI-1 treatment (Fig 4F).*

10. P9 how do we know the O-GlcNAcylation leads to the phosphorylation changes? Figure 3A-OSMI1 treatment alone does not lead to an increase in p-p65, for example.

***Response**: In principle, phosphorylation and O-GlcNAcylation cross-talk to regulate protein function by occurring on the same or adjacent sites. In the case of Akt, phosphorylation at Thr308 is reciprocally regulated depending on nearby O-GlcNAcylation sites, Thr305 and 312, but phosphorylation of p65 at Ser536 is coincidentally induced while O-GlcNAcylation is increased. To support this mechanistic link, we performed additional experiments for Akt. Once Akt with mutation for O-GlcNAcylation sites, Thr305 and Thr312, was overexpressed, cells were more resistant to apoptosis under Stx exposure compared to those with wild-type form due to the lack of induction for phosphorylation at Thr308 (Fig 3G-I). We believe that this result more directly proved that the apoptotic effect of Akt through phosphorylation at Thr308 is regulated depending on O-GlcNAcylation status at its adjacent sites, Thr305 and Thr312.*

In addition, since OSMI-1 treatment was expected to lead to a decrease of phosphorylation level for p65 induced by Stx2a exposure, our result in Fig 3A that OSMI-1 alone cannot increase the phosphorylation of p65, as the reviewer indicated, seems correct. Conversely, it could be expected that the phosphorylation level of p65 would be lowered further under the treatment of OSMI-1 alone, but it was also difficult to confirm it because the basal level of p65 phosphorylation was too low, almost not detectable without Stx2a exposure.

11. Does the increase in p-p65 and decrease in p-Bad occur after the O-GlcNAcylation starts? Or do you see those changes as early as you see the increase in O-GlcNAcylation - 3-6 hours based on Figure 1. Certainly the change in Caspase is observed by 3 hours (Figure 1D).

***Response**: As the reviewer considered, it was confirmed that phosphorylation level for Akt or Bad was already decreased at 3 h, as early as caspase-3 was activated, after Stx2a exposure as shown in Fig EV3A. However, we could not observe any changes for phosphorylation of p65 yet at that time point.*

12. P10 not clear that these results show that the O-GlcNac inhibition works through Akt and p65

***Response**: As described in the 3rd paragraph of the Discussion section, since the abnormal increase in O-GlcNAcylation level by Stx probably directly (or indirectly) affected the function or stability of many target proteins at a time, we tried to analyze the phenotype by connecting to the global effect. Nevertheless, some proteins such as Akt and p65, known to be important in the induction of cell apoptosis and inflammation by Stx in previous studies, should be emphasized because they are also well-recognized as the target proteins for O-GlcNAcylation.*

In the original manuscript, it was confirmed that the phosphorylation of Akt and p65 was regulated in connection with global O-GlcNAcylation levels (Fig 3A-D), and these two proteins were also directly modified by O-GlcNAcylation in the cell system we used (Fig EV3).

In the revised manuscript, we performed additional experiments to supplement the mechanistic link for Akt, as the reviewer suggested. Once Akt with mutation for O-GlcNAcylation sites, Thr305 and Thr312, was overexpressed, cells were more resistant to apoptosis under Stx exposure than those with wild-type form due to the lack of induction for phosphorylation at Thr308 (Fig 3G-I). We believe that this result more directly proved that the apoptotic effect of Akt through phosphorylation at Thr308 is regulated depending on O-GlcNAcylation status at its adjacent sites, Thr305 and Thr312.

13. The authors should consider include the following paper in the discussion: Parello et al. 2015. Shiga toxin2-induced endoplasmic reticulum stress is minimized ... Toxins. 7

***Response**: We appreciated the reviewer for this kind suggestion. It has been added to the second sentence in the discussion of the revised manuscript.*

Parello CSL, Mayer CL, Lee BC, Motomochi A, Kurosawa S, Stearns-Kurosawa DJ. Shiga Toxin 2-Induced Endoplasmic Reticulum Stress Is Minimized by Activated Protein C but Does Not Correlate with Lethal Kidney Injury. Toxins. 2015; 7(1):170-186.

Other comments

1. More references are needed in a few places. For example, second sentence of the introduction and at the end of the 7th line of the results.

***Response**: We appreciated the reviewer for this comment. A few references have been added to the revised manuscript. Newly added references were highlighted by shading with yellow.*

2. The introduction, though informative, is rather long and could be shortened without losing relevant background. Additionally, the reader is left wondering why the authors chose to look at this specific post-translational modification?

***Response**: We modified introduction section to shorten it as the reviewer recommended. Word counts have now been reduced to 830 from 932 counts in the revised manuscript.*

***Response**: As described in the first paragraph of the result section, since it was already well-known that inflammation and apoptosis by Stx are induced through a potent ER stress response, and also O-GlcNAc modification is shown to be increased under ER stress condition, we attempted to determine whether the ER stress response could link Stx-mediated diseases and O-GlcNAcylation. To help readers understand, as the reviewer concerned, additional experimental results (Fig 1C) and explanations have been newly added to that part.*

3. Not clear why the Stx2a"-/-"designation is used for the holotoxoid? There are 3 mutations. Just Stx2a- would be sufficient, or Stx2a-/-/- if the desire is to indicate the # of mutations.

***Response**: We agree. To avoid confusion, we changed "$Stx2a^{-/-}$" to "$Stx2a^{mut}$" throughout the revised manuscript.*

4. Page 6. Figure 1 panel A shows levels of O-GlcNAcylation, not ER stress response.

***Response**: Fig 1A definitely shows the levels of cellular O-GlcNAcylation, but we wanted to emphasize our hypothesis that change of O-GlcNAcylation was probably mediated by ER stress response under Stx2a exposure. In contrast to the wild-type form of Stx2a, the O-GlcNAcylation level was barely induced in cells exposed to an enzymatic mutant form of Stx2a ($Stx2a^{mut}$) which triggers a minimal ER stress response (Fig 1A, right panel). In addition, intoxication with Stx2a did not elevate the O-GlcNAc level in THP-1 cells in the presence of Retro-2, which prevents ER stress by inhibiting retrograde trafficking of Stxs from the Golgi to the ER (Secher et al, 2015) (Fig EV1, upper panel). Furthermore, to supplement the mechanistic link between O-GlcNAcylation and ER stress, we performed additional experiments. Since the splicing of XBP1 is known to be induced by IRE1α activation under ER stress, we examined the splicing inhibitory effect for XBP1 by the treatment of IRE1α inhibitor (MKC-3946). As expected, the splicing of XBP1 induced by Stx2a exposure in host cells is inhibited under the treatment of IRE1α inhibitor, which suppresses GFAT expression and consequent increase in O-GlcNAcylation level, as shown in Fig 1C. Taken together, increased O-GlcNAcylation under Stx exposure may be mediated through ER stress response activated by Stx.*

5. Figure 1 figure legend (page 34)-should describe what is shown from top to bottom, not bottom to top-- O-GlcNAcylation should come before phosphorylation. Panel A before Panel C and so on.

Response: *We revised the legend for Figure 1, considering the order of data presentation.*

Separately, it was confirmed that the order of the sub-figures in Fig 3 in the original manuscript did not match those in the text, so this part was also corrected in the revised manuscript.

6. Figure 1C it would be nice to see an earlier time point, say 3 hours, for the XBP1 and GFAT1-since presumably an increase in those factors would precede the increase in O-GlcNAcylation that is detected by 6 hours.

Response*: As the reviewer suggested, we performed a new experiment to detect XBP1 and GFAT1 at 3 h after Stx2a exposure. As a result, induced levels of spliced XBP1 and GFAT1 were clearly detectable at 3 h. In addition, to supplement the mechanistic link between XBP1 and GFAT1, we examined the splicing inhibitory effect for XBP1 by the treatment of IRE1α inhibitor (MKC-3946) because the splicing of XBP1 is known to be induced by IRE1α activation under ER stress. As expected, the splicing of XBP1 caused by Stx2a exposure in host cells is inhibited under the treatment of IRE1α inhibitor, which suppresses GFAT expression and consequent increase in O-GlcNAcylation level, as shown in Fig 1C.*

7. Figure EV2A - Panels A and B description - panels show 4 time points, not just 9 h.

Response*: We thank the reviewer for this comment. We corrected it in the figure legend of EV2A in the revised version.*

Answers and revisions to the comments of reviewer #3:

Reviewer's Comment:

This is an interesting paper that reports that inhibiting the OGT enzyme responsible for glycosylating proteins on Ser/Thr residues with GlcNAc can reduce cell injury and mouse lethality due to Shiga toxin. The data could eventually inform therapeutic approaches to use OGT inhibition to reduce Shiga toxin pathology in the context of enterohemorrhagic E. coli infections. The following issues should be corrected and/or clarified:

1. Abstract - change 'generated' to 'produced'; change 'fine regulation' to 'inhibition'

Response*: We appreciated the reviewer for this kind suggestion. We rephrased them to 'produced' and 'inhibition', respectively, in the revised manuscript.*

2. Results - need a reference for the statement that the Stx2a mutant triggers a limited ER stress response

Response*: Reviewer is correct. The following references have been added in page 6:*

Sang-Yun Lee, Moo-Seung Lee, Rama P. Cherla, Vernon L. Tesh. Shiga toxin 1 induces apoptosis through the endoplasmic reticulum stress response in human monocytic cells. Cellular Microbiology, 2008, 2008 Mar;10(3):770-80. doi: 10.1111/j.1462-5822.2007.01083.x.

Park, Jun-Young, Yu-Jin Jeong, Sung-Kyun Park, Sung-Jin Yoon, Song Choi, Dae G. Jeong, Su W. Chung, Byung J. Lee, Jeong H. Kim, Vernon L. Tesh, Moo-Seung Lee, and Young-Jun Park. 2017. "Shiga Toxins Induce Apoptosis and ER Stress in Human Retinal Pigment

Epithelial Cells" Toxins 9, no. 10: 319. https://doi.org/10.3390/toxins9100319

3. Results, first paragraph - it is not UPD-GlcNAc production that is necessarily upregulated. This is an awkward statement. Presumably what the others mean at this point in the paper is that the Fig. 1 phenotype could be due to increased OGT activity or reduced OGA activity

***Response**: Although the UDP-GlcNAc level was not directly examined in our study, OGT expression was not changed in the cells exposed to Stx2a, but the expression of GFAT1, a rate-limiting enzyme of hexosamine biosynthetic pathway (HBP), was improperly increased (Fig 1C). Therefore, it was predicted that increased GFAT1 could elevate UDP-GlcNAc, a substrate of OGT, production through HBP and then induce cellular O-GlcNAcylation. In addition, when the activity of IRE1a, which is expected to induce GFAT1 expression as an upstream molecule, was inhibited, both the spliced XBP1 and GFAT1 expressions were not induced even when exposed to Stx2a, and the level of O-GlcNAcylation was also not increased, as shown in Fig 1C. Thus, changes of O-GlcNAcylation, particularly in Stx-exposed cells, seem to be regulated depending on GFAT1 expression rather than OGT or OGA.*

4. Results - need a reference for OSMI-1 activity the first time it is described

***Response**: Reviewer is correct. The following references have been added in page 7:*

Ortiz-Meoz, R.F.; Jiang, J.; Lazarus, M.B.; Orman, M.; Janetzko, J.; Fan, C.; Duveau, D.Y.; Tan, Z.W.; Thomas, C.J.; Walker, S. A small molecule that inhibits OGT activity in cells. ACS Chem Biol 2015, 10, 1392-1397, doi:10.1021/acschembio.5b00004.

*Park SK, Zhou X, Pendleton KE, Hunter OV, Kohler JJ, O'Donnell KA, Conrad NK (2017) A Conserved Splicing Silencer Dynamically Regulates O-GlcNAc Transferase Intron Retention and O-GlcNAc Homeostasis. Cell Rep 20**:** 1088-1099*

5. Results - need a reference for Retro-2 activity the first time it is described

***Response**: The following reference was already existed for inhibitory activity of Retro-2 for retro-trafficking in the 9th line from the bottom of page 6.*

*Secher T, Shima A, Hinsinger K, Cintrat JC, Johannes L, Barbier J, Gillet D, Oswald E (2015) Retrograde Trafficking Inhibitor of Shiga Toxins Reduces Morbidity and Mortality of Mice Infected with Enterohemorrhagic Escherichia coli. Antimicrob Agents Chemother 59**:** 5010-5013*

6. Figure 1D - describe why both CTD110.6 and RL-2 antibodies were used.

***Response**: The most widely used O-GlcNAc specific antibodies are the monoclonal antibodies CTD110.6 and RL-2, but the RL-2 and CTD110.6 epitopes are detected in a different manner. While CTD110.6 was raised against position 5 Ser modified RNA polymerase II subunit I C-Terminal Domain YSPTS*PS, RL2 was from rat liver nuclear envelope proteins and found to recognize eight nuclear pore complex proteins, all of which are O-GlcNAc modified. Therefore, Since the CTD110.6 antibody has a broader target while RL2 exhibits high specificity, the similar results for O-GlcNAcylation levels obtained from the two antibodies are considered complementary and representative.*

7. Figure 1D - quantify the GlcNAc signals as done in Figs. 1A-B

***Response**: As the reviewer suggested, we added quantification data for Fig 1E compared to OSMI-1 treatment to vehicle control (Fig 1F).*

8. Figure 3A - the stated difference in GlcNAc signals is not apparent from the blot provided as representative data

***Response**: To avoid confusion, we added the quantitative result for O-GlcNAcylation level from three independent experiments in Fig 3B.*

9. Figure 3E - why are p65 data not shown?

***Response**: We appreciated the reviewer for this comment. We newly added the representative western blot image in Fig 3C and its quantification data in Fig 3D.*

10. Figure 7B - statistical analysis is needed

***Response**: As the reviewer suggested, the statistical analyses were done to clarify the significance of in vivo study using mice, as shown in Figure 7B. Statistical differences among groups were assessed using a log-rank test with GraphPad Prism software. A statistically significant p-value (0.03) compared to OSMI-1-injected mice with high-dose to vehicle control was verified.*

11. Figure 7C - statistical analysis is needed

***Response**: As the reviewer considered, the statistical analyses were done to clarify the significance of in vivo study using mice, as shown in Figure 7C. The amounts of blood urea nitrogen (BUN) and creatinine, which are renal toxicity markers detected in the blood, were significantly increased upon treatment with Stx2a but restored to almost normal levels upon co-treatment with OGT inhibitor OSMI-1. In addition, OSMI-1 also completely protected against thrombocytopenia (reduction in the platelet count) in mice challenged with Stx2a.*

==

The reviewers' comments have greatly helped improve and consolidate our results in the revised manuscript and should be appreciated with many thanks.

We hope that all these point-by-point replies and manuscript revisions can satisfy the reviewers and editors.

Sincerely yours,

Moo-Seung Lee, Ph.D. (Environmental Diseases Research Center)
Sung-Kyun Park, Ph.D. (Infectious Disease Research Center)

Korea Research Institute of Bioscience & Biotechnology

125 Gwahak-ro, Yuseong-gu, Daejeon 34141, Republic of Korea

Phone: +82-42-879-8282, Fax: +82-42-879-8498, E-mail: skpark@kribb.re.kr

5th Nov 2021

Dear Dr. Park,

Thank you for the submission of your revised manuscript to EMBO Molecular Medicine. I am pleased to inform you that we will be able to accept your manuscript pending the following final amendments:

1) Please address minor issues raised by referees #1 and #2.
2) In the main manuscript file, please do the following:
- In M&M, a statistical paragraph that should reflect all information that you have filled in the Authors Checklist, especially regarding randomization, blinding, replication.
- Raw data from large-scale datasets e.g. RNA sequencing should be deposited in one of the relevant databases and made freely available prior the publication of the manuscript. Use the following format to report the accession number of your data:

The datasets produced in this study are available in the following databases:
[data type]: [full name of the resource] [accession number/identifier] ([doi or URL or identifiers.org/DATABASE:ACCESSION])

Please check "Author Guidelines" for more information.
https://www.embopress.org/page/journal/17574684/authorguide#availabilityofpublishedmaterial
3) Source data: Please zipp source data for all EV Figures as one file. Source data for the main figures should remain as one file per figure.
4) Synopsis: Please check your synopsis text and image, revise them if necessary and submit their final versions with your revised manuscript. Please be aware that in the proof stage minor corrections only are allowed (e.g., typos).
5) For more information: There is space at the end of each article to list relevant web links for further consultation by our readers. Could you identify some relevant ones and provide such information as well? Some examples are patient associations, relevant databases, OMIM/proteins/genes links, author's websites, etc...
6) As part of the EMBO Publications transparent editorial process initiative (see our Editorial at http://embomolmed.embopress.org/content/2/9/329), EMBO Molecular Medicine will publish online a Review Process File (RPF) to accompany accepted manuscripts. This file will be published in conjunction with your paper and will include the anonymous referee reports, your point-by-point response and all pertinent correspondence relating to the manuscript. Let us know whether you agree with the publication of the RPF and as here, if you want to remove or not any figures from it prior to publication. Please note that the Authors checklist will be published at the end of the RPF.
7) Please provide a point-by-point letter INCLUDING my comments as well as the reviewer's reports and your detailed responses (as Word file).

I look forward to reading a new revised version of your manuscript as soon as possible.

Yours sincerely,

Zeljko Durdevic

Zeljko Durdevic
Editor
EMBO Molecular Medicine

***** Reviewer's comments *****

Referee #1 (Remarks for Author):

The revision is largely satisfactory. The authors found that ER-stress induced by Tunicamycin has similar impact on O-GlcNAc and can be inhibited by OSMI-1, suggesting that the effect is not specific to Stx2a. It will be better for authors to mention this possibility/limitation briefly in the discussion section.

Many references lack page numbers.

Referee #2 (Comments on Novelty/Model System for Author):

This manuscript shows clearly a new impact of Stx2 on the host cell. The appropriate controls were done, and additional statistical analyses were added as appropriate. The reason for the low medical impact is that even though they demonstrated good results in mice, it is not clear how to translate that into treatment in humans.

Referee #2 (Remarks for Author):

This is a review of the modified submission for EMM-2021-14678v2.
The authors addressed previous concerns adequately.

Minor issues:
Page 7 - "treatment of Stx2a" should be "with Stx2a"
Figure legends: check for extra "..." at the end of some figure legends

Referee #3 (Remarks for Author):

Is suitable for publication

EMBO Molecular Medicine

Dear Editor:

We have revised our manuscript (EMM-14678-V3), "**Inhibition of O-GlcNAcylation protects from Shiga toxin-mediated cell injury and lethality in host**".

We would like to thank all reviewers who improved the quality of our paper through very professional and meticulous evaluation.

Below, we summarized our revisions according to the comments from reviewers in a point-by-point way. The changed or newly-added portions have been highlighted by shading with yellow color in the revised manuscript.

==

Answers and revisions to the comments of reviewer #1:

(Remarks for Author):

1. The revision is largely satisfactory. The authors found that ER-stress induced by Tunicamycin has similar impact on O-GlcNAc and can be inhibited by OSMI-1, suggesting that the effect is not specific to Stx2a. It will be better for authors to mention this possibility/limitation briefly in the discussion section.

Response*: We newly described it as our unpublished data at the end of discussion section.*

2. Many references lack page numbers.

Response*: Thank you for this comment. We recognized that our reference list in the manuscript includes several papers published in MDPI (St Basel Anlage 66, 4052 Basel, Switzerland). According to their editorial policy (https://www.mdpi.com/authors/layout#_bookmark53), instead of using page range in the citation, these electronic journals have switched to article numbers, which assigns a single number to the entire paper. Nonetheless, it seemed our EndNote program was not applied it*

appropriately to our manuscript, so we revised it by ourselves.

Answers and revisions to the comments of reviewer #2:

(Comments on Novelty/Model System for Author):
This manuscript shows clearly a new impact of Stx2 on the host cell. The appropriate controls were done, and additional statistical analyses were added as appropriate. The reason for the low medical impact is that even though they demonstrated good results in mice, it is not clear how to translate that into treatment in humans.

Response*: We agree with the reviewer's consideration of that issue. Therefore, the significance and limitations of our study in order to be developed as a practical treatment were additionally explained in the discussion section as highlighted.*

(Remarks for Author):
This is a review of the modified submission for EMM-2021-14678v2.
The authors addressed previous concerns adequately.

Minor issues:
1. Page 7 - "treatment of Stx2a" should be "with Stx2a"

Response*: Thank you for this comment. We changed "treatment of Stx2a" to "treatment with Stx2a".*

2. Figure legends: check for extra "..." at the end of some figure legends

Response*: We deleted them in the revised manuscript.*

Answers and revisions to the comments of reviewer #3:

(Remarks for Author):
Is suitable for publication

Response*: We appreciate for this positive evaluation.*

===

We hope that all these point-by-point replies and manuscript revisions can satisfy the reviewers.

Sincerely yours,

Moo-Seung Lee, Ph.D. (Environmental Diseases Research Center)
Sung-Kyun Park, Ph.D. (Infectious Disease Research Center)

Korea Research Institute of Bioscience & Biotechnology

125 Gwahak-ro, Yuseong-gu, Daejeon 34141, Republic of Korea

Phone: +82-42-879-8282, Fax: +82-42-879-8498, E-mail: skpark@kribb.re.kr

We are pleased to inform you that your manuscript is accepted for publication and is now being sent to our publisher to be included in the next available issue of EMBO Molecular Medicine.

EMBO PRESS

YOU MUST COMPLETE ALL CELLS WITH A PINK BACKGROUND ↓

PLEASE NOTE THAT THIS CHECKLIST WILL BE PUBLISHED ALONGSIDE YOUR PAPER

Corresponding Author Name: SUNG-KYUN PARK
Journal Submitted to: EMBO Molecular Medicine
Manuscript Number: EMM-2021-14678-V3

Reporting Checklist For Life Sciences Articles (Rev. June 2017)

This checklist is used to ensure good reporting standards and to improve the reproducibility of published results. These guidelines are consistent with the Principles and Guidelines for Reporting Preclinical Research issued by the NIH in 2014. Please follow the journal's authorship guidelines in preparing your manuscript.

A- Figures
1. Data
The data shown in figures should satisfy the following conditions:

- the data were obtained and processed according to the field's best practice and are presented to reflect the results of the experiments in an accurate and unbiased manner.
- figure panels include only data points, measurements or observations that can be compared to each other in a scientifically meaningful way.
- graphs include clearly labeled error bars for independent experiments and sample sizes. Unless justified, error bars should not be shown for technical replicates.
- if n< 5, the individual data points from each experiment should be plotted and any statistical test employed should be justified
- Source Data should be included to report the data underlying graphs. Please follow the guidelines set out in the author ship guidelines on Data Presentation.

2. Captions
Each figure caption should contain the following information, for each panel where they are relevant:

- a specification of the experimental system investigated (eg cell line, species name).
- the assay(s) and method(s) used to carry out the reported observations and measurements
- an explicit mention of the biological and chemical entity(ies) that are being measured.
- an explicit mention of the biological and chemical entity(ies) that are altered/varied/perturbed in a controlled manner.

- the exact sample size (n) for each experimental group/condition, given as a number, not a range;
- a description of the sample collection allowing the reader to understand whether the samples represent technical or biological replicates (including how many animals, litters, cultures, etc.).
- a statement of how many times the experiment shown was independently replicated in the laboratory.
- definitions of statistical methods and measures:
  - common tests, such as t-test (please specify whether paired vs. unpaired), simple χ2 tests, Wilcoxon and Mann-Whitney tests, can be unambiguously identified by name only, but more complex techniques should be described in the methods section;
  - are tests one-sided or two-sided?
  - are there adjustments for multiple comparisons?
  - exact statistical test results, e.g., P values = x but not P values < x;
  - definition of 'center values' as median or average;
  - definition of error bars as s.d. or s.e.m.

Any descriptions too long for the figure legend should be included in the methods section and/or with the source data.

USEFUL LINKS FOR COMPLETING THIS FORM

http://www.antibodypedia.com
http://1degreebio.org
http://www.equator-network.org/reporting-guidelines/improving-bioscience-research-report
http://grants.nih.gov/grants/olaw/olaw.htm
http://www.mrc.ac.uk/Ourresearch/Ethicsresearchguidance/Useofanimals/index.htm
http://ClinicalTrials.gov
http://www.consort-statement.org
http://www.consort-statement.org/checklists/view/32-consort/66-title
http://www.equator-network.org/reporting-guidelines/reporting-recommendations-for-tumo
http://datadryad.org
http://figshare.com
http://www.ncbi.nlm.nih.gov/gap
http://www.ebi.ac.uk/ega
http://biomodels.net/
http://biomodels.net/miriam/
http://jjj.biochem.sun.ac.za
https://osp.od.nih.gov/biosafety-biosecurity-and-emerging-biotechnology/
http://www.selectagents.gov/

In the pink boxes below, please ensure that the answers to the following questions are reported in the manuscript itself. Every question should be answered. If the question is not relevant to your research, please write NA (non applicable). We encourage you to include a specific subsection in the methods section for statistics, reagents, animal models and human subjects.

B- Statistics and general methods

Please fill out these boxes ↓ (Do not worry if you cannot see all your text once you press return)

1.a. How was the sample size chosen to ensure adequate power to detect a pre-specified effect size?	We adhered to best practice guidelines established in our field of science and chose sample sizes accordingly.
1.b. For animal studies, include a statement about sample size estimate even if no statistical methods were used.	We used standard statistical methods for analyses in all animal experiments. N were estimaed based on our previous work
2. Describe inclusion/exclusion criteria if samples or animals were excluded from the analysis. Were the criteria pre-established?	We have not excluded samples or animals from our analyses. The criteria would have been pre established as is standard in the field.
3. Were any steps taken to minimize the effects of subjective bias when allocating animals/samples to treatment (e.g. randomization procedure)? If yes, please describe.	The animals/samples were randomly allocated into experimental groups. In addition, we made an effort to verify reproducibility as well as reduce biased interpretation through the participation of multiple researchers and repeated experiments more than three times with independent biological samples in animal studies and cell-based experimental analyses.
For animal studies, include a statement about randomization even if no randomization was used.	Randomization of animals was performed to allocate them to each treatment group, in order to minimize cage, litter or animal group differences. Mice was randomized to treatment groups before initiation of drug administration.
4.a. Were any steps taken to minimize the effects of subjective bias during group allocation or/and when assessing results (e.g. blinding of the investigator)? If yes please describe.	For animal experiments, mice were randomly distributed into three groups to minimize subjective bias. In addition, we made an effort to verify reproducibility as well as reduce biased interpretation through the participation of multiple researchers and repeated experiments more than three times with independent biological samples in animal studies and cell-based experimental analyses.
4.b. For animal studies, include a statement about blinding even if no blinding was done	There was no "blinding of the investigator" in conducting animal experiments. Instead, we attempted to minimize subjective bias by independently repeating the experiment using the same conditions by different researchers more than three times to check the reproducibility.
5. For every figure, are statistical tests justified as appropriate?	Yes.
Do the data meet the assumptions of the tests (e.g., normal distribution)? Describe any methods used to assess it.	Yes.
Is there an estimate of variation within each group of data?	Yes.
Is the variance similar between the groups that are being statistically compared?	Yes.

C- Reagents

6. To show that antibodies were profiled for use in the system under study (assay and species), provide a citation, catalog number and/or clone number, supplementary information or reference to an antibody validation profile. e.g., Antibodypedia (see link list at top right), 1DegreeBio (see link list at top right).	1. RL2 (1:5000, MA1-072, ThermoFisher Scientific)<br>2. CTD110.6 (1:3000, sc-59623, Santa Cruz Biotechnologies)<br>3. XBP1 (1:1000, sc-8015, Santa Cruz Biotechnologies)<br>4. GFAT1 (1:1000, sc-377479, Santa Cruz Biotechnologies)<br>5. p65 (1:3000, AB_10859369, Cell Signaling Technology)<br>6. OGT (1:3000, AB_532313, MilliporeSigma/Merck KGaA)<br>7. phospho-IRE1a (1:1000, ab48187, abcam)<br>8. PERK (1:1000, AB_2095847, Cell Signaling Technology)<br>9. phospho-PERK (1:1000, AB_2095853, Cell Signaling Technology)<br>10. Grp78 (1:3000, AB_10695864, Cell Signaling Technology)<br>11. IRE1a (1:1000, AB_823545, Cell Signaling Technology)<br>12. phospho-p65 (1:3000, AB_331284, Cell Signaling Technology)<br>13. Caspase-3 (1:3000, AB_2069872, Cell Signaling Technology)<br>14. Akt (1:3000, AB_915788, Cell Signaling Technology)<br>15. phospho-Akt (1:3000, AB_331163, Cell Signaling Technology)<br>16. Bad (1:3000, AB_331419, Cell Signaling Technology)<br>17. Phospho-Bad (1:3000, AB_560884, Cell Signaling Technology)<br>18. b-actin (1:5000, AB_1903890, Cell Signaling Technology)<br>19. Anti-mouse IgG (1:5000, 7076, Cell Signaling Technology)<br>20. Anti-rabbit igG (1:5000, 7074, Cell Signaling Technology)<br>21. Anti-mouse IgM (1:5000, ab97230, abcam)
7. Identify the source of cell lines and report if they were recently authenticated (e.g., by STR profiling) and tested for mycoplasma contamination.	1. Human monocytic THP-1 were purchased from ATCC.<br>2. Primary human proximal tubular epithelial cells were purchased from Cell Applications, Inc.<br>3. 3D human renal proximal tubular epithelial, 3D mini-kidney spheroids were purchased from ScienCell Research Laboratories<br>4. Human induced pluripotent stem cells were purchased from ATCC.

* for all hyperlinks, please see the table at the top right of the document

D- Animal Models

8. Report species, strain, gender, age of animals and genetic modification status where applicable. Please detail housing and husbandry conditions and the source of animals.	All details are provided in the materials and methods sections, figure legends, and main text.
9. For experiments involving live vertebrates, include a statement of compliance with ethical regulations and identify the committee(s) approving the experiments.	This is included in the materials and methods section.
10. We recommend consulting the ARRIVE guidelines (see link list at top right) (PLoS Biol. 8(6), e1000412, 2010) to ensure that other relevant aspects of animal studies are adequately reported. See author guidelines, under 'Reporting Guidelines'. See also: NIH (see link list at top right) and MRC (see link list at top right) recommendations. Please confirm compliance.	We confirmed compliance.

E- Human Subjects

11. Identify the committee(s) approving the study protocol.	Not applicable.
12. Include a statement confirming that informed consent was obtained from all subjects and that the experiments conformed to the principles set out in the WMA Declaration of Helsinki and the Department of Health and Human Services Belmont Report.	Not applicable.
13. For publication of patient photos, include a statement confirming that consent to publish was obtained.	Not applicable.
14. Report any restrictions on the availability (and/or on the use) of human data or samples.	Not applicable.
15. Report the clinical trial registration number (at ClinicalTrials.gov or equivalent), where applicable.	Not applicable.
16. For phase II and III randomized controlled trials, please refer to the CONSORT flow diagram (see link list at top right) and submit the CONSORT checklist (see link list at top right) with your submission. See author guidelines, under 'Reporting Guidelines'. Please confirm you have submitted this list.	Not applicable.
17. For tumor marker prognostic studies, we recommend that you follow the REMARK reporting guidelines (see link list at top right). See author guidelines, under 'Reporting Guidelines'. Please confirm you have followed these guidelines.	Not applicable.

F- Data Accessibility

18: Provide a "Data Availability" section at the end of the Materials & Methods, listing the accession codes for data generated in this study and deposited in a public database (e.g. RNA-Seq data: Gene Expression Omnibus GSE39462, Proteomics data: PRIDE PXD000208 etc.) Please refer to our author guidelines for 'Data Deposition'.<br><br>Data deposition in a public repository is mandatory for:<br>a. Protein, DNA and RNA sequences<br>b. Macromolecular structures<br>c. Crystallographic data for small molecules<br>d. Functional genomics data<br>e. Proteomics and molecular interactions	This study includes no data deposited in external repositories.
19. Deposition is strongly recommended for any datasets that are central and integral to the study; please consider the journal's data policy. If no structured public repository exists for a given data type, we encourage the provision of datasets in the manuscript as a Supplementary Document (see author guidelines under 'Expanded View' or in unstructured repositories such as Dryad (see link list at top right) or Figshare (see link list at top right).	Not applicable.
20. Access to human clinical and genomic datasets should be provided with as few restrictions as possible while respecting ethical obligations to the patients and relevant medical and legal issues. If practically possible and compatible with the individual consent agreement used in the study, such data should be deposited in one of the major public access-controlled repositories such as dbGAP (see link list at top right) or EGA (see link list at top right).	Not applicable.
21. Computational models that are central and integral to a study should be shared without restrictions and provided in a machine-readable form. The relevant accession numbers or links should be provided. When possible, standardized format (SBML, CellML) should be used instead of scripts (e.g. MATLAB). Authors are strongly encouraged to follow the MIRIAM guidelines (see link list at top right) and deposit their model in a public database such as Biomodels (see link list at top right) or JWS Online (see link list at top right). If computer source code is provided with the paper, it should be deposited in a public repository or included in supplementary information.	Not applicable.

G- Dual use research of concern

22. Could your study fall under dual use research restrictions? Please check biosecurity documents (see link list at top right) and list of select agents and toxins (APHIS/CDC) (see link list at top right). According to our biosecurity guidelines, provide a statement only if it could.	Not applicable.
